# A tissue-specific atlas of protein–protein associations enables prioritization of candidate disease genes

**DOI:** 10.1038/s41587-025-02659-z

**Published:** 2025-05-02

**Authors:** Diederik S. Laman Trip, Marc van Oostrum, Danish Memon, Fabian Frommelt, Delora Baptista, Kalpana Panneerselvam, Glyn Bradley, Luana Licata, Henning Hermjakob, Sandra Orchard, Gosia Trynka, Ellen M. McDonagh, Andrea Fossati, Ruedi Aebersold, Matthias Gstaiger, Bernd Wollscheid, Pedro Beltrao

**Affiliations:** 1 Department of Biology, Institute of Molecular Systems Biology, ETH Zurich, Zurich, Switzerland; 2 Swiss Institute of Bioinformatics, Lausanne, Switzerland; 3 Department of Health Sciences and Technology, Institute of Translational Medicine, ETH Zurich, Zurich, Switzerland; 4 European Molecular Biology Laboratory, European Bioinformatics Institute (EMBL-EBI), Wellcome Genome Campus, Cambridge, UK; 5 Open Targets, Wellcome Genome Campus, Cambridge, UK; 6 Gulbenkian Institute for Molecular Medicine, Oeiras, Portugal; 7 Computational Biology, Functional Genomics, GSK, Stevenage, UK; 8 Department of Biology, University of Rome Tor Vergata, Rome, Italy; 9 Wellcome Sanger Institute, Wellcome Genome Campus, Cambridge, UK; 10 Science for Life Laboratory, Department of Microbiology, Tumor and Cell Biology, Karolinska Institute, Solna, Sweden; 11 Present Address: Biozentrum, University of Basel, Basel, Switzerland

**Keywords:** Proteomic analysis, Computational biology and bioinformatics, Target identification

## Abstract

Despite progress in mapping protein–protein interactions, their tissue specificity is understudied. Here, given that protein coabundance is predictive of functional association, we compiled and analyzed protein abundance data of 7,811 proteomic samples from 11 human tissues to produce an atlas of tissue-specific protein associations. We find that this method recapitulates known protein complexes and the larger structural organization of the cell. Interactions of stable protein complexes are well preserved across tissues, while cell-type-specific cellular structures, such as synaptic components, are found to represent a substantial driver of differences between tissues. Over 25% of associations are tissue specific, of which <7% are because of differences in gene expression. We validate protein associations for the brain through cofractionation experiments in synaptosomes, curation of brain-derived pulldown data and AlphaFold2 modeling. We also construct a network of brain interactions for schizophrenia-related genes, indicating that our approach can functionally prioritize candidate disease genes in loci linked to brain disorders.

## Abstract (web summary)

Protein–protein interactions specific to 11 individual tissues are presented in a comprehensive resource.

## Main

Protein–protein interactions mediate the biophysical structure and functioning of the cell, with disruptions potentially leading to disease. Unraveling the interactions of human proteins has been a long-standing challenge^1^, with recent advances in mass spectrometry (MS) coupled with high-throughput screens such as protein-fragment complementation^2^, yeast two-hybrid^3^, affinity purification (AP)^4,5^ or cofractionation^6–8^ enabling the discovery of interactions on a proteome-wide scale. These experimental datasets are complemented by modern computational approaches that compile collections of interactions using machine-learning-based methods often trained with experimental evidence^1^. Together, these experimental and computational efforts are the basis for numerous publicly available, high-quality protein interaction databases^1,9–11^.

However, these databases typically aggregate interactions without specifying context, whereas the interactome is highly tissue or cell state specific, with less than half of the proteome being detected in all tissues^12^. Elucidating the tissue specificity of protein interactions is important for understanding cell-type-specific function, finding drug targets and developing a system-level understanding of the human cell. Indeed, specifying tissue specificity of the interactome has proven difficult. Initial attempts to generate tissue-specific interaction networks relied on gene expression data to identify coexpressed genes or to exclude proteins on the basis of a lack of expression in a given tissue^13,14^. However, the accuracy of predicting protein associations by mRNA coexpression is limited and it is unclear to what extent gene expression is the major driver of changes in protein interactions^15–18^. While there are some recent experimental efforts for establishing tissue-specific interactions on a proteome-wide scale^4,6^, such approaches require extensive resources even for a single tissue and often use immortalized cell lines or other models whose interactome may not appropriately represent the human tissue. One approach used changes in ratios for complex subunits from proteomic measurements to establish changes in protein interactions in a disease state^19^. An alternative approach used the coabundance of proteins for the purpose of establishing ‘protein associations’^5,17,20,21^. Here, associations are used instead of interactions to reserve the latter for physical interactions. Coabundance has been shown to be an accurate measure for predicting protein–protein associations likely because of the fact that protein complexes consist of subunits assembled in defined stoichiometries that are often coexpressed, with orphaned subunits often being degraded^15,22–25^. The accuracy of coabundance for predicting protein association and the rising number of large-scale proteomics studies of human cancers, with genetic heterogeneity underlying diverse cellular changes, offer a timely opportunity to systematically establish the tissue specificity of protein associations.

Here, we present a protein association atlas derived from the coabundance of proteins in 7,811 human biopsies, scoring the likelihood of 116 million protein associations across 11 tissues. This atlas recovers well-known protein complexes, maps relationships between and across traits and cellular components and proposes context-relevant associations for disease genes. Focusing on the brain and using interactions derived from orthogonal approaches, we illustrate the use of our association atlas for prioritizing genes and interactions of disease genes in a tissue-specific manner.

## Results

### Protein coabundance scores genome-wide protein associations

**Fig. 1:**
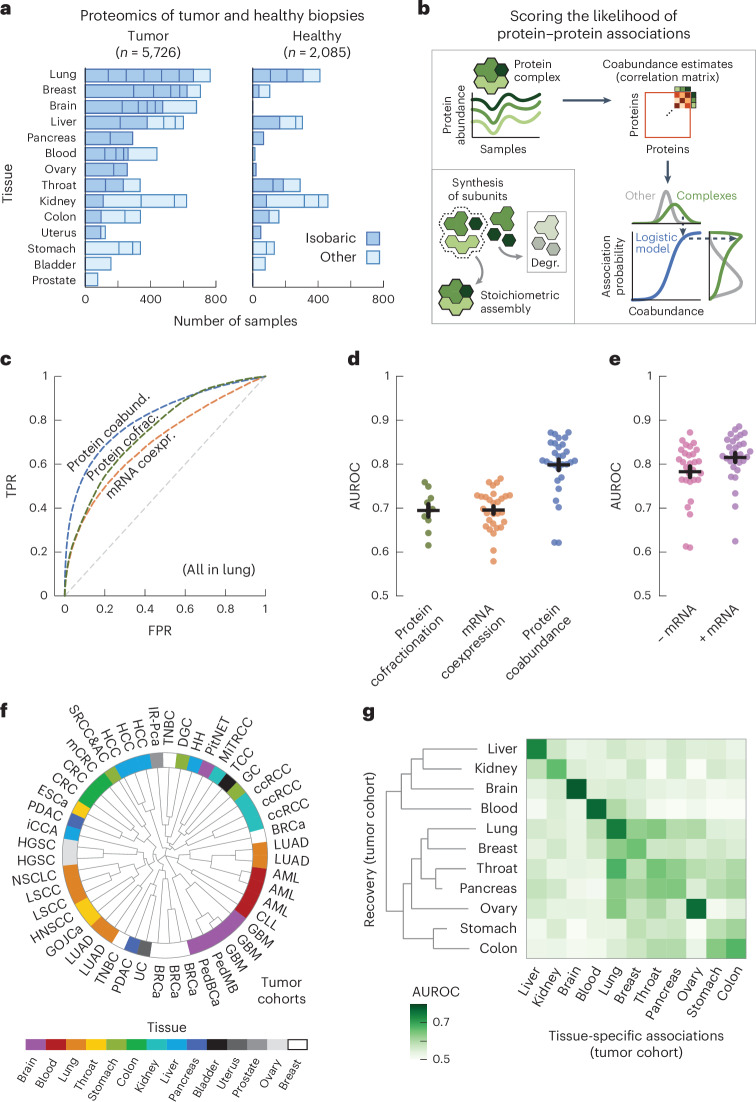
Protein coabundance outperforms mRNA coexpression and protein cofractionation for recovering protein–protein interactions on a genome-wide scale. **a**, Number of tumor and healthy samples per tissue. Bar sections indicate individual studies, using multiplexed proteomics with isobaric labeling (dark blue) or other methods (light blue). **b**, Schematic representation of workflow. Subunits of protein complexes occur in fixed stoichiometries. Protein coabundance is estimated through correlation of protein abundance profiles and converted to probabilities through a logistic model using interactions between subunits of protein complexes (CORUM) as positives. Degr., degradation. **c**, ROC curves for association probabilities in lung tissue derived from protein coabundance (coabund.; blue), mRNA coexpression (coexpr.; orange) and protein cofractionation (cofrac.; green). The gray dashed line shows the performance of a random classifier. FPR, false-positive rate; TPR, true-positive rate. **d**, AUC values for association probabilities as illustrated in **c**. Shown are studies that quantified both protein coabundance (blue; *n* = 29) and mRNA coexpression (orange; *n* = 29) or protein cofractionation (green; *n* = 10). **e**, AUC values for association probabilities derived from protein coabundance combined with mRNA coexpression through a linear model (purple) and protein coabundance after regressing gene expression out of the protein abundance (pink). Shown are the same studies as in **d**. In **d**,**e**, each dot represents one study with paired transcriptomics and proteomics data. Protein pairs were filtered for having association probabilities from both modalities. Error bars show the mean and s.e.m. In **c**–**e**, negatives are all quantified protein pairs not reported as complex members. **f**, Clustering of the *n* = 48 cohorts using association probabilities of protein pairs with the most variable associations (CV above the median). The radial dendrogram shows complete-linkage clustering with the Pearson correlation distance. Cohorts are labeled according to the type of cancer; colors represent the different human tissues. Leaf-joint distances were shortened. **g**, Heat map of AUCs for recovering tissue-specific associations with cohorts that were withheld when predicting these associations. Each square represents the average AUC for all cohorts of a given tissue. Tissues were clustered through complete-linkage clustering with the Manhattan distance. Source data

We started by collecting protein abundance data from proteomics studies of cohorts of participants with cancer. In total, we compiled a dataset of 50 studies across 14 human tissues, encompassing 5,726 samples of tumors and 2,085 samples of adjacent healthy tissue^26–75^ (Fig. 1a and Supplementary Table 1). We further included the mRNA expression data paired to the proteomics for 2,930 of the tumor and 722 of the healthy samples. Following previous studies, we used the fact that protein complex members are strongly transcriptionally and post-transcriptionally coregulated to compute probabilities of protein–protein associations from the abundance data^5,17,20^ (Fig. 1b, Methods and Supplementary Fig. 1). In short, we preprocessed the abundance data to obtain a log-transformed and median-normalized abundance across participants. For each study, we then computed a coabundance estimate of a protein pair as the Pearson correlation when both proteins were quantified in at least 30 samples (Supplementary Fig. 2). Lastly, with pairs of subunits for curated stable protein complexes as ground-truth positives (CORUM^76^), we used a logistic model for each study to convert the coabundance estimates to probabilities of protein–protein associations (Supplementary Figs. 3–5).

To test the ability of the association probabilities to recover known complex members, we computed receiver operating characteristic (ROC) curves for probabilities derived from protein coabundance, mRNA coexpression and protein cofractionation^6,7,77^ (Fig. 1c). We found that protein coabundance (area under the curve (AUC) = 0.80 ± 0.01 (mean ± s.e.m.)) outperformed protein cofractionation (AUC = 0.69 ± 0.01) and mRNA coexpression (AUC = 0.70 ± 0.01) data for recovering known interactions (Fig. 1d and Methods). In addition, the combination of mRNA and protein abundance data did not significantly improve the recovery of known complex members (Fig. 1e; AUC = 0.82 ± 0.01, *P* = 0.15, according to a one-sided Welch’s *t*-test). Therefore, with roughly half of all cohorts having paired mRNA expression data available, we chose to only use protein coabundance for computing association probabilities. Additionally, we found similar AUCs when regressing gene expression out of the protein abundance before computing protein coabundance estimates (AUC = 0.78 ± 0.01, *P* = 0.18), suggesting that post-transcriptional processes but not regulation of gene expression drive most of the predictive power for protein associations.

Having established that the association probabilities derived from protein coabundance data recover known interactions of protein complex members, we sought to test whether replicate studies of the same tissue yielded association probabilities that were representative for each tissue. As a starting point, we used the gene expression data to establish that the association probabilities were not driven by cell-type composition^78^ (Supplementary Fig. 6). Next, using the 1,115,405 association probabilities that were quantified for all studies, we found that the replicate cohorts from the same tissue generally clustered together (Fig. 1f; for example, blood, brain, liver and lung). Next, we selected the associations that were tissue specific, that is associations whose average probability exceeded the 95th percentile for a given tissue (0.68 ± 0.01 across tissues) and whose average probability remained below 0.5 across all other tissues. Through a hold-one-out methodology, we found that the tissue-specific associations were primarily recovered by cohorts of the same tissue of origin (AUC = 0.71 ± 0.01) compared to cohorts from different tissues (AUC = 0.56 ± 0.00, *P* < 0.05 for all tissues, according to a one-sided Welch’s *t*-test) (Fig. 1g, Methods and Supplementary Fig. 7). Together, these observations suggest that the tissue of origin is a major driver of differences between cohorts.

### An atlas of protein associations in human tissues

**Fig. 2:**
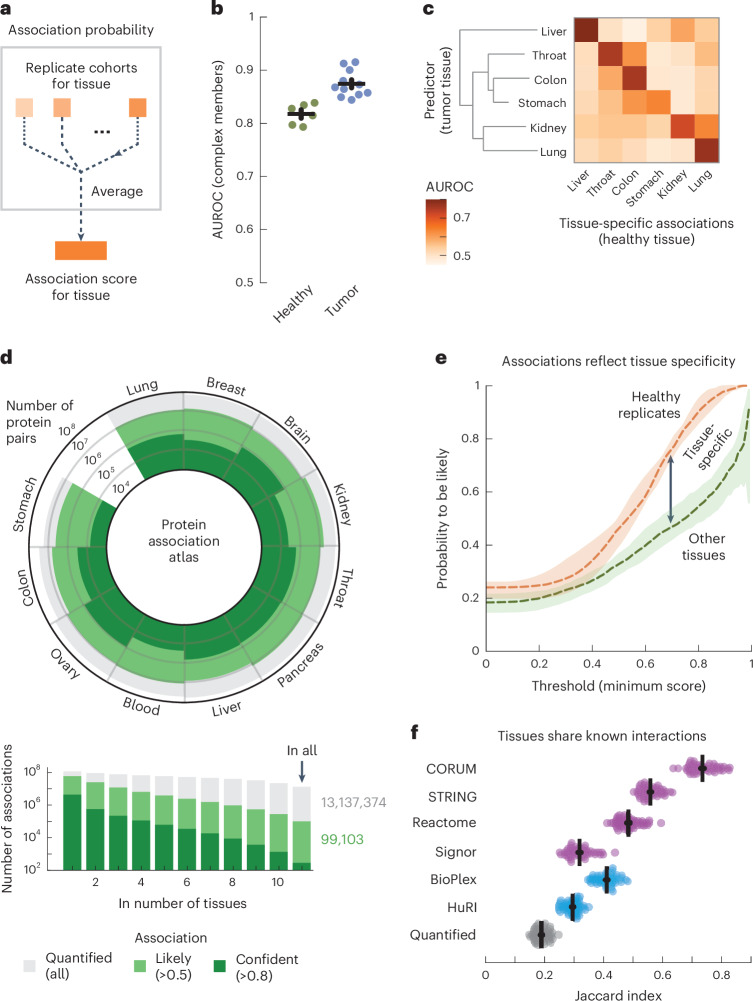
Association atlas scores likelihood of protein interactions across human tissues. **a**, Schematic for aggregating replicate cohorts into a single association score for a tissue. **b**, AUC values for the association scores derived from healthy samples (green; *n* = 6) and tumor samples (blue; *n* = 11), using interactions between subunits of protein complexes (CORUM) as positives. Association scores were filtered for protein pairs having probabilities in all cohorts of a tissue. **c**, Heat map of AUCs for using tumor-derived association scores to recover tissue-specific associations defined by the association scores from healthy tissues. Association scores only include cohorts that had both healthy and tumor samples. Tissues were clustered through complete-linkage clustering with the Manhattan distance. **d**, Atlas of protein associations in *n* = 11 human tissues. The radial diagram shows, for each tissue, the numbers of protein pairs that were quantified (gray), are likely to interact (light green; association score > 0.5) or were confidently quantified (dark green; association score > 0.8). The bar graph shows the number of associations that were quantified in the given number of tissues. **e**, Probability of associations of a tissue to likely be in a healthy-tissue-derived replicate (orange; *n* = 12) or between pairs of tissues (green; *n* = 110) as a function of threshold association score. Scores only include protein pairs quantified for both tissues or replicates. Shown is the median probability across pairs of replicates or tissues. The shaded area shows the interquartile range. **f**, Likely associations shared between pairs of tissues as quantified by the Jaccard index (gray dots), compared to shared associations restricted to complex members (CORUM), physical associations (STRING scores > 400), biological pathways (Reactome) and signaling (SIGNOR) (purple dots) or associations detected through yeast two-hybrid (HuRI) or AP (BioPlex) experiments (blue dots). Each dot represents a pair of tissues. Error bars show the mean and s.e.m. (*n* = 55). Source data

With the replicate cohorts representing the tissue of origin, we aggregated the association probabilities from cohorts of the same tissue into single association scores for 11 human tissues (Fig. 2a and Methods). Aggregating the replicate cohorts was advantageous, as all but one of the individual cohorts were outperformed by the tissue-level scores for recovering known protein interactions (*P* = 1.3 × 10^−9^, according to a one-sided Welch’s *t*-test). Moreover, the tumor-derived scores outperformed the healthy-tissue-derived scores for all tissues (Fig. 2b; AUC = 0.87 ± 0.01 and 0.82 ± 0.01, respectively, *P* = 8.3 × 10^−5^, according to a one-sided Welch’s *t*-test). In addition to the biopsy, where the genetic heterogeneity of tumors increased variation between samples (Supplementary Fig. 8), we found several other factors affecting the recovery of known interactions, such as the available number of cohorts per tissue, the number of samples per cohort, the tissue of origin and the MS methodology (Supplementary Figs. 2 and 9). The healthy-tissue-derived and tumor-derived scores originated from separate dissections of the same tissues and participants and could, thus, serve as independent replicates. Analogous to the cohorts, we computed tissue-specific associations from the healthy-tissue-derived scores, which we then recovered with the tumor-derived scores (Fig. 2c). For all tissues, we found that the tumor-derived scores primarily recovered the tissue-specific associations of the same healthy tissue (AUC = 0.74 ± 0.02) compared to the other healthy tissues (AUC = 0.53 ± 0.01, *P* = 5.9 × 10^−5^, according to a one-sided Welch’s *t*-test). These analyses show that the coabundance-derived tissue-level association scores recover known protein interactions and are reproducible and representative of the tissue of origin (Supplementary Fig. 10).

We defined a protein association atlas with association scores for all quantified protein pairs by averaging the association probabilities over the cohorts of each tissue. The resulting association atlas scores the association likelihood for 116 million protein pairs across 11 human tissues (Fig. 2d). On average, each tissue contains association scores for 56 ± 6.2 million protein pairs, of which 10 ± 1.0 million are likely to be associated (score > 0.5, average accuracy = 0.81 over all tissues, recall = 0.73 and diagnostic odds ratio = 13.0) and 0.49 ± 0.08 million are ‘confident’ associations (score > 0.8, average accuracy = 0.99 across tissues, recall = 0.21 and diagnostic odds ratio = 31.9) (Supplementary Fig. 11). These protein associations tended to be likely and confident in only a few tissues, with 99,103 protein pairs having likely associations in all tissues (Fig. 2d and Supplementary Fig. 12).

### Differences between tissues not driven by gene expression

One of the well-known drivers of differences in protein interactions between tissues is gene expression; proteins can interact only if their gene is expressed in a tissue. Indeed, the proteins that were quantified in a given tissue were generally enriched for genes with elevated expression for that same tissue but not the other tissues (Supplementary Fig. 13; *P* = 1.3 × 10^−6^, according to a one-sided Mann–Whitney *U*-test). However, only up to 7% of differences in (likely) associations between tissues can be explained by differences in gene expression and only through the lack of detection (Supplementary Fig. 14). These observations demonstrate that the likely associations for each tissue reflect but are not defined by differences in gene expression, further supporting our previous observation that protein coabundance is primarily driven by post-transcriptional processes.

Having established that association scores generally reproduce well and that differences between tissues are not driven by gene expression, we sought to measure the share of tissue-specific associations. To do so, we used a threshold association score to quantify the percentage of a tissue’s associations that were likely (score > 0.5) for the replicate (Fig. 2e, orange curve, comparing healthy-tissue-derived and tumor-derived replicates). As expected, we found that the percentage of likely associations increases with the threshold score, with 46.3% of likely associations and 90.2% of confident associations (score > 0.8) also being likely for the replicate tissue. Similarly, we found that these percentages decreased to 32.9% and 54.6%, respectively, when comparing associations between pairs of tissues from the association atlas (Fig. 2e, green curve). Lastly, depending on threshold scores, we found between 18.8% and 34.0% (interquartile range) of likely associations to be tissue specific between pairs of tissues, given the difference in probabilities between the curves for replicates and tissues. Therefore, with up to 7% of likely associations not quantified in other tissues because of gene expression (Supplementary Fig. 14), we estimated over 25.8% (18.8% + 7%) of likely associations to be tissue specific.

### Tissues recover tissue-specific cellular components

We sought to characterize the likely associations that were shared between tissues. Compared to all likely associations (average Jaccard index = 0.19), we found that the similarity between pairs of tissues increased as we restricted the likely associations to interactions identified through high-throughput screens such as yeast two-hybrid (Jaccard index = 0.30; HuRI)^3^ or AP (Jaccard index = 0.41; BioPlex)^4^ experiments (Fig. 2f). Likewise, the similarity between pairs of tissues increased when restricting likely associations to known interactions reported for signaling (Jaccard index = 0.32; SIGNOR)^79^, biological pathways (Jaccard index = 0.48; Reactome)^80^, physical associations (Jaccard index = 0.56; STRING)^81^ or human protein complexes (Jaccard index = 0.74; CORUM)^76^. Lastly, we found that the quantified differences and similarities between tissues were not sensitive to the choice of score cutoffs (Supplementary Fig. 15). Thus, known protein interactions are typically shared by the tissues in our association atlas, with signaling interactions being less commonly recovered between tissues than stable protein complexes. These observations reflect the divergence between tissues for different types of interactions and may also reflect differences in accuracy for recovering associations for stable protein complexes compared to spatiotemporal interactions that are dynamic.

Well-characterized protein complexes were generally preserved across tissues, becoming more variable as the complex-averaged association scores decreased (*ρ* = −0.77, *P* = 6.2 × 10^−125^) (Supplementary Fig. 16). As seen in other proteomics datasets^82^, more variable complexes are typically involved signaling and regulation (for example, tumor necrosis factor and emerin), while more stable complexes are involved central cellular structures (for example, ribosomes and the respiratory chain) (Supplementary Fig. 16). While protein complexes varied little between tissues, we found that associations varied strongly for tissue-specific cellular components, for example, for the brain (synapse-related components), throat (structural components of muscle fiber), lung (motile cilia) and liver (peroxisomes) (Supplementary Fig. 17). This suggests that tissue-specific and cell-type-specific cellular components are an important driver of tissue-specific protein associations that are independent of simple expression differences.

### Association atlas reveals cell-type-specific associations

**Fig. 3:**
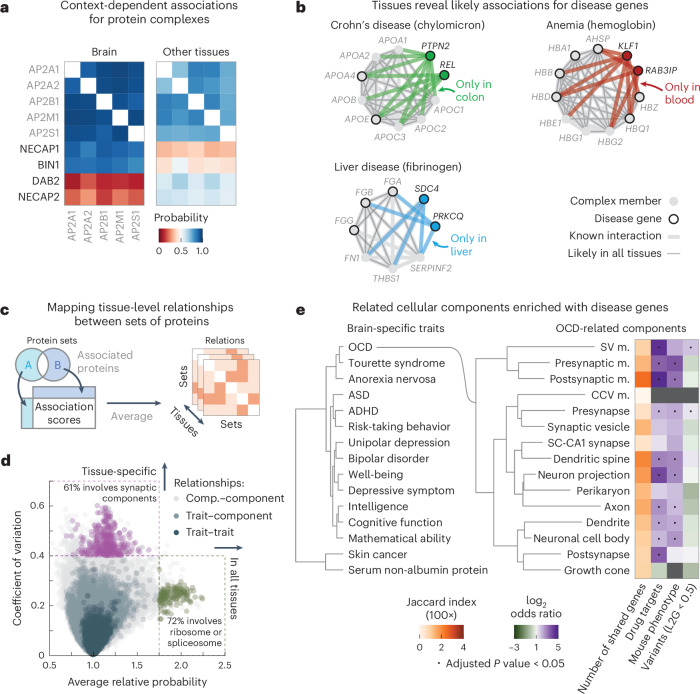
Association scores define relationships between protein sets. **a**, Association scores between AP2 subunits and known AP2 interactors (STRING scores > 400) that are synaptic proteins (NECAP1 and BIN1; SynGO) or not (DAB2 and NECAP2). Heat maps show association scores in the brain and averaged association scores for the other tissues. **b**, Associations of hemoglobin (GO:0005833) to anemia, chylomicron (GO:0042627) to Crohn’s disease and fibrinogen (GO:0005577) to liver disease. Proteins (nodes) are complex members (gray) and disease genes (black edge). Associations are likely in all tissues (thin gray lines) or likely in a single tissue and not likely in all others (thick colored lines). Thick gray lines are associations with prior evidence (STRING scores > 400). Disease genes defined through OTAR (Methods). **c**, Schematic of approach. Relationships are scored by aggregating the association scores between all pairs of proteins from disjoint sets. **d**, Relationship scores of cellular components (light gray; GO), GWAS traits (dark blue; OTAR L2G ≥ 0.5) and between traits and components (light blue). Each dot represents the relationship between two sets, indicating the average and CV of relationship scores relative to the tissue median. Green dots show relations of the ribosome and spliceosome (score > 1.75; green box); purple dots show relations of synaptic components (CV > 0.4; purple box). Comp., component. **e**, Dendrograms of the 15 most brain-specific GWAS traits (left) and the 15 GO cellular components having the most brain-specific relationship with OCD (L2G ≥ 0.5; right). Dendrograms were constructed with complete-linkage clustering using the Manhattan distance on the relationship scores between traits (left) or between cellular components (right). Heat maps show genes overlapping between cellular components and OCD (orange; Jaccard index) or the enrichment of nonoverlapping genes from cellular components with drug targets, genes associated with OCD in mice or genes less confidently linked to OCD through GWAS (purple–green; conditional log_2_ odds ratios of one-sided Fisher exact test; dots show BH-adjusted *P* values < 0.05; Methods). SV, synaptic vesicle; CCV, clathrin-coated vesicle; m., membrane; SC, Schaffer collateral; ASD, autism spectrum disorder. Source data

To explore cell-type-specific associations in our association atlas, we took the AP2 adaptor complex as a well-known example. The AP2 complex has neuron-specific functions in addition to functions that are general to all cells^83^. Indeed, the subunits of the AP2 complex were coabundant in all tissues (average association score between subunits = 0.80). We found 91 proteins that had association scores with all AP2 subunits in all tissues and were known to associate with AP2 (STRING score > 400). Among these, the 51 synaptic proteins (SynGO^84^) had higher association scores with the AP2 complex in the brain (average score = 0.54) compared to the other tissues (average score = 0.48 ± 0.00, *P* = 6.7 × 10^−6^, according to a one-sided Mann–Whitney *U*-test). Conversely, the nonsynaptic interactors had lower association scores with the AP2 complex in the brain (average score = 0.33) compared to the other tissues (average score = 0.43 ± 0.00, *P* = 1.1 × 10^−^^21^, according to a one-sided Mann–Whitney *U*-test) (Fig. 3a). We explored further examples by focusing on cell-type-specific associations in the context of disease. We found that proteins of hemoglobin are related to anemia and have likely associations with anemia proteins but only in the blood (Fig. 3b and Methods). Likewise, we found that subunits of chylomicron, which transports dairy lipids from the intestines, contain and have likely associations with proteins related to Crohn’s disease but only in the colon^85,86^. Lastly, we found that subunits of fibrinogen, synthesized in the liver, contain and have liver-only likely associations with proteins related to liver disease^87,88^. For the other tissues, we could find many examples of tissue-specific and cell-type-specific associations for protein complexes, cellular components and disorders such as diabetes and asthma (Supplementary Fig. 18). These examples demonstrate that our association atlas can be used to study tissue-specific functions of protein complexes and context-dependent associations for disease genes.

### Tissue-specific relations of traits and cellular components

We sought to generalize these examples of context-specific associations by systematically mapping the relationships amongst traits and multiprotein structures such as complexes or cellular components. As sets of proteins, we defined cellular components by Gene Ontology (GO) and defined human traits on the basis of the genome-wide association studies (GWAS) Open Targets (OTAR) locus-to-gene (L2G) score (≥0.5)^89–91^. We then scored the relationship between sets of proteins with the median association score of all possible protein pairs between the sets (Fig. 3c, schematic, omitting the intersection between gene sets). In total, we scored the relationships between traits (107,306 pairs), between traits and cellular components (240,967 pairs) and between components (134,002 pairs) across all tissues (Fig. 3d and Supplementary Tables 2–4). The relationship scores that were high in all tissues were primarily of core cellular components such as the ribosome and spliceosome (72% of relationships with relative average score > 1.75), while the relationship scores that varied most across tissues often involved tissue-specific structures such as synaptic components (61% of relationships with a coefficient of variation (CV) > 0.4). These observations suggest that the relationship scores recapitulate the relatedness of protein sets in a tissue-specific manner, particularly for the brain.

### Relationship scores for prioritizing disease genes

Additionally, we unbiasedly scored the tissue specificity of each protein set as the median association score between all pairs of its proteins (Methods and Supplementary Tables 5 and 6). Using these scores, we then selected the 15 traits most specific to the brain, 13 of which were indeed related to the brain (Supplementary Table 7). Clustering these traits using the trait–trait relationship scores from the brain revealed a hierarchical organization of traits with co-occurring conditions such as anorexia nervosa, obsessive–compulsive disorder (OCD) and Tourette syndrome closely clustering together^92,93^ (Fig. 3e, left dendrogram). As an example, we further determined the 15 cellular components that had the strongest brain-specific relationships with OCD, all but one of which were related or specific to neurons (Fig. 3e, right dendrogram, and Methods). The majority of these cellular components had few genes in common with the genes confidently associated with OCD (Fig. 3e, orange heat map; Jaccard indices < 0.04). However, after removing the few genes confidently associated with OCD through GWAS, we found that almost all components were still enriched with or contained OCD-related genes (Fig. 3e, purple–green heat maps), that is, drug targets for OCD (odds ratio = 8.4 ± 1.8; ChEMBL clinical stage 2 or higher)^94^, genes related to OCD from mouse deletion phenotypes (odds ratio = 4.8 ± 0.8; International Mouse Phenotyping Consortium (IMPC) score ≥ 0.5)^95^ or genes less confidently linked to OCD through GWAS (odds ratio = 1.6 ± 0.2, OTAR L2G score < 0.5) (Methods). Moreover, these 15 components with the strongest OCD relationships in the brain were more strongly enriched with OCD-related genes than other cellular components that contained OCD-linked genes (*P* = 4.3 × 10^−11^ (drug targets), 3.4 × 10^−^^15^ (genes related to OCD in mice) and 6.6 × 10^−^^7^ (genes less confidently linked to OCD through GWAS), according to one-sided Mann–Whitney *U*-tests).

Together, the results above demonstrate that the proposed relationship scores can prioritize cellular components that are enriched with trait-relevant genes. Analogously, we found that we could use the relationship scores for reconstructing the hierarchical organization of the cell, including maps of subcellular structures and modules of tissue-specific relations between cellular components (Supplementary Fig. 19). These observations demonstrate the potential for our association atlas to facilitate the systematic mapping of relations among traits, cellular compartments and likely other ontology terms.

### Validated brain interactions for schizophrenia-related genes

The results above indicate that the tissue-specific associations could facilitate the prioritization of disease-linked genes by functional association. Indeed, direct interactors of disease-linked genes have been used to prioritize causal genes in genetically linked loci and shown to be enriched in successful drug candidates^96–98^. To explore this in more detail, we constructed a network of brain interactions for schizophrenia (SCZ)-related genes. Specifically, we sought to prioritize highly ranked associations for the brain that involve SCZ-related genes and that have additional evidence from orthogonal methodologies.

**Fig. 4:**
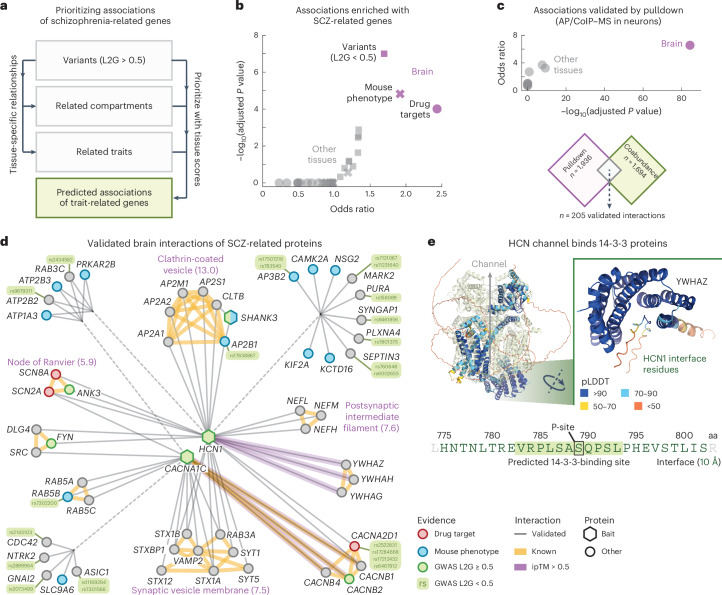
Network of validated brain associations for SCZ-related proteins. **a**, Schematic of approach. Genetic variants associated with SCZ are used together with relationships between traits and cellular components and the tissue scores to prioritize associations for SCZ-related genes. **b**, Enrichment of predicted associations with genes related to SCZ through mouse phenotypes (IPMC; crosses), drug targets (clinical stage 2 or higher; circles) or GWAS variants (L2G scores < 0.5; squares). **c**, Enrichment of pulldown interactions in the predicted associations for SCZ-related genes. In **b**,**c**, purple symbols represent the brain and gray symbols represent other tissues. Scatter plots show the conditional odds ratios and BH-adjusted *P* values, according to one-sided Fisher exact tests. **d**, Simplified network of validated brain interactions for SCZ-related genes (Methods). Circular and hexagonal nodes were prey and bait proteins in the pulldown studies, respectively. Nodes are colored as GWAS variants (green), drug targets (red), associated with SCZ in mice (blue) or other (gray). Gray edges were predicted from association scores in the brain and validated through pulldown experiments. Yellow edges are known interactors (physical associations in STRING; scores > 750). Purple edges have ipTM scores > 0.5. Purple labels annotate subgraphs of known interactors with the most enriched GO cellular components (exponent of BH-adjusted *P* value between brackets, according to a one-sided Fisher exact test). **e**, AlphaFold2 model of the interface between HCN1 and 14-3-3 proteins. Shown are the HCN complex (Protein Data Bank 6UQF; light green) aligned with the AlphaFold2 model of HCN and YWHAZ. The sequence shows the 14-3-3-binding site of HCN1 according to the AlphaFold2 models (green text, 10-Å cutoff; inset), overlaid with the predicted 14-3-3-binding site (green box, 14-3-3-Pred score = 0.457) and phosphorylation site S789 (black box). Interface residues are colored by predicted local-distance difference test. Source data

We started by taking *n* = 369 genes associated with SCZ through GWAS studies (‘starting genes’, L2G scores ≥ 0.5) and computed the top 25 traits and cellular components that had the strongest tissue-specific relation to SCZ in each tissue (Fig. 4a and Methods). This gave us a collection of genes related to SCZ in a tissue-specific manner. For each tissue, we then filtered for protein pairs that had one SCZ starting gene and one SCZ-related gene and required these protein pairs to have association scores exceeding the 97th percentile of the tissue scores (association score = 0.70 ± 0.01, 0.73 in the brain), leading to tissue-specific networks of associations for SCZ-related genes (Supplementary Fig. 20 and Supplementary Table 8). After removing the SCZ starting genes from the brain network, the remaining genes were still enriched for genes associated with SCZ in mice (Benjamini–Hochberg (BH)-adjusted *P* value = 1.5 × 10^−^^5^, IMPC score ≥ 0.5), drug targets for SCZ (BH-adjusted *P* value = 9.8 × 10^−^^5^, ChEMBL clinical stage 2 or higher) and other variants associated with SCZ (BH-adjusted *P* value = 1.0 × 10^−^^7^, OTAR L2G scores < 0.5), according to one-sided Fisher exact tests. This enrichment was specific to the brain compared to any of the other tissues, suggesting that the proposed methodology presents a systematic approach for prioritizing disease genes of tissue-specific traits (Fig. 4b).

Indeed, for brain-related disorders, including autism, attention deficit hyperactivity disorder (ADHD), Tourette syndrome and others, we found that the proposed methodology of prioritizing protein associations for disease-linked genes enriched, specifically in the brain, for other genes associated with the respective disorder through mouse phenotypes or drug targets, with many also being enriched for GWAS variants that are less confidently linked to the disorder (Supplementary Fig. 21).

Having established that the presented methodology prioritizes protein associations that enrich for disease-related genes in a tissue-specific manner, we sought to validate the prioritized protein associations through additional evidence. Specifically, to further validate the networks of predicted associations for SCZ-related genes, we assembled a curated dataset of brain interactions established experimentally through pulldowns using human brain cells (that is, AP–MS or coimmunoprecipitation–MS in human microdissected brain tissue or human induced pluripotent stem cell-derived neurons^99–103^). This dataset contained 7,887 human brain interactions for 30 bait proteins and has been incorporated into the IntAct database^9^ (Supplementary Table 9). We filtered these brain interactions for the bait proteins that were associated with SCZ (OTAR L2G score ≥ 0.5) and further filtered the tissue-specific networks of SCZ-related genes for associations involving at least one bait protein. The remaining associations of SCZ-related genes were strongly enriched with the interactions from pulldowns with SCZ-related baits, especially for the brain (log BH-adjusted *P* value = 84.3, according to a one-sided Fisher exact test) compared to the other tissues (log BH-adjusted *P* value = 1.8 on average) (Fig. 4c). Thus, the brain associations of SCZ-related genes but not the other tissues were experimentally validated by pulldown studies and enriched with SCZ-related association partners.

Next, we filtered the prioritized brain associations for SCZ-related genes for interactions that were also found through the pulldown studies to obtain a network of 205 validated brain interactions for SCZ-related genes (average association scores = 0.86 in the brain), which we simplified to only show synaptic (SynGO) genes having prior evidence (Fig. 4d, Methods and Supplementary Table 10). The visualized network contained 56 proteins connected to three bait proteins through a collection of 66 validated brain interactions. These connected proteins included SCZ drug targets (3 proteins; clinical stage 2 or higher), proteins associated with SCZ in mice (12 proteins; IMPC score ≥ 0.5) or proteins linked with SCZ with weaker prior evidence (15 proteins; OTAR L2G scores < 0.5). Surprisingly, only four of the visualized brain interactions were confidently reported in any of the major protein interaction databases before our curation effort (CACNA1C with CACNB1, CACNB2 and CACNB4 (STRING scores > 750) and SRC (Signor)) and only the interactions of SHANK3 with AP2B1 and CLTB were quantified in more than three other tissues (association scores = 0.34 ± 0.08 and 0.29 ± 0.04 in the other tissues). These observations support our earlier analysis that the network of validated interactions of SCZ-related genes is specific to the brain.

The visualized network contained several groups of highly interconnected proteins (STRING scores > 750). These groups were enriched with genes of cellular components typical for neuronal functioning and SCZ, such as a group of proteins for the postsynaptic cytoskeleton^104^ (BH-adjusted *P* value = 2.3 × 10^−^^8^) or for clathrin-coated vesicles (9.4 × 10^−^^14^), according to one-sided Fisher exact tests. For the clathrin vesicle coat, the network connected all subunits of the AP2 complex and clathrin proteins to HCN1. Interestingly, previous pulldown studies showed that HCN channels directly interact with TRIP8b (refs. ^105,106^). TRIP8b regulates the trafficking of HCN channels^106^ and mainly associates with the AP2 complex^107^. Moreover, while AP2 and clathrin are not cell type specific, both HCN1 and TRIP8b were found to be enriched at parvalbumin (PV)-positive synapses^108^, with HCN channels being specific to PV neurons and important for their high firing frequencies^109,110^. Given the link of PV neurons with SCZ^111–115^, these observations suggest that AP2 and clathrin may be involved in a PV neuron-specific disruption of HCN channel trafficking with SCZ.

To suggest putative interface models for the validated brain interactions for SCZ-related genes, we used AlphaFold2 to predict the structures for 205 protein interactions, including the entire visualized network (Fig. 4d and Supplementary Table 11). The predicted models had more confident interactions compared to models for known complex members from CORUM^116^ (average pDockQ scores = 0.20 and 0.13, respectively, *P* = 1.6 × 10^−^^19^, according to a one-sided Mann–Whitney *U*-test). In total, we identified 15 moderate-confidence interactions (interface predicted template modeling (ipTM) scores > 0.5). These included the brain-specific binding of all three 14-3-3 proteins (YWHAG, YWHAH and YWHAZ) with HCN1 (Fig. 4e; average association score = 0.82, average ipTM score = 0.65). The interfaces of these three models overlap and are located in the C-terminal disordered region of HCN1 (residues 775–802). This consensus interface includes a predicted 14-4-3-binding site (centered around S789; average ipTM score = 0.75 at the putative binding site, 14-3-3-Pred score = 0.457)^117^ that has been verified through pulldown experiments^118^. Indeed, the binding of 14-3-3 proteins with HCN1 was found to be dependent on the phosphorylation of S789, with the interaction between 14-3-3 and HCN1 likely inhibiting HCN1 degradation^118^.

Lastly, the network contained 15 genes within loci genetically associated with SCZ that had weaker evidence supporting them as the causal genes at each locus. Given their interaction with other SCZ-related genes, these could be prioritized as more likely causal because of their functional roles. Some of these genes (*AP2B1*, *ATP2B2* and *SYNGAP1*) had the highest L2G score for their respective locus with single-nucleotide polymorphisms (SNPs) linked to SCZ but had scores below the 0.5 cutoff used (0.457, 0.264 and 0.251, respectively)^90,91^. In addition to the AP2 complex, we found a member of the AP3 complex (*AP3B2*). *AP3B2* had the second highest score for the locus with SNPs (rs783540), which had splicing and expression quantitative trait locus (QTL) associations with *AP3B2* but was ranked higher for disruption of *CPEB1* given that the variant lies within a *CPEB1* intron. Similarly, *MARK2* was ranked second for two SNPs (rs7121067 and rs11231640), both having splicing and promoter capture Hi-C associations with *MARK2* but being ranked higher for disrupting *RCOR2* because of proximity to its transcription start site. *CDC42* and *NTRK2* had the second highest L2G scores for their locus but the top associated genes (*WNT4* and *AGTPBP1*, respectively) had lower and less specific expression in the brain^12^.

### Cofractionation-derived synapse-specific interactome

**Fig. 5:**
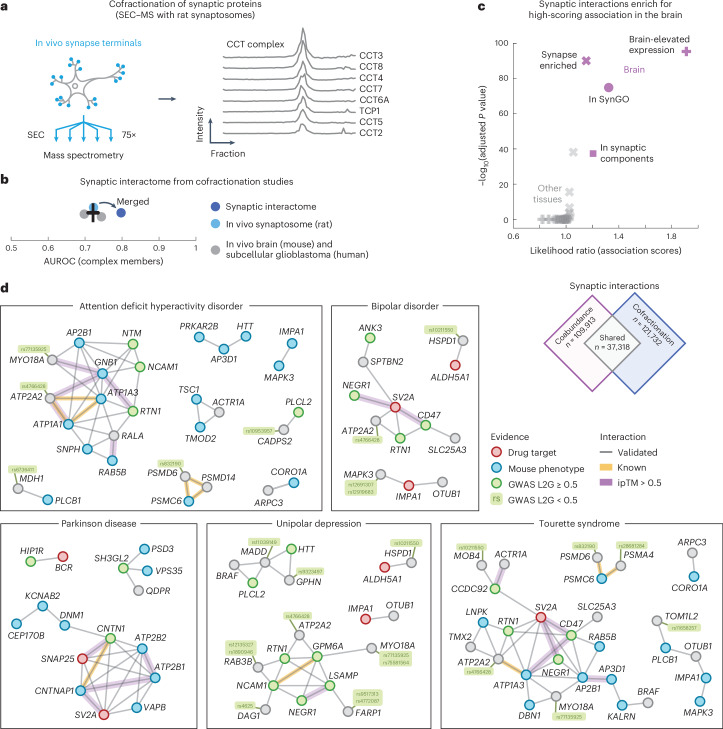
Network of cofractionation-derived synaptic interactions. **a**, Schematic of experiment. In vivo synaptosomes (light blue) from rat neurons were purified and fractionated into 75 fractions through SEC and subjected to LC–MS/MS. Elution profiles show the protein intensities from MS for the CCT complex members (right). **b**, AUC values for the cofractionation studies (in vivo mouse brain, gray; subcellular glioblastoma, gray; in vivo rat synaptosome, light blue) and for the merged synaptic interactome (dark blue). Positives were defined by complex members in CORUM. The error bar shows the mean and s.e.m. (*n* = 3). **c**, Comparison of association scores of interactions between synaptic proteins or interactions of other proteins in the brain (purple) and the other tissues (gray). Interactions are from the synaptic interactome (score > 0.8). Data were derived using a one-sided Mann–Whitney *U*-test, BH-adjusted *P* values and median likelihood ratios. Synaptic proteins included those that were enriched in the synapse (crosses), reported in SynGO (circles), associated with GO synaptic components (squares) or had brain-elevated expression according to The Protein Atlas (pluses). **d**, Networks of validated interactions between synaptic proteins (SynGO or enriched in mouse tissues) related to ADHD, bipolar disorder, Parkinson disease, unipolar depression or Tourette syndrome. Nodes are colored by association to the trait through GWAS (green), drug targets (red), mouse phenotypes (blue) or other (gray) (Methods). Gray edges were predicted from association scores in the brain and validated through the cofractionation studies. Yellow edges are known interactors. Purple edges have ipTM scores > 0.5. Source data

As a final application of our protein association atlas, we focused on the interactome of synapses. To do so, we prepared and purified synaptosomes from rat brains as an orthogonal approach for validating the brain associations. We fractionated the synaptosomes with size-exclusion chromatography (SEC) into 75 fractions that were then subjected to liquid chromatography (LC)–MS/MS. A total of 3,409 unique proteins were detected, including well-known protein complexes such as the CCT complex subunits, whose profiles correlate across the fractions (Fig. 5a; average correlation coefficient = 0.96).

We preprocessed the fractionation profiles of the synaptosome and computed the coabundance of proteins across fractions to score the cofractionation of 4,276,350 protein pairs in rat synapses (Methods). To increase the confidence of the interaction scores, we then combined the rat synaptosome with other cofractionation profiles from in vivo mouse brains^6^ and subcellular fractionation profiles from human glioblastoma cells^7^. We merged the cofractionation studies by orthologs that were quantified in all three datasets and computed interaction probabilities with a logistic model using the CORUM database as positives (Methods and Supplementary Table 12). The resulting synaptic interactome quantified 1,309,771 interaction probabilities for 1,619 proteins and improved the recovery of known interactions compared to the cofractionation studies individually (Fig. 5b; AUC = 0.80 and 0.72). Of the 1,619 proteins in the interactome, 24% are annotated as synaptic proteins in the SynGO database, 49% have been reported as synapse-enriched in mouse brains and 56% have previously been identified through crosslinking MS (XL-MS) of the mouse synaptosome^84,108,119^. All XL-MS interactions for these proteins were quantified in our synaptic interactome, having association scores of 0.59 compared to 0.37 for other associations (*P* = 2.0 × 10^−^^44^, according to a one-sided Mann–Whitney *U*-test). Moreover, the synaptic interactions between synapse-enriched proteins were more likely compared to other interactions (average association scores = 0.40 and 0.36, *P* < 1 × 10^−^^300^, according to a one-sided Mann–Whitney *U*-test). Together, these observations suggest that our synaptic interactome largely aligns with current state-of-the-art synaptic interaction resources and scores the likelihood of interactions for a wide range of synaptic proteins, with interactions being more likely for synaptic proteins compared to other proteins.

### Validated synaptic interactions for brain disease genes

The interactions between synapse-enriched proteins formed more likely associations compared to associations of nonsynaptic proteins, especially for the brain compared to the other tissues of our association atlas (Fig. 5c). Given this brain-specific elevated likelihood of synaptic interactions, we constructed a network of interactions between synaptic proteins (in SynGO or synapse-enriched in mouse brains) that were both likely coabundant in the brain (109,913 associations) and likely cofractionated for the synaptosome (121,732 interactions). The resulting network consisted of a collection of 37,318 validated protein interactions between synaptic proteins (Supplementary Table 13). These synaptic interactions were primarily specific to the brain because few of the interactions were likely for the majority of other tissues in our association atlas (20%) and only a small fraction have been reported in any protein interaction database (5.8% in STRING, 1.3% in HuMAP, 3.6% in IntAct and 1.6% in BioPlex).

We were particularly interested in the validated interactions of synaptic proteins that are associated with brain disorders. As before, we filtered for GWAS traits that had associated genes through mouse phenotyping (IMPC) or known drug targets (ChEMBL) and whose trait-level association scores were elevated in the brain compared to the other tissues (*z* score > 1; Methods). We found that 10 of the resulting 13 traits were disorders clearly related to the brain and selected the 727 confident synaptic interactions between genes associated with these brain-specific traits (association scores = 0.7 and 0.81 on average in the synaptosome and brain, respectively). Additionally, to suggest putative interface models, we used AlphaFold2 to predict the structures for these 727 interactions. The predicted models had more confident interactions compared to models for known interactors in CORUM (average pDockQ scores = 0.28 and 0.13, *P* = 3.7 × 10^−^^147^, according to a one-sided Mann–Whitney *U*-test) or HuMAP (pDockQ scores = 0.28 and 0.25, *P* = 2.4 × 10^−^^17^) and high-confidence models (ipTM scores > 0.7) were enriched for additional evidence from XL-MS experiments in mouse synaptosomes^119^ (Supplementary Fig. 22). In total, we identified 105 moderate-confidence interactions (ipTM scores > 0.5; Supplementary Table 14). Lastly, we visualized (simplified) the networks of validated synaptic interactions between trait-related genes of the brain disorders (Fig. 5d and Methods).

### Prioritizing synaptic disease genes for brain disorders

Several genes in the networks had weaker prior evidence supporting them as the causal genes at loci genetically associated with the brain disorders. These genes could be prioritized as more likely to be causal for the disorders because of their validated synaptic interactions with other genes that were confidently associated with the same disorders. As before, we looked for genes with the highest (below cutoff) L2G scores for their respective loci with SNPs linked to the brain disorders. We found likely causal genes for ADHD (*MDH1*, score = 0.491; *CADPS2*, score = 0.340; *PIK3C3*, score = 0.323), SCZ (*TOM1L2*, score = 0.492; *AP2B1*, score = 0.457; *PSD3*, score = 0.429; *MYO18A*, score = 0.428; *ATP2B2*, score = 0.264; *TMX2*, score = 0.232), Alzheimer disease (*CLPTM1*, score = 0.378; *MADD*, score = 0.315), autism (*ATP2B2*, score = 0.264; *ATP2A2*, score = 0.243), unipolar depression (*MADD*, score = 0.244) and bipolar disorder (*ATP2A2*, score = 0.206), with the last variant (*MYO18A*, score = 0.428) additionally being likely causal for Tourette syndrome, OCD, ADHD and unipolar depression^90,91^. Of these genes, all but *ATP2A2*, *MDH1*, *MYO18A* and *PIK3C3* had the highest or second highest expression in brain tissue^12^.

Additionally, we found genes with synaptic interactions that did not have the highest L2G score for the respective SNP linked to the brain disorders but still had additional evidence. For example, we found that *PAFAH1B1* associated with *RHOA* in our synaptic interactome, with both having weak prior evidence for being causal to depression. *PAFAH1B1* has a role in neural mobility and is required for activation of Rho guanosine triphosphatases such as RhoA^120^. *PAFAH1B1* was the second highest scoring gene for the locus with an SNP (rs12938775) linked to unipolar depression. This variant has expression QTL associations with *PAFAH1B1* and lies within a *PAFAH1B1* intron, despite being more distant to the transcription start site of *PAFAH1B1* compared to the highest scoring gene (*CLUH*). However, *PAFAH1B1* encodes a synaptic protein, whereas *CLUH* does not, with the expression of *PAFAH1B1* being higher and more specific to the brain compared to *CLUH*^12^. Overall, this example demonstrates how orthogonal approaches targeting subcellular structures and individual tissues can provide tissue-specific protein interaction networks and aid in the prioritization of genes likely to be causal for tissue-related disorders.

## Discussion

Despite progress in mapping protein interactions on a large scale, we lack a comprehensive understanding of how these interactions differ across tissues. It has been previously shown that the protein–protein associations derived from correlated protein abundance measurements can be more accurate than those derived from mRNA coexpression^15–18^. Here, we showed additionally that these associations can be more accurate than those found by correlation analysis of cofractionation. However, in line with other reports, we observed that the combination of different cofractionation datasets can improve the predictions^121^ and it is likely that more advanced analyses of cofractionation data may yield more accurate results^122^. Interestingly, we noted that we can exclude the variation in protein levels that is explained by mRNA changes without significantly affecting the performance of the method. This suggests that protein interaction partners are often post-transcriptionally coregulated. This is in line with the observations that protein abundances of subunits within a complex can be limited by the abundance of the complex itself, with active degradation of excess unbound subunits^15,16,22,24,123^.

Several lines of evidence attest to the quality of the derived association scores. Cohorts of the same tissue had similar association scores, association scores derived from tumor and healthy dissections of the same tissue tended to replicate in a tissue-specific manner and essential protein complexes were generally very well predicted across all tissues. Around 46% of likely associations and 90% of confident associations were replicated between healthy-tissue-derived and tumor-derived scores, suggesting that these associations, complemented with other data, can be useful in generating testable hypotheses regarding tissue-specific biology. In particular, our human tissue-derived associations can provide supporting evidence for interactions in human tissues obtained from less physiologically relevant material, given that human tissue is often not available for interaction studies. Taking the brain as example, we complemented the coabundance associations with experimental data from pulldowns, cofractionation and AlphaFold2 predictions to provide a more confident subset of interactions for disease–gene mapping.

We estimated that over 25% of associations are tissue specific. While we observed a large fraction of associations that were not quantified in different tissues, likely because of technical issues of detectability, we estimated that only a small fraction of the differences across tissues are because of changes in expression levels (up to 7%). We found that cell-type-specific structures such as synaptic components may contribute greatly to differences in protein associations across tissues, with differences in post-translational modifications being other mechanisms that could explain some variation in tissue-specific protein associations.

Lastly, we showed that our atlas can be used to derive relations between traits and with cellular components. This approach is particularly effective for disorders of the brain and synaptic components, demonstrating that the network of brain associations is more specific and distinct from the other tissues. We presented validated brain interactions enriched for disease-associated genes and drug targets and showed how this can be useful to prioritize novel disease-linked genes and presumably drug candidates. The combination of genetics, tissue-specific networks and AlphaFold2 models provides an integrated network for enhanced understanding of disease mechanisms and target prioritization. In addition, our approach may improve the safety of targeting disease genes as the predicted disease–gene associations will tend to be tissue specific and, therefore, the proposed genes may be safer to target.

## Methods

### Preprocessing abundance data

Publicly available protein abundance data were filtered by removing nonabundance samples and measurements (internal controls, reference samples, samples that were withdrawn or that did not have further annotation, etc.). Protein measurements without gene identifiers were removed and the abundance data of cohorts with replicate samples for some but not all participants were averaged at the participant level (for 3 of 50 cohorts, for example, for cohorts having a sample with five technical replicates as the internal control). Zeros were replaced with NaN values for all entries of cohorts that were not log-transformed to ensure that undetected proteins did not have abundance data. Cohorts were then split into tumor and healthy samples if not reported separately. The sample names were homogenized to obtain paired RNA expression and protein abundance data and paired tumor and healthy biopsies. The gene identifiers were homogenized and converted to HUGO Gene Nomenclature Committee (HGNC) gene symbols across cohorts to allow for comparison between studies (Supplementary Table 15 and Supplementary Methods). Then, sequentially, duplicate measurements were averaged (that is, averaging the reported abundance of proteins whose identifier occurred more than once), the protein abundances were median-normalized for each participant, the data were log_2_-transformed if not already on a log scale (13 of 50 cohorts) and proteins with measurements in fewer than ten participants were removed. Note that abundances were not further normalized across participants because correlation coefficients are invariant under linear transformations. RNA expression data were processed in identical fashion. As deviations from the above, samples were removed from cohorts that shared participants by filtering the follow-up studies for participants whose protein abundance was not previously reported^47,48^.

### Computing association probabilities

Coabundance estimates were computed by calculating the correlation matrix consisting of Pearson correlations between all possible pairs of proteins for a cohort. Each pair of proteins was required to have at least *n* = 30 paired abundances across samples for computing a correlation value (that is, protein pairs had to be quantified in at least 30 of the same samples, available for 48 of 50 studies). Correlations were then reported for each unique protein pair (alphabetically and sorted by gene symbols), ignoring selfcorrelations and missing values. For each study, a logistic model was fitted to convert the correlations to association probabilities. The set of ground-truth positives was defined as all protein interactions reported in the CORUM database^76^. Specifically, all possible protein pairs from the subunits for each protein complex in CORUM were defined as ground-truth positives and all protein pairs that had correlation coefficients but were not ground-truth positives were labeled as negatives, resulting in a collection of labels ${\left\{\,{y}_{i}\right\}}_{i\in [1,n]}$ for the $n$ protein pairs (where $y=1$ is a positive and $y=0$ is a negative). Using the set of defined positives and negatives, a logistic regression with two free parameters was fitted for each study to the correlation values ${\left\{{x}_{i}\right\}}_{i\in [1,n]}$,

$${{\rm{logit}}\;p}(\,{y}_{i}|{x}_{i})={\beta }_{0}+{\beta }_{1} {x}_{i}.$$

Here, each study’s model had an intercept ${\beta }_{0}$, parameter ${\beta }_{1}$ and no penalty. The logit function was defined as ${{\rm{logit}}\; p}=\mathrm{ln}(\frac{p}{1-p})$ and the model’s parameters were optimized by minimizing the cost function,

$$\frac{1}{W}\mathop{\sum }\limits_{i=1}^{n}{w}_{i}\left(-{y}_{i} \log {p}\!\!\right.({x}_{i})-(1-{y}_{i}) \log \left(1-p({x}_{i})\right),$$

where $W={\sum }_{i=1}^{n}{w}_{i}$ is the total weight and ${\left\{{w}_{i}\right\}}_{i\in [1,n]}$ are the sample weights for the protein pairs. These sample weights were determined by balancing the positive and negative classes when fitting the logistic model. In detail, each sample was weighted inversely proportional to the number of samples in its class, such that the total weight of the positive samples was equal to the total weight of the negative samples. Specifically, with $Y={\sum }_{i=1}^{n}{y}_{i}$ being the total number of positives, the positive samples were weighted as ${w}_{i}=\frac{n}{2 Y}$ while the negative samples were weighted as ${w}_{i}=\frac{n}{2 (n-Y)}$, such that the total weight of the positives ($Y\times \frac{n}{2 Y}=\frac{n}{2}$) was equal to the total weight of the negatives ($(n-Y)\times \frac{n}{2 (n-Y)}=\frac{n}{2}$) and the weights in the cost function summed to 1,

$$\frac{1}{W}\mathop{\sum }\limits_{i=1}^{n}{w}_{i}=\frac{1}{W}\left(Y\times \frac{n}{2 Y}+(n-Y)\times \frac{n}{2 (n-Y)}\right)=1.$$

Additional details can be found in the implementation of the LogisticRegression function with the class_weight='balanced' option from the scikit-learn package. Finally, the fitted logistic model was used to transform the correlation values to probabilities for each study. Note that the logistic (log-linear) model yields a rank-preserving transformation, preserving the ranking of protein pairs when sorting correlations and probabilities from large to small. The association probabilities per tissue are available through the BioStudies repository for this paper (S-BSST1423).

### Computing interaction probabilities from cofractionation data

Cofractionation data were collected from studies of in vivo mouse tissues (SEC with stable isotope labeling in mammals; 2 × 55 fractions for seven tissues)^6^, human breast tissue-derived cell lines (ion exchange with high-performance (HP)LC–MS; 3 × 2 × 192 fractions of three cell lines with two technical replicates each)^77^ and subcellular fractionation of human cancer cell lines (high-resolution isoelectric focusing with LC–MS; 5 × 10 fractions for five different tissues and an additional 4 × 10 fractions from three replicates and one condition in the lung)^7^. The protein identifiers of the mouse data were converted to human orthologs (Ensembl BioMart, September 28, 2023). Replicates and conditions were concatenated and treated as different fractions. The resulting ten cofractionation datasets (seven mouse tissues, one human breast cell line, one human lung cell line and one human mixed cell line) were further processed as follows. Data were filtered for fractions and proteins that had nonzero measurements and abundances of duplicate protein identifiers were averaged. Fractions were then normalized by total intensity, missing values were imputed as 90% of the minimum nonzero value for each protein and the data were log_2_-transformed (mouse tissues and breast cell line). Setting empty fractions to 90% of the minimum nonzero value was advantageous as it increased the number of protein pairs having sufficient observations across fractions for computing association probabilities (3.6-fold more protein pairs on average) and improved the cofractionation estimates by including the fractions where proteins were coabsent (increased AUCs by 14% on average). Here, the cofractionation estimates were not sensitive to the value used for empty fractions (that is, 0.7% difference in AUC values on average between using 90% or 10% of the minimum nonzero values). Association probabilities were then computed as described earlier.

### Computing ROC curves and AUC values

We defined the set of ground-truth positives as all protein interactions reported in CORUM. Specifically, for each protein complex, the positives were defined as all possible pairs of subunits. The space of negatives was then defined as all protein pairs for which association scores were quantified for a given dataset (cohort, tissue, etc) but that were not ground-truth positives (in CORUM). ROC curves were computed as the true positive rates as function of the false positives rates across all possible score thresholds, using the defined positives and negatives as labels and the association probabilities as scores. AUCs for the ROC curves were computed using the roc_auc_score function from the scikit-learn package.

### Defining protein association atlas for human tissues

Association scores for a tissue were computed by averaging, for each protein pair, the association probabilities of all cohorts of the tissue. Missing values were ignored. Two cohorts were excluded on the basis of their performance for recovering tissue-specific associations of the other cohorts of their respective tissues (Supplementary Fig. 7). Tissues were only included if association probabilities from at least two studies were available (not for the bladder, prostate and uterus (tumor) and only for the lung, throat, liver, kidney, colon and stomach (healthy)). Likely associations were defined as the protein pairs whose association score exceeded 0.5 for a given tissue, whereas confident associations were defined as the protein pairs whose association score exceeded 0.8 for a given tissue.

### Proteins associated with human traits through GWAS variants, drug targets or mouse phenotypes

Evidence was downloaded from the OTAR repository (version 24.06)^90,91^. Three types of evidence collected from OTAR were used for associating genes to traits. Specifically, genes were considered associated with traits through GWAS, associated with traits through mouse phenotypes (IMPC)^95^ or as drug targets (ChEMBL)^94^. For genes associated with traits through GWAS, the evidence was loaded from the OTAR ‘ot_genetics_portal’ source (scores ≥ 0.5 (confident) or scores < 0.5 (weak)). For genes associated with traits through mouse phenotypes and drug targets, the evidence was loaded from the OTAR sources ‘impc’ (scores ≥ 0.5) and ‘chembl’ (clinical phase of at least 1 or at least 2), respectively. Gene identifiers were converted to HGNC gene symbols following the symbols used in the association atlas (Supplementary Table 15 and Supplementary Methods). Trait identifiers (experimental factor ontology) were converted to trait names using annotations from the OTAR platform (version 24.06). Evidence was then summarized per source (Supplementary Tables 16–19).

### Proteins associated with GO cellular components

The descriptions of GO terms were taken from their GO annotations and filtered for not being obsolete, having a description and being cellular components but not protein complexes (namespace = ‘cellular_component’, name does not contain ‘complex’; 2,068 components). The cellular components were annotated with associated genes using the QuickGO annotations from the European Bioinformatics Institute (EBI; August 27, 2024). Additionally, the cellular compartments were annotated with associated genes reported by UniProtKB (August 27, 2024) through their Rest application programming interface (API). Both sets of associated genes were merged and gene identifiers were converted to HGNC gene symbols following the symbols used in the association atlas (Supplementary Table 15 and Supplementary Methods). Lastly, 889 GO cellular components had associated genes (Supplementary Table 20).

### Trait-level and component-level association scores and relationship scores of traits and components

Protein sets were defined by the genes associated with GO cellular components (541 components having at least five associated proteins) or genes associated with traits through GWAS (465 traits having at least 20 associated proteins). Set-level association scores and relationship scores were computed as follows. Let ${S}_{A}$ be some protein set $A$. The set-level association score ${P}_{T}(A)$ for tissue $T$ and $A$ was defined as the median association score of all pairs of proteins in ${S}_{A}$ that were quantified for tissue $T$,

$${P}_{T}(A)={\rm{median}}{\left\{{P}_{T}(i,j)\right\}}_{i,j\in {S}_{A}}.$$

Analogously, the relationship score ${P}_{T}(A,B)$ for tissue $T$ and protein sets $A$ and $B$ was defined as the median of all association scores between proteins from $A$ (that were not in $B$) and proteins from $B$ (that were not in $A$),

$${P}_{T}(A,B)={\rm{median}}{\left\{{P}_{T}(i,j)\right\}}_{i\in {S}_{A}{\rm{\backslash }}{S}_{B},j\in {S}_{B}{\rm{\backslash }}{S}_{A}}.$$

Lastly, set-level association scores were computed for all protein sets in all tissues (Supplementary Tables 5 and 6), and relationship scores were computed between all pairs of protein sets in all tissues (Supplementary Tables 2–4). Relationship scores that could not be quantified were set as zero and ignored throughout.

### Structural models of protein–protein interaction interfaces

Structures were predicted using AlphaFold-multimer version 2.3.1. Input protein sequences were downloaded from UniProt (release 2023_03 to release 2024_02). The use of templates was turned on. Five structures were predicted for each protein pair and the predicted structure with the highest model confidence score was considered the ‘best’ structure for each pair. The pDockQ scores were calculated for the top-ranked structure using a previously published implementation^116^. Interaction interfaces were defined by considering that any residue with at least one atom within 10 Å (or 5 Å; Fig. 4e) of the other protein chain is part of the interface.

### Synaptosome preparation

Cortices of adult rats (Sprague–Dawley) were dissected and a synaptosome preparation was generated using Syn-PER (Thermo Fisher Scientific) according to the manufacturer’s instructions. All animal experiments were carried out under institutional guidelines (ZH172/18 Kanton Zürich Gesundheitsdirektion Veterinäramt).

### Cell lysis

The procedures used for cell lysis and SEC-based fractionation followed experimental protocols previously published^124,125^, with slight adaptations regarding the lysis, detergent and SEC column. In short, synaptosomes were lysed in HNN buffer (50 mM HEPES, 100 mM NaCl and 50 mM NaF) supplemented with protease inhibitor (1× of a 500× stock solution), 20 mM sodium vanadate (of a 200 mM stock solution), 1 mM PMSF (from a 100 mM stock solution) and 1% *n*-dodecyl β-d-maltoside (DDM), from a freshly prepared 10% DDM stock solution. Samples were lysed by gentle pipetting up and down. The lysate was preclarified by centrifugation at 16,000*g* at 4 °C for 20 min. The supernatant was split to multiple ultracentrifuge tubes to remove insoluble cell debris by ultracentrifugation in a TLA 100.1 rotor (Beckman-Coulter) at 35,000*g* at 4 °C for 15 min. Next, supernatants were transferred to Eppendorf tubes and protein concentration was assessed using the BCA assay (Thermo Fisher Scientific). A total protein amount of 1.2 mg (or 319 μl of protein lysate) was then diluted up to 4 ml with HNN buffer (without detergent). To concentrate the sample and remove surplus detergent, samples were concentrated over a filter with a 30-kDa molecular weight cutoff (MWCO; Amicon). Samples were centrifuged at 3,600*g* at 4 °C for 5 to 10 min with 5-min intervals to monitor lysate volume. First, samples were concentrated to approximately 500 μl, before diluting them again up to 1 ml. This dilution step was repeated a total of three times before samples were concentrated to approximately 100 μl.

### Separation of complexes by SEC

For the separation of complexes, a 1260 Infinity II HPLC system (Agilent) connected to an SRT-C SEC-1000 (Sepax) 7.8 × 300 mm column with an SRT-C SEC-1000 (Sepax) 7.8 × 50 mm precolumn was used. The system was operated at a flow rate of 500 μl min^−1^. The column was equilibrated with SEC buffer (50 mM HEPES and 100 mM NaCl, pH 7.5) for at least four column volumes. Before collection of the separated sample, a replicate of the rat synaptosome extract was injected to prime the column. The input for the prime and SEC experiment was kept at 950 μg of total protein amount (according to BCA). The sample was separated into 75 100-μl fractionations. For separation, the HPLC was cooled to 4 °C and the column was placed on ice. Column performance was monitored by injecting a standard protein mix (Phenome protein standard mixture, diluted 1:3 in SEC buffer) before and after the experiment.

### Preparation of SEC fractions by filter-aided sample preparation protocol

SEC fractions were further processed using a high-recovery and high-throughput filter-aided sample preparation protocol^124^. In short, the 96-well Acroprep advance filter plate (10,000-kDa MWCO) was equilibrated by flushing the plate twice with 100 μl of H_2_O (HPLC-grade) followed by centrifugation at 1,800*g*. Each fraction was diluted 1:1 (v/v) with SEC buffer and added to the filter plate. Additionally an aliquot of SEC input was added to the filter plate. The sample was loaded by centrifugation at 1,800*g* to completely remove the SEC buffer. Next, 50 μl of TUA buffer (5 mM TCEP, 8 M urea and 20 mM ammonium bicarbonate) was added to each fraction. The plate was incubated for 30 min at 400 rpm at 37 °C. After cooling the plate to room temperature, 20 μl of 35 mM IAA was added to each well and the plate was incubated for 1 h at 400 rpm at room temperature. Urea, IAA and TCEP were removed by centrifugation at 1,800*g* and the plate was washed three times with 100 μl of 20 mM ammonium bicarbonate with centrifugation steps in between. After the last centrifugation, there was less than 10 μl of 20 mM ammonium bicarbonate left in each well. Before adding the digestion mix, the filter plate was moved to a new receiver plate. Proteins were digested overnight at 300 rpm at 37 °C by adding 1 μg of trypsin and 0.3 μg of Lys-C diluted in 50 μl of 20 mM ammonium bicarbonate to each well. Peptides were collected by centrifugation at 2,400*g* for 30 min. The filter plate was subsequently washed with 100 μl of of HPLC-grade H_2_O. To increase the recovery of hydrophobic peptides, 50 μl of 50% acetonitrile was added to the receiver plate. Each fraction was then transferred to LoBind tubes and peptides were dried under reduced pressure at 42 °C.

### MS data analysis of SEC fractions

For MS analysis, peptides were reconstituted in 5% acetonitrile and 0.1% formic acid containing iRT peptides (Biognosys). The peptides were analyzed on a Fusion Lumos MS instrument (Thermo Fisher Scientific) using a 2-cm Acclaim PepMap 100 C18 HPLC trap column (Thermo Fisher Scientific) and 25-cm EASY-Spray HPLC analytical column (Thermo Fisher Scientific) setup connected to an EASY-nLC 1200 instrument (Thermo Fisher Scientific)^126,127^. Peptides were loaded in 100% buffer A (98% H_2_O, 2% acetonitrile and 0.15% formic acid) and eluted at a flow rate of 250 nl min^−1^ with a segmented 1-h gradient from 1% to 58% buffer B (80% acetonitrile and 0.15% formic acid). The data were acquired in data-independent acquisition mode. The Orbitrap-based method used contained 40 dynamic windows over a scan range of 350–1,650 *m*/*z* with a resolution of 30,000 and higher-energy collision-induced dissociation energy of 27% and a survey scan with a resolution of 120,000, maximum injection time of 50 ms and default charge state of 2. The MS proteomics data were deposited to the ProteomeXchange^128^ Consortium through the PRIDE^129^ partner repository with the dataset identifier PXD049084.

### Preprocessing of synaptosome cofractionation data

Raw files were converted to HTRMS using HTRMSConverter (Biognosys) and analyzed in Spectronaut 13 (Biognosys) applying the ‘only protein group-specific’ proteotypicity filter; otherwise, the standard manufacturer’s settings for directDIA were applied using the UniProt reference proteome for *Rattus norvegicus* (retrieved October 2019), quality control standards and common laboratory contaminants. The Spectronaut output (BGS report) was then further preprocessed (Supplementary Table 21). The total sum of intensity of the fractions was scaled to account for different dilutions applied at the MS injection stage (fractions 44–54 were multiplied by 2 and fractions 56–75 were multiplied by 4). Proteins without measurements were removed and fractions were removed when the total sum of intensity was less than 50% of the average total sum of intensity for the adjacent ten fractions (fractions 44, 45, 50, 51, 63 and 69). Rat protein identifiers were converted to human orthologs (Ensembl BioMart, September 26, 2023). The data were then further preprocessed as described earlier. The processed protein information and protein–protein associations are available through the BioStudies repository for this paper (S-BSST1423).

### Predicting a synaptic interactome from cofractionation data in the brain

Cofractionation data from in vivo mouse brains (110 fractions)^6^ and a subcellular fractionation of a human glioblastoma cell line (U251, 10 fractions)^7^ were preprocessed as described earlier. For each study and the in vivo rat synaptosomes (75 fractions), the correlations of the preprocessed intensities were computed per protein pair across fractions as described earlier (Supplementary Table 22). The correlation estimates of the studies were then filtered for protein pairs that were quantified for all three studies (1,309,771 pairs) and converted to association probabilities using a logistic model and all three studies as variates (model weights = 1.00 (glioblastoma), 2.05 (mouse brain) and 0.61 (rat synaptosome)), with complex members as positives, as described earlier. AUC values for the recovery of known interactions were computed using the association probabilities for each study individually and for the combined studies, as described earlier.

### Analysis for figure panels

Methodology for the analyses of figure panels is described in the Supplementary Methods. The source code and all data are available through the BioStudies repository for this paper (S-BSST1423).

### List of materials

**Table:** 

**Cell lysis, SEC and sample preparation**		
Compound	Catalog number	Supplier
HEPES, BioPerformance ≥ 99.5	H4034	Sigma-Aldrich
Sodium chloride	1.06404.5000_P	Merck
Sodium fluoride	S7920	Sigma-Aldrich
Sodium orthovanadate	S6506	Sigma-Aldrich
PMSF	P7625	Sigma
Protease inhibitor cocktail	P8849	Sigma
DDM	D4641	Sigma-Aldrich
Column performance check standard aqueous SEC 1	AL0-3042	Phenomenex
BCA assay kit	23225	Thermo Fisher Scientific
Ultracentrifuge tubes (8 × 34 mm)	343776	Beckman-Coulter
TLA 100.1 rotor		Beckman-Coulter
Amicon(R) Ultra-4, 30-kDa MWCO	UFC803096	Merck
Guard column, SRT-C1000, 5 μm, 1,000 Å, 7.8 × 50 mm	235950-7805	Sepax
SRT-C1000, 5 μm, 1,000 Å, 7.8 × 300 mm	215950-7830	Sepax
LC high-pressure pump	1100 Series	Agilent
Degasser	1100 Series	Agilent
LC fraction collectors	1260 Infinity Series	Agilent
LC operating software Agilent		OpenLab
AcroPrep Advance 96-well filter plates, 350 μl, Omega 10-kDa MWCO	8164	Pall
HPLC-grade H_2_O	7732-18-5	Fisher Chemical
Urea	GEPURE00-67	Eurobio
TCEP	C4706	Sigma-Aldrich
IAA	I6125	Sigma
Ammonium bicarbonate	09830	Fluka
Sequencing-grade modified trypsin	V5113	Promega AG
Lysyl endopeptidase (MS-grade)	WA3 125-05061	Wako
Acetonitrile (LC–MS-grade)	75-05-8	Fisher Chemical
LoBind tubes	022431081	Eppendorf
iRT kit	Ki-3002-2	Biognosys

### Reporting summary

Further information on research design is available in the Nature Portfolio Reporting Summary linked to this article.

## Online content

Any methods, additional references, Nature Portfolio reporting summaries, source data, extended data, supplementary information, acknowledgements, peer review information; details of author contributions and competing interests; and statements of data and code availability are available at 10.1038/s41587-025-02659-z.

## Supplementary information

Supplementary Information Supplementary Methods and Figs. 1–22.

Reporting Summary

Supplementary Tables 1–28 All supplementary tables and file descriptions.

## Source data

Source Data Fig. 1 Statistical source data.

Source Data Fig. 2 Statistical source data.

Source Data Fig. 3 Statistical source data.

Source Data Fig. 4 Statistical source data.

Source Data Fig. 5 Statistical source data.

## Data Availability

All processed, analyzed and generated data in this study are publicly available (BioStudies S-BSST1423). The MS proteomics data were deposited to the PRIDE repository (identifier PXD049084). Other proteomics data were collected from public studies (Supplementary Table 1). Approved HGNC gene symbols obtained online (https://www.genenames.org) and gene symbols were harmonized or converted to human orthologs with Ensembl (https://www.ensembl.org). Evidence for protein interactions was collected from STRING (https://string-db.org), CORUM (https://www.helmholtz-munich.de), HuMAP2 (http://humap2.proteincomplexes.org), HuRI (http://www.interactome-atlas.org), BioPlex (https://bioplex.hms.harvard.edu), IntAct (https://www.ebi.ac.uk/intact), Reactome (https://reactome.org) and Signor (https://signor.uniroma2.it). Evidence for disease-related genes was obtained from the OTAR genetics platform (https://platform.opentargets.org/). GO annotations were collected for SynGO (https://www.syngoportal.org) and other ontology terms (https://geneontology.org) and associations of genes with GO terms were collected using the UniProt rest API (https://www.uniprot.org) and EBI (https://www.ebi.ac.uk/QuickGO). Mutation frequencies in cancer were collected from cBioPortal (https://www.cbioportal.org). Consensus RNA expression data (https://www.proteinatlas.org/humanproteome/tissue/data#consensus_tissues_rna) and protein location data (https://www.proteinatlas.org/humanproteome/subcellular/data#locations) were collected from The Protein Atlas (https://www.proteinatlas.org). Source data are provided with this paper.

## Code availability

All code for data processing, analysis and the figures in this study is publicly available as Jupyter notebooks (BioStudies S-BSST1423).

## References

[1] Drew, K., Wallingford, J. B. & Marcotte, E. M. hu.MAP 2.0: integration of over 15,000 proteomic experiments builds a global compendium of human multiprotein assemblies. *Mol. Syst. Biol.* **17**, e10016 (2021). 33973408 10.15252/msb.202010016 PMC8111494

[2] Tarassov, K. et al. An in vivo map of the yeast protein interactome. *Science* **320**, 1465–1470 (2008). 18467557 10.1126/science.1153878

[3] Luck, K. et al. A reference map of the human binary protein interactome. *Nature* **580**, 402–408 (2020). 32296183 10.1038/s41586-020-2188-x PMC7169983

[4] Huttlin, E. L. et al. Dual proteome-scale networks reveal cell-specific remodeling of the human interactome. *Cell* **184**, 3022–3040 (2021). 33961781 10.1016/j.cell.2021.04.011 PMC8165030

[5] Hein, M. Y. et al. A human interactome in three quantitative dimensions organized by stoichiometries and abundances. *Cell* **163**, 712–723 (2015). 26496610 10.1016/j.cell.2015.09.053

[6] Skinnider, M. A. et al. An atlas of protein–protein interactions across mouse tissues. *Cell* **184**, 4073–4089 (2021). 34214469 10.1016/j.cell.2021.06.003

[7] Orre, L. M. et al. SubCellBarCode: proteome-wide mapping of protein localization and relocalization. *Mol. Cell* **73**, 166–182 (2019). 30609389 10.1016/j.molcel.2018.11.035

[8] Havugimana, P. C. et al. A census of human soluble protein complexes. *Cell* **150**, 1068–1081 (2012). 22939629 10.1016/j.cell.2012.08.011 PMC3477804

[9] Orchard, S. et al. The MIntAct project—IntAct as a common curation platform for 11 molecular interaction databases. *Nucleic Acids Res.* **42**, D358–D363 (2014). 24234451 10.1093/nar/gkt1115 PMC3965093

[10] Oughtred, R. et al. The BioGRID database: a comprehensive biomedical resource of curated protein, genetic, and chemical interactions. *Protein Sci.* **30**, 187–200 (2021). 33070389 10.1002/pro.3978 PMC7737760

[11] Szklarczyk, D. et al. The STRING database in 2021: customizable protein–protein networks, and functional characterization of user-uploaded gene/measurement sets. *Nucleic Acids Res.* **49**, D605–D612 (2021). 33237311 10.1093/nar/gkaa1074 PMC7779004

[12] Uhlén, M. et al. Tissue-based map of the human proteome. *Science* **347**, 1260419 (2015). 25613900 10.1126/science.1260419

[13] Pierson, E. et al. Sharing and specificity of coexpression networks across 35 human tissues. *PLoS Comput. Biol.* **11**, e1004220 (2015). 25970446 10.1371/journal.pcbi.1004220 PMC4430528

[14] Greene, C. S. et al. Understanding multicellular function and disease with human tissue-specific networks. *Nat. Genet.* **47**, 569–576 (2015). 25915600 10.1038/ng.3259 PMC4828725

[15] Sousa, A. et al. Multi-omics characterization of interaction-mediated control of human protein abundance levels. *Mol. Cell. Proteomics* **18**, S114–S125 (2019). 31239291 10.1074/mcp.RA118.001280 PMC6692786

[16] Ryan, C. J., Kennedy, S., Bajrami, I., Matallanas, D. & Lord, C. J. A compendium of co-regulated protein complexes in breast cancer reveals collateral loss events. *Cell Syst.* **5**, 399–409 (2017). 29032073 10.1016/j.cels.2017.09.011 PMC5660599

[17] Lapek, J. D. et al. Detection of dysregulated protein-association networks by high-throughput proteomics predicts cancer vulnerabilities. *Nat. Biotechnol.* **35**, 983–989 (2017). 28892078 10.1038/nbt.3955 PMC5683351

[18] Wang, J. et al. Proteome profiling outperforms transcriptome profiling for coexpression based gene function prediction. *Mol. Cell. Proteomics* **16**, 121–134 (2017). 27836980 10.1074/mcp.M116.060301 PMC5217778

[19] Buljan, M. et al. A computational framework for the inference of protein complex remodeling from whole-proteome measurements. *Nat. Methods* **20**, 1523–1529 (2023). 37749212 10.1038/s41592-023-02011-w PMC10555833

[20] Roumeliotis, T. I. et al. Genomic determinants of protein abundance variation in colorectal cancer cells. *Cell Rep.* **20**, 2201–2214 (2017). 28854368 10.1016/j.celrep.2017.08.010 PMC5583477

[21] Kustatscher, G. et al. Co-regulation map of the human proteome enables identification of protein functions. *Nat. Biotechnol.* **37**, 1361–1371 (2019). 31690884 10.1038/s41587-019-0298-5 PMC6901355

[22] Taggart, J. C., Zauber, H., Selbach, M., Li, G.-W. & McShane, E. Keeping the proportions of protein complex components in check. *Cell Syst.* **10**, 125–132 (2020). 32105631 10.1016/j.cels.2020.01.004 PMC7195860

[23] Juszkiewicz, S. & Hegde, R. S. Quality control of orphaned proteins. *Mol. Cell* **71**, 443–457 (2018). 30075143 10.1016/j.molcel.2018.07.001 PMC6624128

[24] Gonçalves, E. et al. Widespread post-transcriptional attenuation of genomic copy-number variation in cancer. *Cell Syst.* **5**, 386–398 (2017). 29032074 10.1016/j.cels.2017.08.013 PMC5660600

[25] McShane, E. et al. Kinetic analysis of protein stability reveals age-dependent degradation. *Cell* **167**, 803–815 (2016). 27720452 10.1016/j.cell.2016.09.015

[26] Xu, N. et al. Integrated proteogenomic characterization of urothelial carcinoma of the bladder. *J. Hematol. Oncol.* **15**, 76 (2022). 35659036 10.1186/s13045-022-01291-7 PMC9164575

[27] Herbst, S. A. et al. Proteogenomics refines the molecular classification of chronic lymphocytic leukemia. *Nat. Commun.* **13**, 6226 (2022). 36266272 10.1038/s41467-022-33385-8 PMC9584885

[28] Jayavelu, A. K. et al. The proteogenomic subtypes of acute myeloid leukemia. *Cancer Cell* **40**, 301–317 (2022). 35245447 10.1016/j.ccell.2022.02.006 PMC12882723

[29] Kramer, M. H. et al. Proteomic and phosphoproteomic landscapes of acute myeloid leukemia. *Blood* **140**, 1533–1548 (2022). 35895896 10.1182/blood.2022016033 PMC9523374

[30] Stratmann, S. et al. Proteogenomic analysis of acute myeloid leukemia associates relapsed disease with reprogrammed energy metabolism both in adults and children. *Leukemia* **37**, 550–559 (2023). 36572751 10.1038/s41375-022-01796-7 PMC9991901

[31] Yang, M. et al. Proteogenomics and Hi-C reveal transcriptional dysregulation in high hyperdiploid childhood acute lymphoblastic leukemia. *Nat. Commun.* **10**, 1519 (2019). 30944321 10.1038/s41467-019-09469-3 PMC6447538

[32] Archer, T. C. et al. Proteomics, post-translational modifications, and integrative analyses reveal molecular heterogeneity within medulloblastoma subgroups. *Cancer Cell* **34**, 396–410 (2018). 30205044 10.1016/j.ccell.2018.08.004 PMC6372116

[33] Oh, S. et al. Integrated pharmaco-proteogenomics defines two subgroups in isocitrate dehydrogenase wild-type glioblastoma with prognostic and therapeutic opportunities. *Nat. Commun.* **11**, 3288 (2020). 32620753 10.1038/s41467-020-17139-y PMC7335111

[34] Petralia, F. et al. Integrated proteogenomic characterization across major histological types of pediatric brain cancer. *Cell* **183**, 1962–1985(2020). 33242424 10.1016/j.cell.2020.10.044 PMC8143193

[35] Wang, L.-B. et al. Proteogenomic and metabolomic characterization of human glioblastoma. *Cancer Cell* **39**, 509–528 (2021). 33577785 10.1016/j.ccell.2021.01.006 PMC8044053

[36] Yanovich-Arad, G. et al. Proteogenomics of glioblastoma associates molecular patterns with survival. *Cell Rep.* **34**, 108787 (2021). 33657365 10.1016/j.celrep.2021.108787

[37] Zhang, F. et al. Integrated proteogenomic characterization across major histological types of pituitary neuroendocrine tumors. *Cell Res.* **32**, 1047–1067 (2022). 36307579 10.1038/s41422-022-00736-5 PMC9715725

[38] Anurag, M. et al. Proteogenomic markers of chemotherapy resistance and response in triple-negative breast cancer. *Cancer Discov.* **12**, 2586–2605 (2022). 36001024 10.1158/2159-8290.CD-22-0200 PMC9627136

[39] Asleh, K. et al. Proteomic analysis of archival breast cancer clinical specimens identifies biological subtypes with distinct survival outcomes. *Nat. Commun.* **13**, 896 (2022). 35173148 10.1038/s41467-022-28524-0 PMC8850446

[40] Gong, T.-Q. et al. Proteome-centric cross-omics characterization and integrated network analyses of triple-negative breast cancer. *Cell Rep.* **38**, 110460 (2022). 35235781 10.1016/j.celrep.2022.110460

[41] Johansson, H. J. et al. Breast cancer quantitative proteome and proteogenomic landscape. *Nat. Commun.* **10**, 1600 (2019). 30962452 10.1038/s41467-019-09018-y PMC6453966

[42] Krug, K. et al. Proteogenomic landscape of breast cancer tumorigenesis and targeted therapy. *Cell* **183**, 1436–1456 (2020). 33212010 10.1016/j.cell.2020.10.036 PMC8077737

[43] Mertins, P. et al. Proteogenomics connects somatic mutations to signalling in breast cancer. *Nature* **534**, 55–62 (2016). 27251275 10.1038/nature18003 PMC5102256

[44] Li, C. et al. Integrated omics of metastatic colorectal cancer. *Cancer Cell* **38**, 734–747 (2020). 32888432 10.1016/j.ccell.2020.08.002

[45] Vasaikar, S. et al. Proteogenomic analysis of human colon cancer reveals new therapeutic opportunities. *Cell* **177**, 1035–1049 (2019). 31031003 10.1016/j.cell.2019.03.030 PMC6768830

[46] Wang, J. et al. Colorectal cancer cell line proteomes are representative of primary tumors and predict drug sensitivity. *Gastroenterology* **153**, 1082–1095 (2017). 28625833 10.1053/j.gastro.2017.06.008 PMC5623120

[47] Clark, D. J. et al. Integrated proteogenomic characterization of clear cell renal cell carcinoma. *Cell* **179**, 964–983 (2019). 31675502 10.1016/j.cell.2019.10.007 PMC7331093

[48] Li, Y. et al. Histopathologic and proteogenomic heterogeneity reveals features of clear cell renal cell carcinoma aggressiveness. *Cancer Cell* **41**, 139–163 (2023). 36563681 10.1016/j.ccell.2022.12.001 PMC9839644

[49] Qu, Y. et al. A proteogenomic analysis of clear cell renal cell carcinoma in a Chinese population. *Nat. Commun.* **13**, 2052 (2022). 35440542 10.1038/s41467-022-29577-x PMC9019091

[50] Qu, Y. et al. Proteogenomic characterization of MiT family translocation renal cell carcinoma. *Nat. Commun.* **13**, 7494 (2022). 36470859 10.1038/s41467-022-34460-w PMC9722939

[51] Dong, L. et al. Proteogenomic characterization identifies clinically relevant subgroups of intrahepatic cholangiocarcinoma. *Cancer Cell* **40**, 70–87 (2022). 34971568 10.1016/j.ccell.2021.12.006

[52] Gao, Q. et al. Integrated proteogenomic characterization of HBV-related hepatocellular carcinoma. *Cell* **179**, 561–577 (2019). 31585088 10.1016/j.cell.2019.08.052

[53] Gu, Y. et al. The proteomic characterization of the peritumor microenvironment in human hepatocellular carcinoma. *Oncogene* **41**, 2480–2491 (2022). 35314790 10.1038/s41388-022-02264-3 PMC9033583

[54] Jiang, Y. et al. Proteomics identifies new therapeutic targets of early-stage hepatocellular carcinoma. *Nature* **567**, 257–261 (2019). 30814741 10.1038/s41586-019-0987-8

[55] Ng, C. K. Y. et al. Integrative proteogenomic characterization of hepatocellular carcinoma across etiologies and stages. *Nat. Commun.* **13**, 2436 (2022). 35508466 10.1038/s41467-022-29960-8 PMC9068765

[56] Chen, Y.-J. et al. Proteogenomics of non-smoking lung cancer in East Asia delineates molecular signatures of pathogenesis and progression. *Cell* **182**, 226–244 (2020). 32649875 10.1016/j.cell.2020.06.012

[57] Gillette, M. A. et al. Proteogenomic characterization reveals therapeutic vulnerabilities in lung adenocarcinoma. *Cell* **182**, 200–225 (2020). 32649874 10.1016/j.cell.2020.06.013 PMC7373300

[58] Lehtiö, J. et al. Proteogenomics of non-small cell lung cancer reveals molecular subtypes associated with specific therapeutic targets and immune evasion mechanisms. *Nat Cancer* **2**, 1224–1242 (2021). 34870237 10.1038/s43018-021-00259-9 PMC7612062

[59] Satpathy, S. et al. A proteogenomic portrait of lung squamous cell carcinoma. *Cell* **184**, 4348–4371 (2021). 34358469 10.1016/j.cell.2021.07.016 PMC8475722

[60] Soltis, A. R. et al. Proteogenomic analysis of lung adenocarcinoma reveals tumor heterogeneity, survival determinants, and therapeutically relevant pathways. *Cell Rep. Med.* **3**, 100819 (2022). 36384096 10.1016/j.xcrm.2022.100819 PMC9729884

[61] Stewart, P. A. et al. Proteogenomic landscape of squamous cell lung cancer. *Nat. Commun.* **10**, 3578 (2019). 31395880 10.1038/s41467-019-11452-x PMC6687710

[62] Xu, J.-Y. et al. Integrative proteomic characterization of human lung adenocarcinoma. *Cell* **182**, 245–261 (2020). 32649877 10.1016/j.cell.2020.05.043

[63] McDermott, J. E. et al. Proteogenomic characterization of ovarian HGSC implicates mitotic kinases, replication stress in observed chromosomal instability. *Cell Rep. Med.* **1**, 100075 (2020). 32529193 10.1016/j.xcrm.2020.100004 PMC7289043

[64] Zhang, H. et al. Integrated proteogenomic characterization of human high-grade serous ovarian cancer. *Cell* **166**, 755–765 (2016). 27372738 10.1016/j.cell.2016.05.069 PMC4967013

[65] Cao, L. et al. Proteogenomic characterization of pancreatic ductal adenocarcinoma. *Cell* **184**, 5031–5052 (2021). 34534465 10.1016/j.cell.2021.08.023 PMC8654574

[66] Hyeon, D. Y. et al. Proteogenomic landscape of human pancreatic ductal adenocarcinoma in an Asian population reveals tumor cell-enriched and immune-rich subtypes. *Nat Cancer* **4**, 290–307 (2023). 36550235 10.1038/s43018-022-00479-7

[67] Sinha, A. et al. The proteogenomic landscape of curable prostate cancer. *Cancer Cell* **35**, 414–427 (2019). 30889379 10.1016/j.ccell.2019.02.005 PMC6511374

[68] Fan, Y. et al. Proteomic profiling of gastric signet ring cell carcinoma tissues reveals characteristic changes of the complement cascade pathway. *Mol. Cell. Proteomics* **20**, 100068 (2021). 33676000 10.1016/j.mcpro.2021.100068 PMC8121970

[69] Ge, S. et al. A proteomic landscape of diffuse-type gastric cancer. *Nat. Commun.* **9**, 1012 (2018). 29520031 10.1038/s41467-018-03121-2 PMC5843664

[70] Li, Y. et al. Proteomic characterization of gastric cancer response to chemotherapy and targeted therapy reveals new therapeutic strategies. *Nat. Commun.* **13**, 5723 (2022). 36175412 10.1038/s41467-022-33282-0 PMC9522856

[71] Huang, C. et al. Proteogenomic insights into the biology and treatment of HPV-negative head and neck squamous cell carcinoma. *Cancer Cell* **39**, 361–379 (2021). 33417831 10.1016/j.ccell.2020.12.007 PMC7946781

[72] Li, S. et al. Integrative proteomic characterization of adenocarcinoma of esophagogastric junction. *Nat. Commun.* **14**, 778 (2023). 36774361 10.1038/s41467-023-36462-8 PMC9922290

[73] Liu, W. et al. Large-scale and high-resolution mass spectrometry-based proteomics profiling defines molecular subtypes of esophageal cancer for therapeutic targeting. *Nat. Commun.* **12**, 4961 (2021). 34400640 10.1038/s41467-021-25202-5 PMC8368010

[74] Bateman, N. W. et al. Proteogenomic landscape of uterine leiomyomas from hereditary leiomyomatosis and renal cell cancer patients. *Sci. Rep.* **11**, 9371 (2021). 33931688 10.1038/s41598-021-88585-x PMC8087684

[75] Dou, Y. et al. Proteogenomic characterization of endometrial carcinoma. *Cell* **180**, 729–748 (2020). 32059776 10.1016/j.cell.2020.01.026 PMC7233456

[76] Ruepp, A. et al. CORUM: the comprehensive resource of mammalian protein complexes—2009. *Nucleic Acids Res.* **38**, D497–D501 (2010). 19884131 10.1093/nar/gkp914 PMC2808912

[77] Havugimana, P. C. et al. Scalable multiplex co-fractionation/mass spectrometry platform for accelerated protein interactome discovery. *Nat. Commun.* **13**, 4043 (2022). 35831314 10.1038/s41467-022-31809-z PMC9279285

[78] Luca, B. A. et al. Atlas of clinically distinct cell states and ecosystems across human solid tumors. *Cell* **184**, 5482–5496 (2021). 34597583 10.1016/j.cell.2021.09.014 PMC8526411

[79] Lo Surdo, P. et al. SIGNOR 3.0, the signaling network open resource 3.0: 2022 update. *Nucleic Acids Res.* **51**, D631–D637 (2023). 36243968 10.1093/nar/gkac883 PMC9825604

[80] Milacic, M. et al. The Reactome Pathway Knowledgebase 2024. *Nucleic Acids Res.* **52**, D672–D678 (2024). 37941124 10.1093/nar/gkad1025 PMC10767911

[81] Szklarczyk, D. et al. STRING v11: protein–protein association networks with increased coverage, supporting functional discovery in genome-wide experimental datasets. *Nucleic Acids Res.* **47**, D607–D613 (2019). 30476243 10.1093/nar/gky1131 PMC6323986

[82] Romanov, N. et al. Disentangling genetic and environmental effects on the proteotypes of individuals. *Cell* **177**, 1308–1318 (2019). 31031010 10.1016/j.cell.2019.03.015 PMC6988111

[83] Guardia, C. M., De Pace, R., Mattera, R. & Bonifacino, J. S. Neuronal functions of adaptor complexes involved in protein sorting. *Curr. Opin. Neurobiol.* **51**, 103–110 (2018). 29558740 10.1016/j.conb.2018.02.021 PMC6410744

[84] Koopmans, F. et al. SynGO: an evidence-based, expert-curated knowledge base for the synapse. *Neuron* **103**, 217–234 (2019). 31171447 10.1016/j.neuron.2019.05.002 PMC6764089

[85] Huang, L.-H. et al. Postprandial chylomicron output and transport through intestinal lymphatics are not impaired in active Crohn’s disease. *Gastroenterology* **159**, 1955–1957 (2020). 32681923 10.1053/j.gastro.2020.07.012 PMC7680355

[86] Ghoshal, S., Witta, J., Zhong, J., de Villiers, W. & Eckhardt, E. Chylomicrons promote intestinal absorption of lipopolysaccharides. *J. Lipid Res.* **50**, 90–97 (2009). 18815435 10.1194/jlr.M800156-JLR200

[87] Kopec, A. K. & Luyendyk, J. P. Role of fibrin(ogen) in progression of liver disease: guilt by association? *Semin. Thromb. Hemost.* **42**, 397–407 (2016). 27144445 10.1055/s-0036-1579655 PMC5338033

[88] Northup, P. G. & Caldwell, S. H. Coagulation in liver disease: a guide for the clinician. *Clin. Gastroenterol. Hepatol.* **11**, 1064–1074 (2013). 23506859 10.1016/j.cgh.2013.02.026

[89] Ashburner, M. et al. Gene Ontology: tool for the unification of biology. *Nat. Genet.* **25**, 25–29 (2000). 10802651 10.1038/75556 PMC3037419

[90] Ghoussaini, M. et al. Open Targets Genetics: systematic identification of trait-associated genes using large-scale genetics and functional genomics. *Nucleic Acids Res.* **49**, D1311–D1320 (2021). 33045747 10.1093/nar/gkaa840 PMC7778936

[91] Mountjoy, E. et al. An open approach to systematically prioritize causal variants and genes at all published human GWAS trait-associated loci. *Nat. Genet.* **53**, 1527–1533 (2021). 34711957 10.1038/s41588-021-00945-5 PMC7611956

[92] Yan, J. et al. The prevalence and comorbidity of tic disorders and obsessive–compulsive disorder in Chinese school students aged 6–16: a national survey. *Brain Sci.* **12**, 650 (2022). 35625036 10.3390/brainsci12050650 PMC9139904

[93] Kumar, A., Trescher, W. & Byler, D. Tourette syndrome and comorbid neuropsychiatric conditions. *Curr. Dev. Disord. Rep.* **3**, 217–221 (2016). 27891299 10.1007/s40474-016-0099-1 PMC5104764

[94] Davies, M. et al. ChEMBL web services: streamlining access to drug discovery data and utilities. *Nucleic Acids Res.* **43**, W612–W620 (2015). 25883136 10.1093/nar/gkv352 PMC4489243

[95] Dickinson, M. E. et al. High-throughput discovery of novel developmental phenotypes. *Nature* **537**, 508–514 (2016). 27626380 10.1038/nature19356 PMC5295821

[96] Sadler, M. C., Auwerx, C., Deelen, P. & Kutalik, Z. Multi-layered genetic approaches to identify approved drug targets. *Cell Genom.* **3**, 100341 (2023). 37492104 10.1016/j.xgen.2023.100341 PMC10363916

[97] Barrio-Hernandez, I. et al. Network expansion of genetic associations defines a pleiotropy map of human cell biology. *Nat. Genet.* **55**, 389–398 (2023). 36823319 10.1038/s41588-023-01327-9 PMC10011132

[98] Lee, I., Blom, U. M., Wang, P. I., Shim, J. E. & Marcotte, E. M. Prioritizing candidate disease genes by network-based boosting of genome-wide association data. *Genome Res.* **21**, 1109–1121 (2011). 21536720 10.1101/gr.118992.110 PMC3129253

[99] Hsu, Y.-H. H. et al. Using brain cell-type-specific protein interactomes to interpret neurodevelopmental genetic signals in schizophrenia. *iScience* **26**, 106701 (2023). 37207277 10.1016/j.isci.2023.106701 PMC10189495

[100] Pintacuda, G. et al. Protein interaction studies in human induced neurons indicate convergent biology underlying autism spectrum disorders. *Cell Genom.* **3**, 100250 (2023). 36950384 10.1016/j.xgen.2022.100250 PMC10025425

[101] O’Neill, A. C. et al. Spatial centrosome proteome of human neural cells uncovers disease-relevant heterogeneity. *Science* **376**, eabf9088 (2022). 35709258 10.1126/science.abf9088

[102] Drummond, E. et al. Phosphorylated tau interactome in the human Alzheimer’s disease brain. *Brain* **143**, 2803–2817 (2020). 32812023 10.1093/brain/awaa223 PMC7526722

[103] Tracy, T. E. et al. Tau interactome maps synaptic and mitochondrial processes associated with neurodegeneration. *Cell* **185**, 712–728 (2022). 35063084 10.1016/j.cell.2021.12.041 PMC8857049

[104] Runge, K. et al. Neurodegeneration markers in the cerebrospinal fluid of 100 patients with schizophrenia spectrum disorder. *Schizophr. Bull.* **49**, 464–473 (2023). 36200879 10.1093/schbul/sbac135 PMC10016411

[105] Lewis, A. S. et al. Alternatively spliced isoforms of TRIP8b differentially control h channel trafficking and function. *J. Neurosci.* **29**, 6250–6265 (2009). 19439603 10.1523/JNEUROSCI.0856-09.2009 PMC2730639

[106] Santoro, B. et al. TRIP8b regulates HCN1 channel trafficking and gating through two distinct C-terminal interaction sites. *J. Neurosci.* **31**, 4074–4086 (2011). 21411649 10.1523/JNEUROSCI.5707-10.2011 PMC3077297

[107] Popova, N. V., Plotnikov, A. N., Ziganshin, R. K., Deyev, I. E. & Petrenko, A. G. Analysis of proteins interacting with TRIP8b adapter. *Biochemistry* **73**, 644–651 (2008). 18620529 10.1134/s0006297908060035

[108] van Oostrum, M. et al. The proteomic landscape of synaptic diversity across brain regions and cell types. *Cell* **186**, 5411–5427 (2023). 37918396 10.1016/j.cell.2023.09.028 PMC10686415

[109] Roth, F. C. & Hu, H. An axon-specific expression of HCN channels catalyzes fast action potential signaling in GABAergic interneurons. *Nat. Commun.* **11**, 2248 (2020). 32382046 10.1038/s41467-020-15791-y PMC7206118

[110] Cai, W., Liu, S.-S., Li, B.-M. & Zhang, X.-H. Presynaptic HCN channels constrain GABAergic synaptic transmission in pyramidal cells of the medial prefrontal cortex. *Biol. Open* **11**, bio058840 (2022). 34709375 10.1242/bio.058840 PMC8966777

[111] Kaar, S. J., Angelescu, I., Marques, T. R. & Howes, O. D. Pre-frontal parvalbumin interneurons in schizophrenia: a meta-analysis of post-mortem studies. *J. Neural Transm.* **126**, 1637–1651 (2019). 31529297 10.1007/s00702-019-02080-2 PMC6856257

[112] Arime, Y. et al. Activation of prefrontal parvalbumin interneurons ameliorates working memory deficit even under clinically comparable antipsychotic treatment in a mouse model of schizophrenia. *Neuropsychopharmacology* **49**, 720–730 (2024). 38049583 10.1038/s41386-023-01769-z PMC10876596

[113] Hashimoto, T. et al. Gene expression deficits in a subclass of GABA neurons in the prefrontal cortex of subjects with schizophrenia. *J. Neurosci.* **23**, 6315–6326 (2003). 12867516 10.1523/JNEUROSCI.23-15-06315.2003 PMC6740534

[114] Lewis, D. A., Hashimoto, T. & Volk, D. W. Cortical inhibitory neurons and schizophrenia. *Nat. Rev. Neurosci.* **6**, 312–324 (2005). 15803162 10.1038/nrn1648

[115] Bitanihirwe, B. K. Y., Lim, M. P., Kelley, J. F., Kaneko, T. & Woo, T. U. W. Glutamatergic deficits and parvalbumin-containing inhibitory neurons in the prefrontal cortex in schizophrenia. *BMC Psychiatry* **9**, 71 (2009). 19917116 10.1186/1471-244X-9-71 PMC2784456

[116] Burke, D. F. et al. Towards a structurally resolved human protein interaction network. *Nat. Struct. Mol. Biol.* **30**, 216–225 (2023). 36690744 10.1038/s41594-022-00910-8 PMC9935395

[117] Madeira, F. et al. 14-3-3-Pred: improved methods to predict 14-3-3-binding phosphopeptides. *Bioinformatics* **31**, 2276–2283 (2015). 25735772 10.1093/bioinformatics/btv133 PMC4495292

[118] Lankford, C., Houtman, J. & Baker, S. A. Identification of HCN1 as a 14-3-3 client. *PLoS ONE* **17**, e0268335 (2022). 35679272 10.1371/journal.pone.0268335 PMC9182292

[119] Gonzalez-Lozano, M. A. et al. Stitching the synapse: cross-linking mass spectrometry into resolving synaptic protein interactions. *Sci. Adv.* **6**, eaax5783 (2020). 32128395 10.1126/sciadv.aax5783 PMC7030922

[120] Moon, H. M., Hippenmeyer, S., Luo, L. & Wynshaw-Boris, A. LIS1 determines cleavage plane positioning by regulating actomyosin-mediated cell membrane contractility. *eLife* **9**, e51512 (2020). 32159512 10.7554/eLife.51512 PMC7112955

[121] Skinnider, M. A. & Foster, L. J. Meta-analysis defines principles for the design and analysis of co-fractionation mass spectrometry experiments. *Nat. Methods* **18**, 806–815 (2021). 34211188 10.1038/s41592-021-01194-4

[122] Frommelt, F. et al. DIP-MS: ultra-deep interaction proteomics for the deconvolution of protein complexes. *Nat. Methods* **21**, 635–647 (2024). 38532014 10.1038/s41592-024-02211-y PMC11009110

[123] Liu, Y. et al. Multi-omic measurements of heterogeneity in HeLa cells across laboratories. *Nat. Biotechnol.* **37**, 314–322 (2019). 30778230 10.1038/s41587-019-0037-y

[124] Fossati, A. et al. System-wide profiling of protein complexes via size exclusion chromatography–mass spectrometry (SEC–MS). In *Shotgun Proteomics: Methods and Protocols* (eds Carrera, M. & Mateos, J.) (Springer, 2021). 10.1007/978-1-0716-1178-4_18 33687722

[125] Bludau, I. et al. Complex-centric proteome profiling by SEC–SWATH–MS for the parallel detection of hundreds of protein complexes. *Nat. Protoc.* **15**, 2341–2386 (2020). 32690956 10.1038/s41596-020-0332-6

[126] Wildschut, M. H. E. et al. Proteogenetic drug response profiling elucidates targetable vulnerabilities of myelofibrosis. *Nat. Commun.* **14**, 6414 (2023). 37828014 10.1038/s41467-023-42101-z PMC10570306

[127] Muntel, J. et al. Comparison of protein quantification in a complex background by DIA and TMT workflows with fixed instrument time. *J. Proteome Res.* **18**, 1340–1351 (2019). 30726097 10.1021/acs.jproteome.8b00898

[128] Deutsch, E. W. et al. The ProteomeXchange consortium at 10 years: 2023 update. *Nucleic Acids Res.* **51**, D1539–D1548 (2023). 36370099 10.1093/nar/gkac1040 PMC9825490

[129] Perez-Riverol, Y. et al. The PRIDE database resources in 2022: a hub for mass spectrometry-based proteomics evidences. *Nucleic Acids Res.* **50**, D543–D552 (2022). 34723319 10.1093/nar/gkab1038 PMC8728295

