# Supplementary material for: A tissue-specific atlas of protein–protein associations enables prioritization of candidate disease genes
Source: Nat Biotechnol. 2025 May 2;44(4):654–67. doi: 10.1038/s41587-025-02659-z (PMC13090126; doi:10.1038/s41587-025-02659-z)
Supplement: Supplementary file 1 — Supplementary Methods and Figs. 1–22. [file 41587_2025_2659_MOESM1_ESM.pdf]

nature biotechnology

Supplementary information https://doi.org/10.1038/s41587-025-02659-z

A tissue-specific atlas of protein–protein associations enables prioritization of candidate disease genes

In the format provided by the authors and unedited

SUPPLEMENTARY METHODS

Homogenizing protein identifiers across cohorts

Protein abundance measurements were primarily reported at the gene-level and using gene symbols as gene identifiers (45 of 50 cohorts). Gene identifiers were identified, checked, and corrected where possible to maximize the number of overlapping genes between cohorts. Identifiers that contained multiple gene names were split into individual replicate measurements (for 12 of 50 cohorts). All gene identifiers that were aliases, previous symbols, Ensembl IDs or UniProt IDs were converted to the approved HGNC gene symbols (using HGNC dataset and Ensembl Biomart; Table S15). Thus, gene identifiers were not changed if they were already an approved HGNC gene symbol. Identifiers that mapped to multiple approved gene symbols were ignored, and symbols were corrected for common errors made with gene symbols (e.g., replacing 'Sep.01' with 'SEPTIN1'). Finally, identifiers that could not be mapped to approved HGNC gene symbols and that occurred in at least 5 cohorts were kept with their original identifier. Overall, we mapped 99.5% of the reported gene identifiers in the cohorts to 16,986 unique gene symbols.

Combinations of RNA co-expression and protein co-abundance data (Fig. 1d-e)

Studies were selected for having both RNA expression and protein abundance data available (32 studies - 29 studies had at least 30 shared samples). For each of these studies, both the RNA and protein abundance data was filtered for proteins and samples quantified for both modalities. The RNA expression was then regressed out of the protein abundance data to exclude the variation of protein abundance that can be explained by gene expression ('protein - RNA'). Specifically, a linear model (linear regression with intercept) was fitted for each protein to find the dependence of protein abundance ($A_{protein}$) on RNA expression (A_{RNA}), ignoring missing values,

$$A_{protein} \sim \beta_0 + \beta_1 \cdot A_{RNA} + \varepsilon.$$

Here, ε represents the variation of protein abundance that cannot be explained by gene expression, and β_0,β_1 are the parameters of the linear model. The gene expression was then regressed out of the protein abundance by subtracting the model's prediction ($A_{pred} = \beta_0 + \beta_1 \cdot A_{RNA}$) from the protein abundance for all values,

$$A_{protein - RNA} = A_{protein} - A_{pred} = \varepsilon.$$

Note that the inclusion of an intercept does not affect the co-abundance estimates as correlation coefficients are invariant under linear transformations. Co-abundance estimates and association probabilities were then computed following 'Computing association probabilities' (Methods).

Additionally, for the same studies, the RNA expression and protein abundance data were combined to predict association probabilities ('protein + RNA'). Specifically, the correlation estimates for both the RNA co-expression and protein co-abundance of the studies were filtered for protein pairs quantified for both modalities. The correlation estimates were then converted to association probabilities using a logistic model and the correlation values of the two modalities as variates. Specifically, the correlations from RNA co-expression $\{x_i\}_{i \in [1,n]}$ and protein

co-abundance $\{x_i\}_{i \in [1,n]}$ of the n protein pairs were combined through a logistic model modified from 'Computing association probabilities' (Methods) with the labels $\{y_i\}_{i \in [1,n]}$ defined as before,

$$logit\, p(y_i \mid x_{RNA,i}, x_{protein,i}) = \beta_0 + \beta_{RNA} \cdot x_{RNA,i} + \beta_{protein} \cdot x_{protein,i}.$$

For each study having RNA and protein abundance data, the protein pairs were filtered for having association probabilities for both modalities and their combinations. AUC values were then computed following 'Computing ROC curves and AUC values' (Methods) for these filtered sets of association probabilities. AUC values were also computed for the co-fractionation studies (from 'Computing interaction probabilities from co-fractionation data' (Methods)). Protein pairs of co-fractionation studies were not filtered as they were separate studies from the RNA co-expression and protein co-abundance studies.

Recovering tissue-specific associations (Figs. 1g & 2c)

Cohorts were filtered for protein pairs having association probabilities in all cohorts. The recovery of tissue-specific associations was then scored through a hold-one-out methodology as follows. Each cohort was sequentially used as a hold-out by not using its association scores for predicting tissue-specific associations. Using all other cohorts, tissue-level association probabilities were computed, for each tissue, as the averaged probabilities of the cohorts. Associations of protein pairs were defined as tissue-specific if the tissue-level association probability exceeded the 95-th percentile for a given tissue and remained below 0.5 when averaged over all other tissues. The withheld study was then used to recover the tissue-specific associations of each tissue following 'Computing ROC curves and AUC values' (Methods), resulting in AUC values for all pairs of studies and tissues. Recovery of tissue-specific associations for a tissue with the cohorts from some (other) tissue was then summarized at the tissue-level by averaging the AUC scores of the respective cohorts.

Tissue-specific associations were recovered for the tissue-level scores from tumor- and healthy-derived biopsies (Fig. 2c) in analogous fashion. Tissues were filtered for protein pairs having association scores across all studies for both the tumor- and healthy-derived scores. Tissue-specific associations were predicted using the association scores derived from healthy samples. Here, the tissue-specific associations were defined as the protein pairs whose association scores exceeded the 95-th percentile in one healthy-derived tissue and remained below 0.5 when averaged over the other healthy-derived tissues. For each tissue, these tissue-specific associations were then recovered using the tumor-derived association scores.

Differences of likely associations between tissues (Fig. 2e)

Pairs of tissues (e.g., tissues A and B) were filtered for protein pairs quantified for both tissues. Let $S_X(\tau)$ be the set of protein pairs whose association scores exceed threshold τ in tissue X. The probability to be likely between tissues A and B, given threshold τ, was then defined as,

$$P_{A\rightarrow B}(likely \mid \tau) = \frac{|S_A(\tau) \cap S_B(0.5)|}{|S_A(\tau)|}.$$

In other words, the probability to be likely between tissues represents the probability of associations being likely in tissue B while having scores exceeding some threshold τ in another tissue A. These probabilities were computed across a range of score thresholds and for all pairs of tissues. Probabilities were computed analogously for the six pairs of tumor- and healthy-derived association scores.

Similarity of likely associations between tissues (Fig. 2f)

For each pair of tissues, the associations were filtered for having prior evidence (or no evidence) and being quantified for both tissues. The similarity between tissues was then the Jaccard index for the sets of remaining associations that were likely in each tissue. Specifically, let $S_X(\tau)$ be the set of protein pairs having prior evidence whose association scores exceed threshold τ in tissue X. The similarity between tissues A and B was then computed as,

$$J_{A,B} = \frac{\left|S_A(0.5) \cap S_B(0.5)\right|}{\left|\left(S_A(0) \cap S_B(0.5)\right) \cup \left(S_A(0.5) \cap S_B(0)\right)\right|}.$$

As prior evidence, interactions were considered from HuRI (date = 2024-08-28) [1], BioPlex (combined interaction networks for 239T, HCT116 (both 2022-09-22) and U2OS, RPE1 (both 2023-04-05)) [2], Signor complete (release July 2024) [3], Reactome (human interactions, 2024-08-28, [4]), STRING (human physical interactions with scores >= 400 – v12, 2023-12-01) [5], and CORUM (2022-09-20) [6].

Context-dependent associations for protein complexes (Fig. 3a)

Association scores were filtered for protein pairs quantified for all tissues. Proteins were filtered for having associations with all subunits of the AP-2 complex, and further filtered for the 91 proteins that were known to interact with at least one AP-2 subunit (STRING scores > 400). These 91 known interactors were then divided into the 51 synaptic and 39 other (non-synaptic) proteins (following SynGO [7], release 2023-12-01). The distribution of association scores of the synaptic proteins with the AP-2 complex were then compared between the brain and the other tissues (one-sided MWU-test). The distributions for non-synaptic proteins were compared following the same methodology. The known interactors were then filtered for having, on average, likely associations with the AP-2 complex subunits in the brain (synaptic interactors) or the other tissues on average (non-synaptic interactors). Finally, the association scores of the known interactors with the AP-2 subunits were averaged at the complex level (Table S23). The AP-2 complex was then visualized with the known interactors that had the largest complex-level difference in association scores between the brain and the other tissues, taking synaptic interactors that had higher association scores in the brain and non-synaptic interactors that had higher association scores in the other tissues.

Disease-relevant associations for protein complexes (Fig. 3b)

The disease genes for Anemia (blood), Crohn's disease (colon) and Liver disease (liver) were selected by merging together the genes associated with each disease through GWAS (OTAR L2G >= 0.5), drug targets (ChEBML clinical stage I or higher) or mouse phenotypes (IMPC score >= 0.5). Protein complexes related to each disease (hemoglobin (GO:000583 - for blood), chylomicron (GO:0042627 - for colon) and fibrinogen (GO:0005577 - for liver)) were defined through their Gene Ontology (GO) annotations (date 2024-08-27 [8]), converted to the HGNC

gene symbols (Supplementary Methods) and filtered for proteins quantified in the relevant tissue of the association atlas. Each complex was further filtered for subunits that had known interactions (STRING scores >= 900) with the other subunits of the same complex. The resulting complexes consisted of subunits quantified in the relevant tissue of the association atlas having known interactions between its subunits. Each complex contained genes associated with the disease matched for the tissue. For each complex, the association scores of each tissue were filtered for involving proteins that are complex members. For each defined complex, all possible pairs of subunits had associations quantified in the association atlas and interactions reported in STRING. Complexes were annotated by known interactions (STRING scores > 400 - minimum STRING score between subunits of defined complexes were 540 (hemoglobin), 852 (chylomicron), and 720 (fibrinogen)). Complexes were further annotated by associations that were likely in all tissues (average association score across tissues between subunits of defined complexes were 0.84 (hemoglobin), 0.82 (chylomicron), and 0.80 (fibrinogen)). Disease genes were then selected for having the most tissue-specific associations with the subunits for each complex. Specifically, disease genes were required to have associations with complex subunits, with scores exceeding 0.5 in the relevant tissue and being below 0.5 in all other tissues, further filtering for genes having at least 4 such associations for the blood and colon, and at least 2 for the liver). Finally, the remaining disease genes were selected for having the largest difference in association score between the relevant tissue and the maximum score for other tissues. The two disease genes with the most tissue-specific associations were shown for each complex.

Specificity and preservation of relationship scores (Fig. 3d)

For each tissue, the relationship scores between pairs of cellular components, between pairs of traits, and between traits and components (from 'Trait- and component-level association scores and relationship scores of traits and components' (Methods)) were normalized with the median association score of the tissue, and filtered for relationships for all tissues. As examples, relationships were then filtered for pairs of cellular components whose relative score averaged across tissues exceeded 1.75, and for which the name of at least one component contained 'ribosom', 'spliceosome' or 'snRNP'. Alternatively, relationships were filtered for pairs of cellular components whose coefficient of variation across tissues exceeded 0.4 and for which the name of at least one component contained 'myelin', 'synap', 'axo' (not 'axonem'), 'neuro', 'dendrit', 'node of Ranvier' or 'calyx of Held'.

Relationship scores for prioritizing genes associated with OCD (Fig. 3e)

Traits were filtered for having at least 100 associations between its associated proteins. The trait-level association scores (from 'Trait- and component-level association scores and relationship scores of traits and components' (Methods)) were normalized with the tissue-median association score for each tissue and then z-scored across tissues. The top 15 traits most specific to the brain were selected by taking the traits with the highest z-score for the brain tissue (Table S7). The trait-trait relationship scores of the brain were then filtered for all relationships of the brain-specific traits. The resulting matrix scored the relationships of the brain-specific traits with all other traits, and was used to cluster the brain-specific traits. Next, the cellular components that had relationship scores with traits were filtered for having at least 400 associations used to compute the relationship scores (517 components). The remaining

relationship scores were z-scored across tissues to select the relationships between cellular components and traits most specific to the brain. From these, the top 15 cellular components were selected that had the highest brain-specific relationship with OCD. Finally, the component-component relationship scores of the brain were filtered for the relationships of these top 15 components. The resulting matrix scored the relationships of the OCD-related components with all other components, and was used to cluster the cellular components.

The genes associated to each of the selected cellular components (from 'Proteins associated with GO cellular components' (Methods)) and the genes associated to OCD (GWAS L2G >= 0.5 - from 'Proteins associated with human traits through GWAS variants, drug targets or mouse phenotypes' (Methods)) were used to compute the jaccard index for the genes that the components had in common with OCD. Enrichment of other OCD-related genes in the OCD-related components was then tested with one-sided Fisher exact tests. Other OCD-related genes were defined through drug targets (ChEMBL clinical stage II and higher), genes associated with OCD in mice (IMPC scores >= 0.5) or genes with weak evidence supporting them as causal for OCD (OTAR L2G < 0.5). The collection of all other genes reported in each database was used as a background for the different sources of OCD-associated genes and for the OCD-related cellular components respectively. The genes confidently associated with OCD through GWAS (OTAR L2G >= 0.5) were removed from all sets of genes before testing (these genes were used to score the relationships between OCD and cellular compartments). Finally, the enrichment of the other OCD-related genes in all other cellular components (not selected as having the highest brain-specific relationship with OCD) was tested in similar fashion (Table S24). To do so, the cellular components were filtered for the ones that contained OCD-related genes from a given database after removing the genes confidently associated with OCD through GWAS as before. The level of enrichment of OCD-associated genes in OCD-related components was then compared with the enrichment in the other components by comparing the distributions of conditional odds ratios (one-sided MWU-test).

Predicting associations of schizophrenia genes (Fig. 4a)

Relationship scores between GWAS traits were used to define traits related to SCZ in a tissue-specific manner. In detail, the trait-trait relationship scores (Fig. 3) were z-scored across tissues and for each tissue expressed as a matrix containing z-scored relationships between pairs of traits. The tissue-specific SCZ-related traits were then the top 25 traits that had the smallest Manhattan distance to SCZ in the matrix of z-scores. Analogously, the relationship scores between GWAS traits and cellular components were used to define cellular components related to SCZ. Here, the trait-component relationship scores (Fig. 3) were z-scored across tissues and for each tissue expressed as a matrix containing z-scored relationships between traits and cellular components. The tissue-specific SCZ-related components were then the top 25 cellular components having the highest z-score with SCZ in a given tissue. Potential interactions were then defined as the protein pairs that had one gene confidently associated with SCZ through GWAS (L2G scores > 0.5; n=369) and one gene from the tissue-specific SCZ-related traits and compartments. The tissue-specific networks of associations for SCZ-related genes were then defined as these potential interactions that had association scores exceeding the 97-th percentile for a given tissue (Table S8).

Associations enriched with schizophrenia-related genes (Fig. 4b)

The 369 genes confidently associated with SCZ through GWAS were removed from the collection of proteins in networks of associations for SCZ-related genes for each tissue. Enrichment of other SCZ-related genes was then tested with a one-sided Fisher exact test using all other proteins of the associations for each tissue as background. For these tests, the SCZ-related genes were defined as SCZ drug targets (ChEMBL clinical stage II and higher), genes associated with SCZ in mice (IMPC score >= 0.5) or genes with weak evidence supporting them as causal for SCZ (OTAR L2G < 0.5). The collection of all other genes reported in each database were used as background respectively.

Networks of SCZ-related genes validated by pulldown interactions (Fig. 4c)

Brain interactions were collected and pooled from pulldown studies using micro-dissected human brain tissue or human iPSC-derived neurons (Table S9) [9–13]. These pulldown interactions were filtered for bait proteins that were confidently associated with SCZ (OTAR L2G score > 0.5) to select interactions of SCZ-related baits. Similarly, the tissues of the association atlas were filtered for associations that involved the same SCZ-related baits, which ensured that the networks only contained associations that could have been pulled down with the available baits. Enrichment was then tested with a one-sided Fisher exact test. Background for the pulldown interactions were all associations for SCZ-related baits not found in the pulldowns. Background for the networks of associations for SCZ-related genes were all other quantified associations for the SCZ-related baits in each tissue.

Network of validated interactions for schizophrenia genes (Fig. 4d)

Networks of brain-associations for SCZ-related genes (n=1,694) were filtered for interactions reported in the pulldown studies that involved SCZ bait proteins (n=1,936). The remaining n=205 protein interactions were defined as the validated brain-interactions for SCZ-related genes. These validated interactions were further annotated with the association scores for the other tissues in the association atlas, and whether the interactions were reported in the major protein interaction databases (interactions from HuRI, BioPlex, SIGNOR, Reactome, STRING, CORUM, IntAct (date 2024-05-20) [14] and HuMAP (2020-08-21) [15]). Finally, the proteins for each interaction were annotated with further supporting evidence for being related to SCZ through drug targets (ChEMBL clinical stage II and higher), genes associated with SCZ in mice (IMPC score >= 0.5) or genes with weak evidence supporting them as causal for SCZ (OTAR L2G < 0.5). (Table S10).

Visualizing the network of validated schizophrenia interactions (Fig. 4d)

The validated brain-interactions for SCZ-related genes were filtered for interactions between synaptic proteins (SynGO) to simplify the network for visualization. The remaining interactions were filtered and annotated as follows. The STRING network of physical interactions for human proteins (scores > 750) was filtered for fully connected subgraphs (cliques) of non-bait proteins in the remaining network interactions. Cliques were included if they consisted of at least 3 proteins. The selected cliques were the starting graph of the visualized graph. To this graph, all validated interactions between the cliques and SCZ-bait proteins were added, together with all

validated interactions of these SCZ-bait proteins with other synaptic proteins that had prior evidence for being related to SCZ (i.e., SCZ drug targets (ChEMBL clinical stage II or higher), genes associated with SCZ in mice (IMPC scores >= 0.5), or genes with weaker evidence supporting them as causal for SCZ (OTAR L2G < 0.5)) (c.f., Table S10). The validated interactions were checked for being confidently reported in STRING (scores > 750), IntAct, HuMAP and BioPlex (scores > 0.5 for each), and for being reported in HuRI, Signor, Reactome and CORUM. Validated interactions were annotated as known if they were confidently reported as physical associations in STRING (scores > 750; limiting the annotations to STRING to be consistent with the visualization of cliques). Finally, each clique in the visualized network was tested for being enriched with genes of cellular components through a one-sided Fisher exact test. All other proteins for associations in the brain and all other genes reported for the cellular components were used as backgrounds. Benjamini-Hochberg adjusted p-values were log-transformed and sorted for the cellular component with the strongest enrichment. Cellular components are shown for adjusted p-values < 10e-5. Conditional odds ratios for the shown components all exceeded 300.

Interactions involving synaptic proteins enriched for more probable associations (Fig. 5c)

Sets of synaptic proteins were defined as reported in the SynGO database [7] (release date = 2023-12-01; 1,601 genes), reported as synapse enriched in mouse brains [16] (converted to human proteins through orthologs - 1,733 genes), reported as part of synaptic cellular components (genes in GO cellular components [8] whose name contain descriptions of ‘myelin’, ‘synap’, ‘axo’ (not ‘axonem’), ‘neuro’, ‘dendrit’, or ‘node of Ranvier’ or ‘calyx of Held’ - 2,354 genes), or reported as having elevated expression in the brain (Protein Atlas, maximum expression of the brain tissues exceeds 5x the average expression of the other tissues - 2,719 genes; [17]) (Table S25). The distribution of association scores for each of the tissues in the association atlas were then compared for interactions between synaptic proteins (as defined by the sets above) or interactions involving at least one non-synaptic protein. Here, interactions were defined as protein pairs whose probability exceeded 0.8 in the synaptic interactome. Distributions were compared with a one-sided MWU-test and through the likelihood ratio of the median interaction probabilities.

Defining network of interactions between synaptic proteins (Fig. 5d)

The interaction probabilities of the synaptic interactome and the association scores of the brain from the association atlas were filtered for protein pairs that were quantified in both (1,309,767 protein pairs). The resulting protein pairs were further filtered for pairs of synaptic proteins (397,386 protein pairs; both either reported in SynGO or as synapse-enriched in mouse brains). Finally, the remaining protein pairs were filtered for being both likely co-abundant in the brain (scores > 0.5) and likely co-fractionating in synaptosomes (scores > 0.5), yielding 37,318 pairs of validated interactions between synaptic proteins. These validated interactions were annotated with association scores in the other tissues, and for being reported in the major protein interaction databases (interactions from HuRI, BioPlex, SIGNOR, Reactome, STRING, CORUM, IntAct and HuMAP) (Table S13).

Annotating validated synaptic interactions for brain disorders (Fig. 5d)

Traits were selected for having at least 50 associated genes. Among those, GWAS traits were selected for having associated mouse phenotype data (IMPC scores >= 0.5) or drug targets (ChEMBL clinical stage II or higher). For each tissue in the association atlas, the trait-level association scores for each trait (Fig. 3) were normalized by the median association score in the tissue. The remaining traits were then filtered for having relative trait-level association scores that are elevated in the brain compared to other tissues (z-score > 1 - 10 out of 13 traits were specific to the brain). For each of these traits, the network of validated synaptic interactions was filtered for the interactions between proteins having prior evidence for being related to the trait (i.e., through GWAS (any score), mouse phenotyping (IMPC scores >= 0.5), or drug targets (ChEMBL clinical stage II or higher)). Additionally, the remaining validated synaptic interactions between trait-related genes were filtered for confident interactions (association score > 0.8 in the brain and/or interaction probability > 0.8 in the synaptic interactome). The resulting network consisted of 727 confident validated synaptic interactions between trait-related genes (Table S14). For each brain-elevated trait, the visualized network was limited to interactions involving at least one protein having stronger prior evidence for being related to the trait (i.e., not having GWAS L2G scores < 0.5 - to avoid clusters of genes having weak evidence for a trait). Additionally, to reduce the size of the visualized networks, the networks were further limited to the top 40 interactions that had the highest minimum interaction scores. To these networks, for completeness, the confident validated synaptic interactions were added between their proteins that had weaker prior evidence for the trait (GWAS L2G scores < 0.5 - these proteins were annotated with their variants if the L2G scores exceeded 0.1). Shown are the networks consisting of more than 10 interactions (schizophrenia not shown). Finally, the validated interactions were annotated for being confidently reported in STRING (scores > 750), IntAct, HuMAP and BioPlex (scores > 0.5 for each), and for being reported in HuRI, Signor, Reactome and CORUM.

SUPPLEMENTARY FIGURES

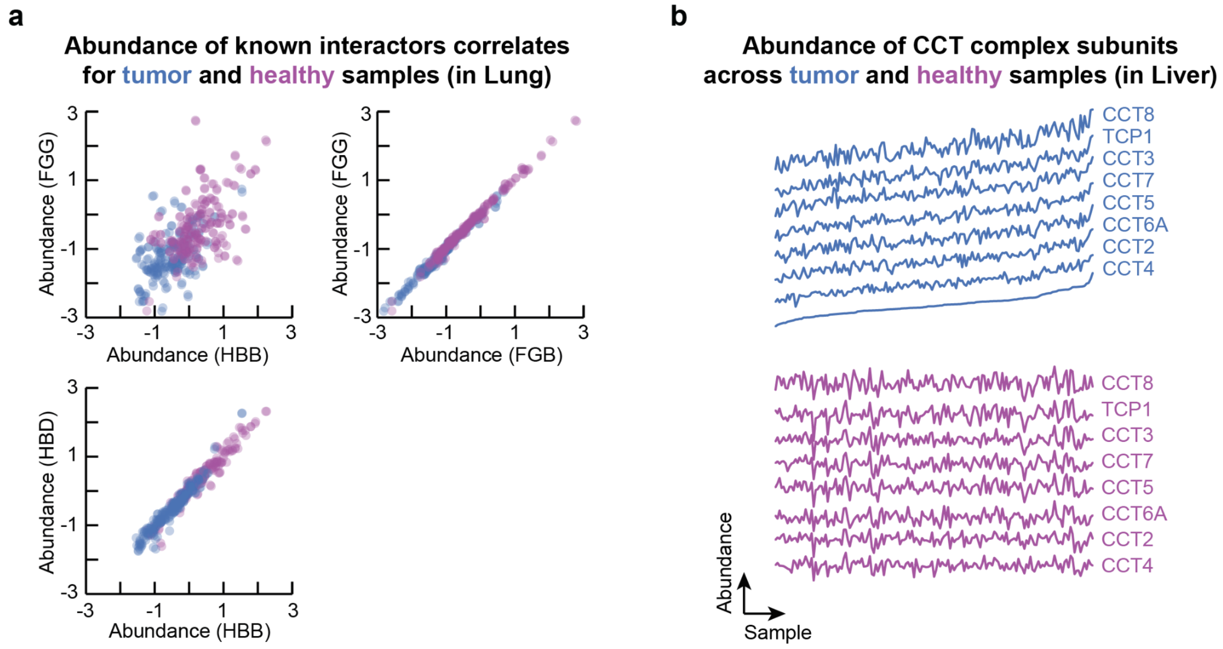

Figure S1. Abundance of known interactors correlates across tumor and healthy samples (related to Fig. 1). (a) To compare the abundance of proteins across samples, we filtered for studies for which we had both tumor and healthy samples whose abundance values were not truncated (17 studies). We further filtered each study to only consider paired samples and genes that were quantified in all samples for both the tumor and healthy samples. As an example of the co-abundance of known protein interactions, we looked at the protein pairs that were most co-abundant in tumors on average across studies. Specifically, we took the Fibrinogen proteins FGB and FGG (average Pearson correlation 0.98) and the Hemoglobin proteins HBB and HBD (0.94). Shown is the (co-)abundance for these proteins in healthy and tumor samples for a representative cohort with many samples (n=216 - in the lung). Each dot represents the abundance in one sample for the healthy (purple) and tumor (blue) samples. **(b)** As an example of the co-abundance of subunits in a protein complex, we selected the abundance of the CCT complex subunits from a representative cohort having a large number paired tumor and healthy samples (n=159 - in the liver). Shown is the abundance of the CCT subunits across patients for the tumor (blue - top) and paired healthy samples (purple - bottom). Patients were sorted by the abundance in tumors of the subunit that had the largest variance across patients. Subunits were sorted by the correlation across tumors with the same subunit (CCT4). Healthy data is shown in the same order. For this example, we found that the abundance of CCT subunits correlates across tumor samples (Pearson correlation coefficient 0.93 ± 0.00 (mean with s.e.m. of n=28 pairs of subunits)) and healthy samples (0.75 ± 0.01). Surprisingly, we found that the abundance of CCT subunits poorly correlated between the healthy and tumor dissections across patients (0.12 ± 0.04), which generalized to the correlation of protein abundance of paired healthy and tumor samples across studies (Pearson correlation 0.16 ± 0.03 for abundance between healthy and tumor samples (across n=17 studies)).

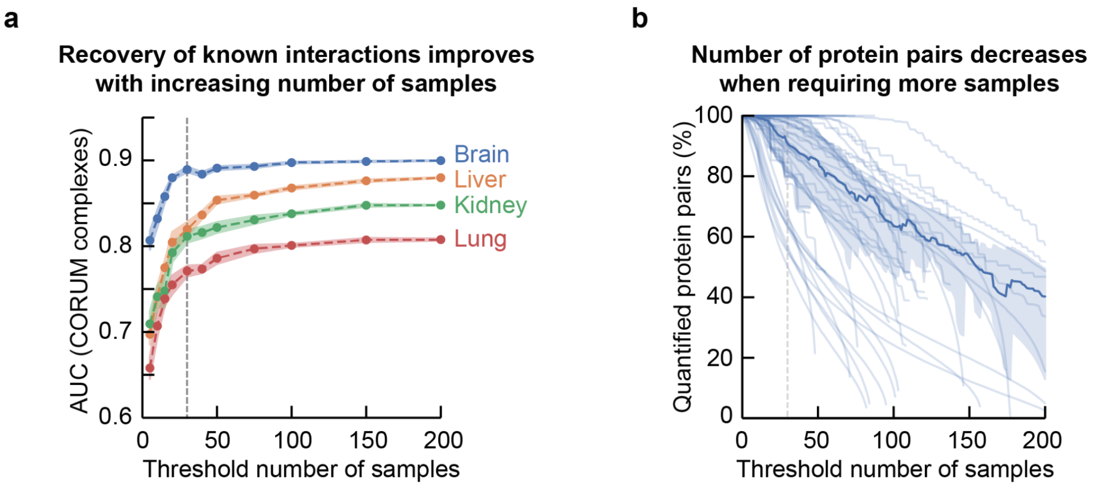

Figure S2. Co-abundance estimates as function of the number of patients per cohort (related to Fig. 1). (a) To test the effect of the available number of samples on the co-abundance estimates, we computed the recovery of known protein interactions when varying the number of samples. Specifically, we computed the AUC for the recovery of interactions between subunits of the CORUM complexes when subsampling the samples of cohorts with large numbers of samples. As cohorts, we selected studies that had cohorts consisting of at least 200 cancer patients and whose AUC on the entire dataset exceeded 0.75. Moreover, we used at most one cohort per tissue, selecting the cohort achieving the highest AUC value on the entire dataset, leaving 4 cohorts (for the kidney, liver, lung and brain). We then filtered each cohort for proteins that were quantified for all samples, such that we could quantify co-abundance estimates for the same protein pairs across sample sizes (i.e., the analysis does not depend on the available protein pairs). We subsampled varying numbers of patients of each cohort and computed the AUC for the recovery of interactions for CORUM complexes using the association probabilities computed from the co-abundance of proteins within the respective subsampled sets of patients. Shown is the AUC as a function of the number of samples for each of the cohorts. As expected, the AUC value decreases as the number of samples decreases. Dotted lines show the mean, shaded area shows the s.em. (n=10 replicate subsamples). **(b)** Additionally, we computed the number of protein pairs that were both quantified in at least a threshold number of samples as a function of that threshold number. Here, we did not filter for proteins quantified for all samples. Shown are the quantified protein pairs (a percentage of all possible pairs) as a function of the threshold number of samples required for computing co-abundance estimates for all cohorts (light blue lines (n=50 cohorts)) and the median (dark blue line) with the interquartile range (shaded area). The percentage of protein pairs for which we can compute a co-abundance estimate decreases when requiring more samples (increasing threshold number of samples). In (a-b), the gray dotted line shows the threshold number of samples (n=30) used throughout this work. Overall, these analyses demonstrate that having more samples for computing co-abundance estimates result in association scores for less protein pairs but higher AUC values. As a compromise, we used a threshold of 30 samples for computing co-abundance estimates throughout all analysis.

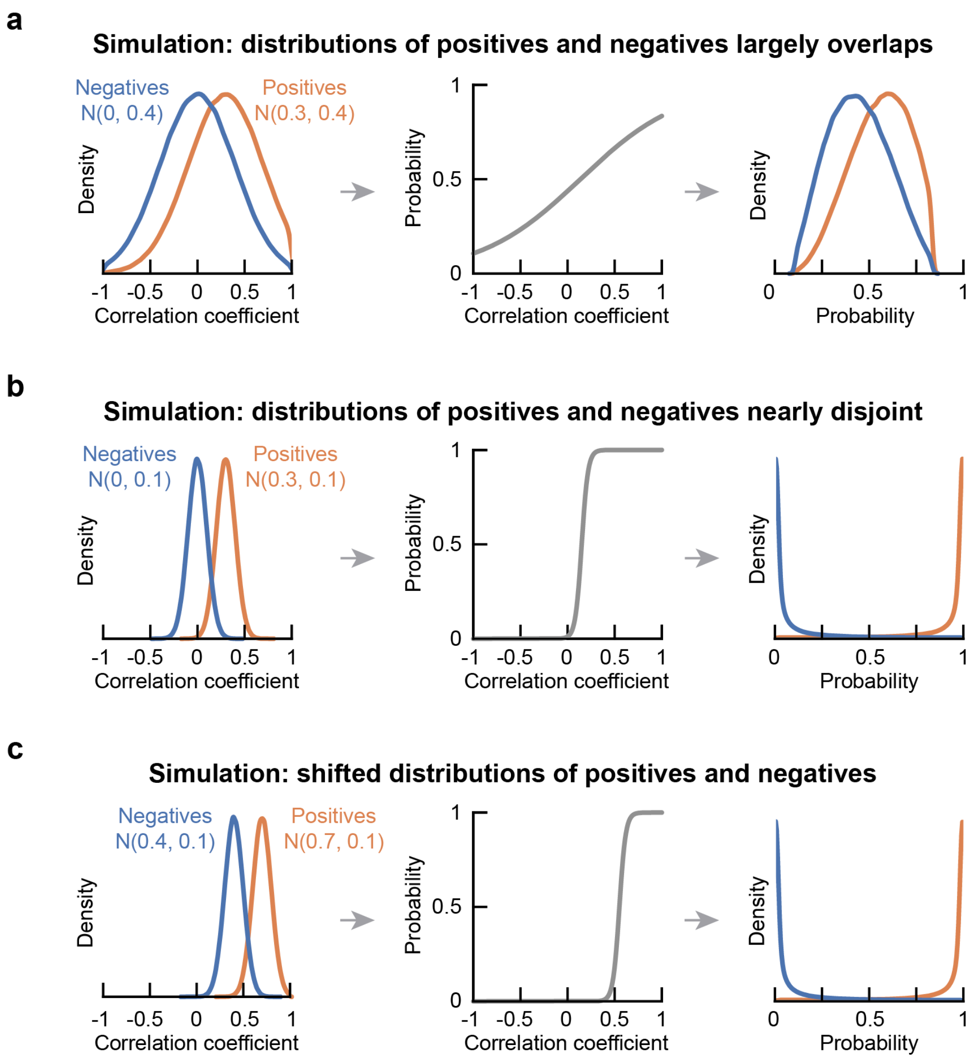

Figure S3. Using a logistic model for converting co-abundance estimates to probability scores (related to Fig. 1). Correlation coefficients estimate the co-abundance of proteins across samples (Fig. S1). We applied a logistic model with ground-truth positives to estimate a likelihood of protein-protein associations from these correlation coefficients. Shown are examples of computing probability scores for simulated distributions of co-abundance estimates (correlation coefficients) for samples labeled as positives and negatives. **(a)** We simulated correlation coefficients for negative and positive samples by sampling from the normal distribution (mean 0 (negatives) and 0.3 (positives), standard deviation 0.4 for both; 1 million samples for each class; Samples were filtered for being within the range of possible correlation values). We then fitted a logistic regression with two free parameters (no penalty parameter) to the simulated correlation values. Finally, the fitted logistic model was used to transform the simulated correlation values to probabilities. Shown is the distribution of correlation coefficients for positives (orange) and negatives (blue) (left plot), the logistic model fitted to the data (middle plot), and the correlation values mapped to probability scores for all samples (right plot). **(b)** identical to (a) but for sampling narrower distributions (standard deviation 0.1 for both positives and negatives). **(c)** identical to (b) but for sampling distributions of correlation coefficients that are shifted (mean 0.4 (negatives) and 0.7 (positives)). For the cohorts, we defined the set of ground-truth positives as all protein interactions reported in CORUM [6]. Specifically, for each protein complex, the positives were defined as all possible pairs of subunits. For each cohort, the space of negatives was then defined as all protein pairs for which association scores were quantified for a given dataset (e.g., cohort, tissue, etc) but that were not ground-truth positives (in CORUM) (also see Methods). This choice is motivated by the scale of the negative

space, a lack of (sufficiently many) ground-truth negatives for protein interactions, and to avoid biases in potential selected subsets of negatives (e.g., when filtering for protein pairs that are located in unconnected cellular compartments). The resulting negative space contains (some number of) true positives (true interactions that are not described in CORUM). This bias causes the recovery of ground-truth positives to underestimate the true recovery of positives, as known interactions are more co-abundant than arbitrary other protein pairs that are not known interactors [18–21]. The motivation for using a logistic model to estimate probabilities is as follows. The logistic (log-linear) model is a rank-preserving transformation, such that the ranking of protein pairs is preserved when converting correlation estimates to association probabilities. Moreover, the logistic model - as part of the exponential family - makes the fewest assumptions on the correlation coefficients for computing association probabilities. Finally, using a single ground-truth set of positives ensures that association probabilities are interpretable and calibrated between studies, allowing for a straightforward combination of different studies for a single tissue and comparisons between studies - essential for scoring the tissue-specificity of associations. A separate logistic model was fitted for each study, using its correlation estimates and the defined sets of ground-truth positives and negatives. The purpose of fitting a separate model for each study is to correct for batch effects - biases in correlation estimates and differences in the accuracy for recovering of known interactions - by rescaling correlations to probabilities using the same set of ground-truth positives. Specifically, for each study, the model's probabilities reflect the confidence in the likelihood of protein associations through the spread of probabilities around the midpoint of the logistic curve. Indeed, probabilities are more centered for less confident predictions, reflecting the confidence in the ability to recover known protein interactions - compare (a) and (b); the overlap in distributions of correlation coefficients dictates the spread of probabilities around the midpoint of the logistic curve. Simultaneously, the model adjusts cohort-specific biases in the correlation coefficients. For example, protein pairs can be generally positively correlated according to one study compared to other studies - compare (b) and (c); a shift (bias) in distributions of correlation coefficients yields identical probability distributions. Thus, a separate logistic model for each study, using a single set of ground-truth positives, transforms the correlation estimates to adjust for cohort-specific biases and to reflect the confidence in the cohort's ability to recover known protein interactions.

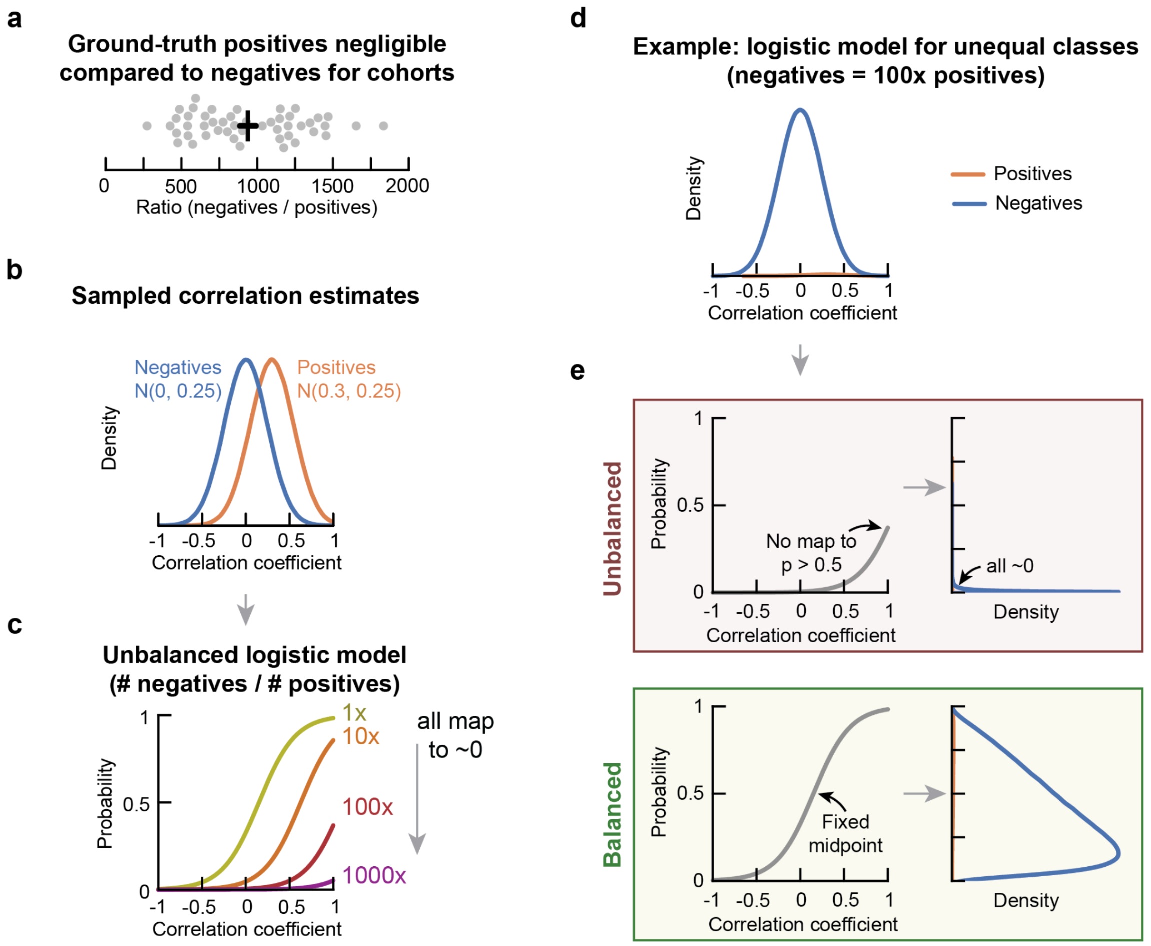

Figure S4. Balancing the number of positives (known interactions) and negatives for the logistic model when transforming co-abundance estimates to probabilities (related to Fig. 1). Illustrative simulation motivating the 'class balancing' of ground-truth positives and negatives when transforming correlation coefficients to probabilities through a logistic model. **(a)** Ratio of the number of negatives and ground-truth positives for the protein pairs having correlation coefficients in the tumor cohorts. Ground-truth positives are defined as all pairs of subunits for protein complexes in CORUM, with negatives being all protein pairs that are not ground-truth positives (also see Fig. S3 and Methods). Each dot represents one cohort. Error bar shows mean with s.e.m. (n=48 cohorts). Negatives outnumber ground-truth positives almost 1000-fold (average 938.7 across cohorts). **(b)** To study the effect of differences in the number of positives and negatives, we simulated correlation coefficients for positive and negative samples by sampling from the normal distribution (mean 0 (negatives) and 0.3 (positives), standard deviation 0.25 for both; 1 million samples for each class). Shown is the distribution of correlation coefficients for positives (orange) and negatives (blue). **(c)** We fitted an unbalanced logistic model with two free parameters (with intercept, no penalty) to the simulated data from (b). Here, unbalanced means that all protein pairs have equal weight in the loss function when fitting the model. Shown is the fitted logistic curve when the negatives outnumber the positives by 1000-fold (purple curve), 100-fold (red), 10-fold (orange), or equal numbers (yellow). The unbalanced logistic model maps correlation coefficients continuously closer to probability zero as the fold-difference between the number of negatives and positives increases, yielding hard to interpret scores. We aim to avoid this effect through "class balancing" the number of ground-truth positives and negatives. Specifically, when fitting the logistic model, we re-weighted the protein pairs inversely proportional to the number of samples in its class (positives or negatives), such that the total weight of the positive samples is equal to the total weight of the negative samples (see methods for mathematical details). **(d)** As an example for fitting a logistic model for balanced classes, we simulated correlation coefficients for the case where negatives outnumber positives by 100-fold. Distributions are identical to (a). Shown are the distributions of simulated positives (orange) and negatives (blue). **(e)** We

fitted unbalanced and balanced logistic models to the simulated correlation coefficients from (d). As expected, we found that the unbalanced model maps the co-abundance estimates to probabilities close to zero, with the logistic curve not permitting probabilities that exceed 0.5 (as in (c) - red box). In contrast, the logistic model that balanced classes (re-weighted the positives and negatives to the same total weight) has a midpoint that is fixed and independent of the ratio between positive and negative samples (green box). The model maps co-abundance estimates to probabilities as if the number of positives and negatives were equal (compare the logistic curve with the yellow curve in (c)). We used logistic models that balances the weights of positives and negatives when converting proteomics-derived co-abundance estimates to probabilities.

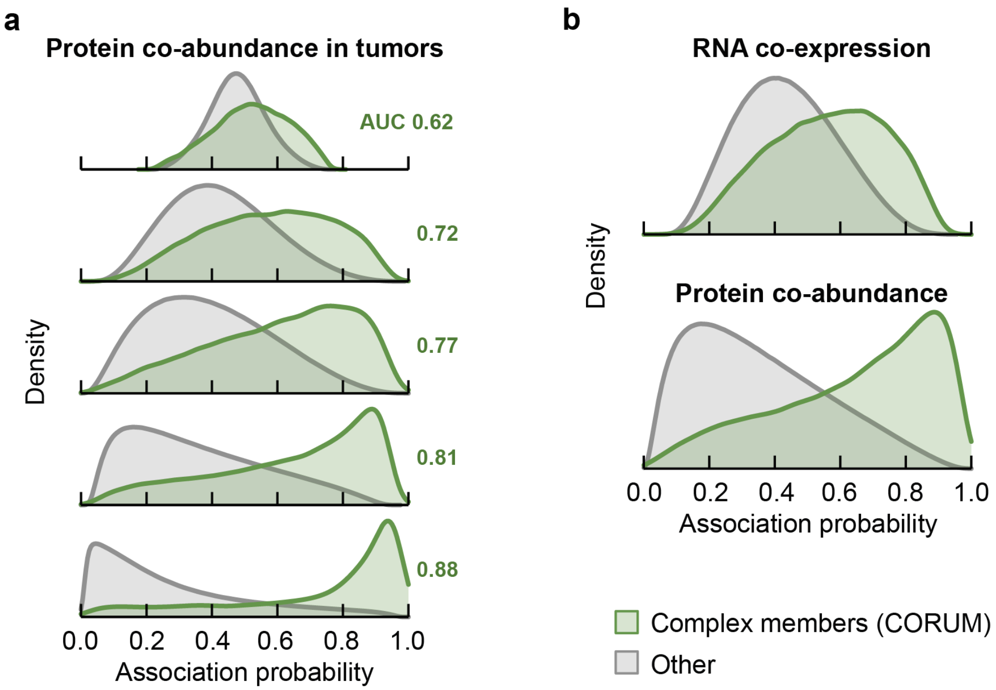

Figure S5. Associations between complex members are more likely than associations of other protein pairs (related to Fig. 1). We compared the association probabilities of ground-truth positive interactions (protein pairs that are reported as complex members in CORUM) with the association probabilities of other protein pairs. **(a)** Association probabilities derived from protein co-abundance in tumor samples for five different representative cohorts used throughout the analysis. Shown is the density of protein pairs across probabilities (KDE density estimates) for complex members (green) or for other protein pairs (gray). Cohorts are sorted from top to bottom by AUC values for recovering the protein complex members (AUC values in green). Probability distributions are more centered for lower AUCs and more skewed for higher AUCs (also see Fig. S3). Using the cohorts (Fig. 1a), we found AUCs of 0.79 ± 0.01 (mean with s.e.m. for n=48 cohorts) for the tumor-derived probabilities and 0.72 ± 0.01 (n=23) for the healthy-derived probabilities. **(b)** As in (a) but comparing association probabilities derived from RNA co-expression or protein co-abundance of the same samples. Together, these distributions demonstrate that the associations between complex members are more likely compared to the associations of other proteins, especially as quantified through protein co-abundance compared to RNA co-expression. In (a-b), the density curves are not proportional to the number of protein pairs available for each class.

Association scores not driven by cell-type composition of tissue samples

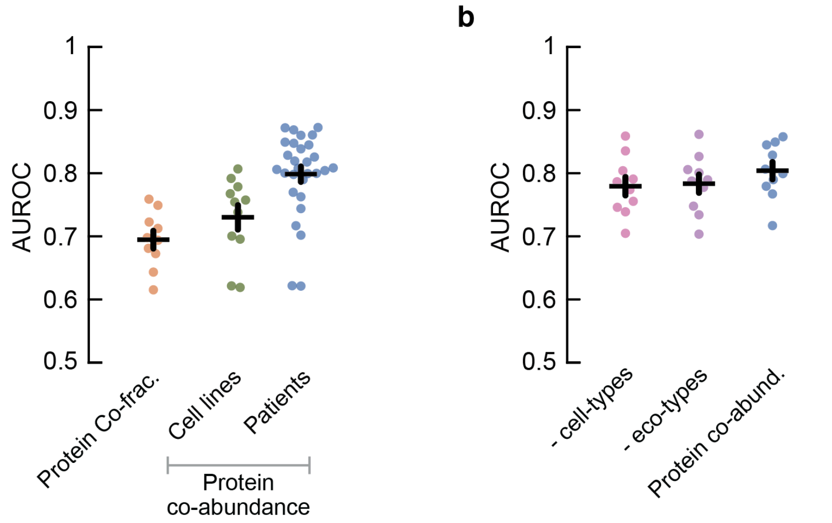

Figure S6. Association scores not driven by cell-type composition of tissues (related to Fig. 1). Differences in cell-type composition and variations in their relative proportions between samples can confound the co-abundance of proteins across tissue samples. We study the effect of cell-type composition on the recovery of protein interactions in a quantitative manner through two different approaches. **(a)** As a first approach, we collected and compiled 11 proteomics datasets consisting of samples from populations of cells of the same cell type [19,20,22–27]. Association scores for these 11 sets of cell types were computed following the identical methodology as for biopsies of cancer patients (Fig. 1; Methods). Shown are the AUC values for the recovery of protein interactions (CORUM) for the cell lines (green), together with the AUC values for protein co-fractionation (orange) and protein co-abundance (blue) reproduced from Fig. 1c. Each dot represents one dataset. Error bars show mean with s.e.m.. We found that the co-abundance in cells (AUC 0.73 ± 0.02 (n=11)) typically outperformed co-fractionation data (AUC 0.69 ± 0.01 (n=10); p-value = 0.08; one-sided Welch's t-test) but not co-abundance in tissues (AUC 0.80 ± 0.01 (n=29); p-value = 3.8e-3) for recovering known interactions, suggesting that cell-type composition cannot be the sole driver of protein co-abundance across tissue samples. **(b)** As a second approach, we used EcoTyper [28] to decompose the cell-type and eco-type composition of tumor samples based on paired RNA-sequencing data. Specifically, we used EcoTyper to specify the ecotypes and cell type composition for the 10 cohorts that contained carcinoma samples, had RNA-sequencing data available and were cancer types supported by EcoTyper. We then regressed the output of EcoTyper - cell-types and ecotypes per sample - out of the proteomics data to subtract the fraction of the protein abundances that can be explained by the cell-types or eco-types of the samples. Specifically, per protein j, we fitted a linear model $A_j \sim \beta_0 + \sum_{i=1}^{n} \beta_i \cdot T_i$ to the abundances A_j of the protein across samples, using the total abundances of the cell-types or ecotypes $\{T_i\}_{i \in (1,n)}$ across samples, and with parameters $\{\beta_i\}_{i \in (1,n)}$ and intercept β_0. We then subtracted the model's prediction ($A_{j,pred} = \beta_0 + \sum_{i=1}^{n} \beta_i \cdot T_i$) from the protein abundances for all samples. Note that the use of an intercept does not affect co-abundance estimates (also see Methods). Datasets were then filtered for having at least 3-fold more samples compared to the number of eco-types or cell-types to ensure that abundances are not fully explained by simply regressing out sufficiently many linearly independent variates. Finally, we computed protein association scores as before (Methods). Shown are the AUC values for the recovery of protein interactions (CORUM) with the association scores when cell-type composition (pink) or eco-types (purple) are regressed out, compared to the association scores from the same cohorts and abundances without regressing out the ecotypes or cell types (blue). Each dot represents one dataset. Error bars show mean with s.e.m. (n=10 cohorts). We found that the association scores when regressing out the eco-types (AUC 0.78 ± 0.01) or the cell-type composition (0.78 ± 0.01) did not considerably change the recovery of protein associations compared to the association scores derived from the same cohorts and abundances without

regressing out the ecotypes or cell types (0.80 ± 0.01; p-values 0.16 and 0.12 respectively; one-sided Welch's t-tests). These observations suggest that the cell-type and ecotype composition of tissue samples - as quantified through gene expression - have a limited effect on the co-abundance of proteins across samples. Overall, both the cell-type derived co-abundance and the RNA-sequencing based cell-type decomposition demonstrate that cell-type composition plays a minor role in driving protein co-abundance across tissue samples.

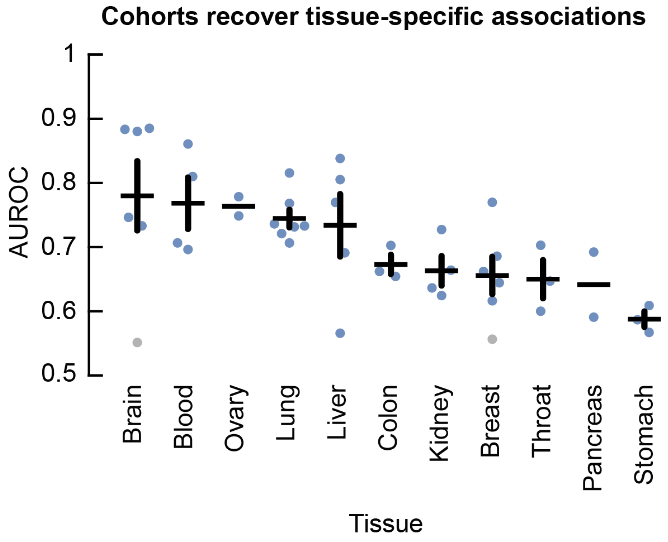

Figure S7. Cohorts recover tissue-specific associations (related to Fig. 1). We tested whether a cohort of a tissue could recover associations predicted by the other replicate cohorts of the same tissue. For this, we used the tissue-specific associations for each tissue: associations whose average probability exceeded the 95-th percentile for a given tissue and whose average probability remained below 0.5 across all other tissues. We then used a hold-one-out methodology where we predicted the tissue-specific associations for a given tissue with all-but-one cohorts of that tissue, and tested how well the withheld cohort could recover the predicted associations of its tissue. By predicting and recovering the tissue-specific associations through sequentially withholding each cohort of a given tissue, we could score how well each cohort aligned with the other cohorts of that tissue. Shown are the AUCs for recovering the tissue-specific associations across tissues. Each dot represents one cohort. Error bars show mean and s.e.m. (when available; showing the brain (n=6 cohorts), blood (n=4), ovary (n=2), lung (n=7), liver (n=5), colon (n=3), kidney (n=4), breast (n=6), throat (n=3), pancreas (n=2), and stomach (n=3)). We excluded cohorts for further analysis when the AUC was below 0.6 and at least 5 other cohorts of the same tissue were available. We chose not to incorporate these cohorts for computing the aggregated association scores for tissues as the tissue-specific associations of these cohorts were poorly aligned with the other (sufficiently many) cohorts of the same tissue. We found two such cohorts (for the brain and breast respectively - colored gray).

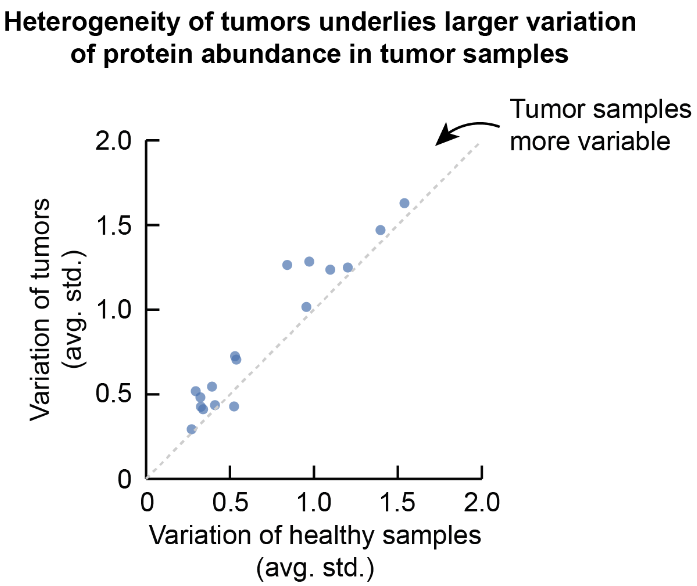

Figure S8. Comparing variation of abundance for tumor and healthy samples (related to Fig. 2). To compare the variation between samples of tumor and healthy samples, we selected the abundance of common genes and common samples in the n=17 studies that had at least 30 paired tumor and healthy samples available. We then computed the standard deviation of abundances across samples, averaged over all genes. We found that the standard deviation across patients was 0.83 ± 0.1 (mean with s.e.m. across cohorts) for the tumor samples and 0.70 ± 0.1 for the paired healthy samples. Shown is the variation across tumor samples as function as the variation across healthy samples. We compared the distributions of average standard deviations using a paired t-test (one-sided; p-value 2.3e-4 (n=17)), suggesting that - averaged over all genes - there is no reason to assume that the tumor samples are less variable than the paired healthy samples. Finally, when comparing the standard deviation of abundances across samples between tumor and healthy samples per gene, we found that the tumor samples were 1.34 ± 0.06 -fold more variable compared to the healthy samples. Overall, these analyses suggest that the genetic heterogeneity of tumors is represented in the data by a larger variation in abundance across the tumor samples compared to the paired healthy samples in the cohorts. Dots represent the different studies. Diagonal indicates equal variation between tumor and healthy samples.

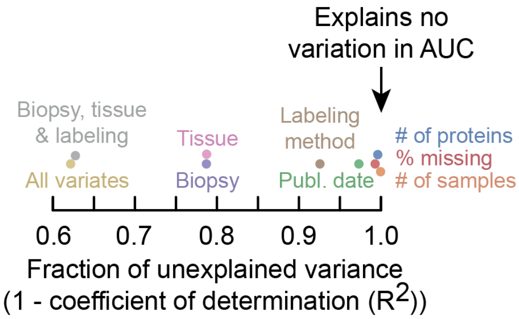

Figure S9. Factors partially driving the variation in AUC values between studies (related to Fig. 2). Several factors may affect the variability in accuracy between scores for recovering protein interactions, as observed both at the tissue-level (Fig. 2b) and for cohorts of different tissues (Fig. S7). To quantify some of such factors, we specified potentially confounding factors that may affect the recovery of known protein interactions for each cohort, such as factors relating to the quality of MS data (the use of isobaric labeling, the year of the study, number of detected proteins, missingness of data) and the available biological material (healthy- or tumor-derived biopsies, tissue of origin, number of samples). We then computed the correlation coefficients between the AUCs and individual variates. Additionally, we one-hot encoded the categorical variables and fitted a linear model to the AUC values (from CORUM) for all cohorts (n=71, tumor + healthy cohorts) using different subsets of variates. We required positive regression coefficients when fitting the model to categorical variables or combinations of different variates to ensure that the explained variation of AUCs was additive. Finally, we expressed the explained variation in AUCs using the fraction of variance unexplained (FVU; 1 - coefficient of determination (R^2)). Each dot shows the FVU for a linear model predicting the AUC of cohorts using the labeled variates. We found that most variance in AUCs remains unexplained after accounting for all variates (FVU = 62.2%; yellow), suggesting that there are likely other, unexplored sources of variation for the AUCs. Surprisingly, we also found that the available number of samples poorly correlates with the AUCs (ρ = 0.02), and indeed does not explain any variance in AUCs (FVU = 99.9%; orange). However, the number of available samples is likely still an important variate driving the recovery of protein interactions, given that the AUCs decrease when decreasing the number of samples at the level of individual cohorts (Fig. S2). We found that the variation in AUCs can be partially explained by the use of isobaric labeling of samples for MS (ρ = 0.27; FVU = 92.6%; brown), the tissue of origin (FVU = 78.7%; pink - unlikely due to cell-type composition; see Fig. S6), and whether the association scores are derived from biopsies of healthy or tumor tissue (ρ = 0.46; FVU = 78.7%; purple - tumor biopsies have larger variation in abundance across samples, leading to better co-abundance estimates; see Fig. S8). Together, these three factors cover almost all variance that is explained by our linear model (FVU = 62.7%). Following the same methodology, we found that the variability in AUCs between tissues can be largely explained by the number of cohorts available for a given tissue (ρ = 0.67; FVU = 54.5%). Overall, these observations suggest that the number of cohorts per tissue, the number of samples per cohort (Fig. S2), the biopsy (healthy or tumor tissue), the tissue of origin and the mass spectrometry methodology are primary drivers of the variability in AUCs between scores.

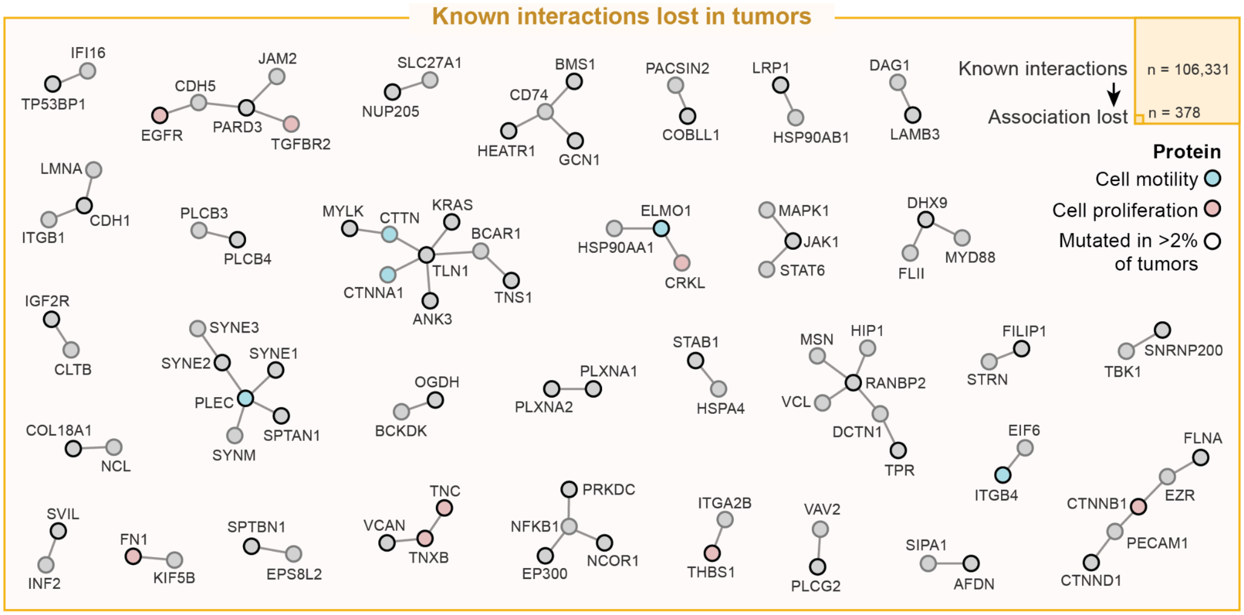

Figure S10. Network of protein interactions that are lost in tumors (related to Fig. 2). For six tissues we had protein association scores derived from both tumor and from healthy biopsies (colon, kidney, liver, lung, stomach, throat). We hypothesized that there may be some protein interactions that are lost for tumors and that the loss of these interactions could be reflected in our association scores. To test this, we computed the difference between the healthy and tumor-derived association scores for each of the six tissues. We then filtered for protein pairs that are known to interact (reported in STRING with scores exceeding 400 [5]). Finally, for each tissue, we selected the protein pairs for which the tumor-derived association score was at least 0.5 below the association score derived from the healthy biopsies of the same tissue ("lost associations" – 392 protein pairs). Thus, we ensured that we only considered known interactions whose likelihood of associating substantially decreases for at least one tissue. We found that the known protein interactions were enriched for lost associations compared to associations without prior evidence (p-value 1.6-17 – one-sided Fisher exact test). Here, we used all non-"lost associations" with and without having prior evidence as backgrounds respectively. Next, we explored the proteins that were involved with the lost associations. First, using mutation frequencies for any tissue from the Pan-cancer Atlas (cbioportal[29], we found that 12% of the proteins with lost associations were frequently mutated in cancers (mutation frequencies exceeding 2%). Looking at the GO biological processes, we found that the lost associations were enriched for proteins related to several respiration-related processes (mitochondria, electron transport chain, oxidative phosphorylation, cellular respiration: p-value 3.6e-15; odds-ratio 36.5; GO:0045333), with the vascular endothelial growth factor receptor signaling pathway (p-value 3.9e-3; odds-ratio 11.7; GO:0048010), positive regulation of cell population proliferation (p-value 4.0e-3; odds-ratio 2.6; GO:0008284), cell motility (p-value 9.9e-3; odds-ratio 6.1; GO:0048870), and positive regulation of ERK1 and ERK2 cascade (p-value 1.8e-2; odds-ratio 3.5; GO:0070374) also being amongst the most related processes (one-sided Fisher exact tests with Benjamini-Hochberg (BH) adjusted p-values; all other proteins from the known interactions and all other proteins in GO biological processes were used as backgrounds respectively). Finally, we found that 0.5% ± 0.3% (mean with s.e.m. (n=6 tissues)) of likely associations had a difference that exceeded 0.5 when comparing the healthy and tumor-derived scores. In other words, at most 0.5% of protein pairs have a substantial difference in association scores and are likely to be associating according to either – but not both – the healthy-derived or the tumor-derived scores. The substantially different association scores are thus negligible on the scale of the association atlas. Therefore, while the known interaction lost in tumors may be sensible in the context of cancer, our observations demonstrate the lack of a sizable cancer-specificity in the association atlas. The visualized network shows the known protein interactions for the frequently mutated genes whose associations are lost in tumors. Nodes are colored for the proteins being associated with the positive regulation of cell population proliferation (red), cell motility (blue) or other (gray). Node edges indicate proteins that are frequently mutated in cancer (black - mutation frequency >2%) or not (gray).

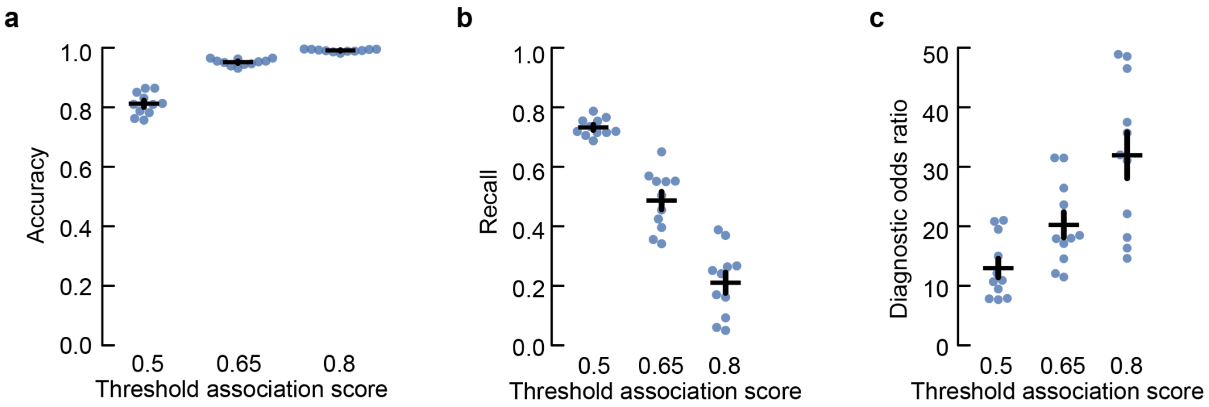

Figure S11. Performance metrics for associations depending on threshold (related to Fig. 2). For each tissue, we quantified the accuracy, recall, precision and diagnostic odds ratio for the threshold association scores for likely (scores > 0.5), confident (scores > 0.8), or other (scores >0.65) associations. Here, we used as ground-truth positives all possible pairs of subunits for protein complexes in CORUM, with the negatives being all other quantified protein pairs for each tissue (Methods). Shown are the accuracy **(a)**, recall **(b)** and diagnostic odds ratio **(c)** for the different score thresholds. Each dot represents one tissue. When using the likely associations (scores > 0.5), we found that the accuracy is 0.81 ± 0.01 (mean with s.e.m. (n=11)), with a recall of 0.73 ± 0.01, and a diagnostic odds ratio of 13.0 ± 1.6 (the odds of being likely when a true interaction, relative to the odds of being likely if not a true interaction). Thus, on average, over 80% of all calls we make are correct, while calling over 70% of all ground-truth positives correctly, and having roughly a 13-fold higher chance of being likely for true interactions compared to non-true interactions. These metrics change as expected for more stringent cut-offs, yielding an accuracy of ~0.99, a recall of 0.21 ± 0.03, and a diagnostic odds ratio of 31.9 ± 3.9 for confident associations (scores > 0.8). Finally, we note that the precision increased from ~0.003 for likely associations to ~0.02 for confident associations as expected for a metric with a high false discovery rate (FDR), given that the negatives outnumber the positives by almost 1000-fold (Fig. S4).

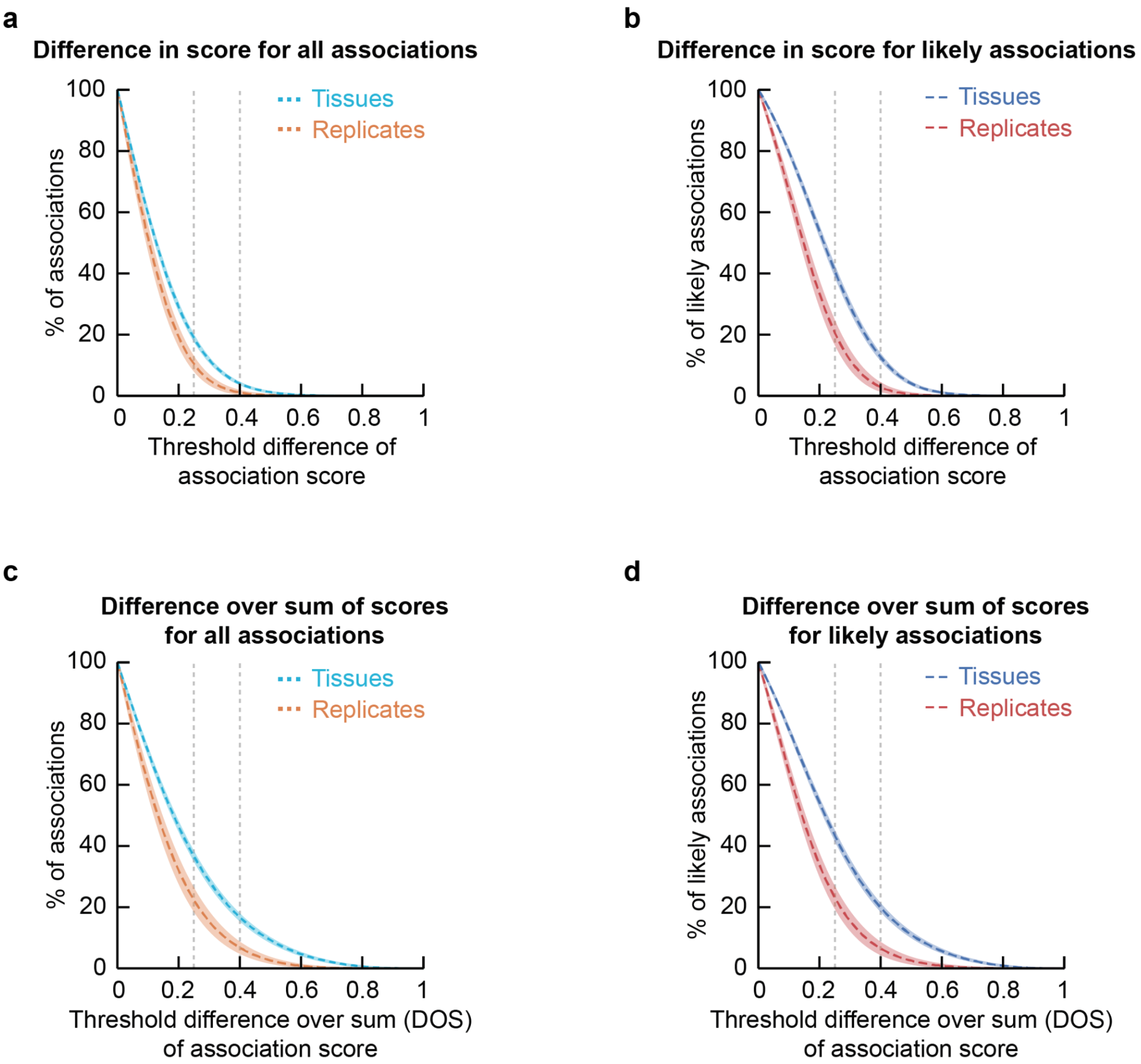

Figure S12. Association scores reproduce well between replicates with larger differences between tissues (related to Fig. 2). (a) For each pair of tissues, we filtered the association scores for protein pairs quantified in both tissues or tumor- and healthy-derived replicates. For each protein pair, we then computed the absolute difference between their association scores. Shown is the percentage of protein pairs whose difference exceeds a given threshold between tissues (blue curve) or between tumor- and healthy-derived replicates (red curve). **(b)** As in (a), but filtering for protein pairs that are quantified in both tissues and whose association is likely (score > 0.5) in at least one of the tissues or replicates. **(c-d)** Following (a-b), but expressing the absolute difference as a difference over the sum (DOS) (i.e., for each protein pair we expressed the absolute difference in association scores relative to the sum of association scores). Shown is the percentage of protein pairs whose DOS exceeds a given threshold for all associations **(c)** or for the likely associations **(d).** In (a-d), colored dotted lines show average percentage across pairs of tissues or replicates, with the shaded area showing the s.e.m. (n=55 pairs of tissues and n=6 replicates). Gray dotted lines indicate the 0.25 and 0.4 threshold scores. As expected, the differences in association scores for tumor- and healthy-derived replicates are smaller than differences between pairs of tissues. Specifically, we found that the differences in scores remained below 0.25 for 81.1% (tissues) and 89.6% (replicates) of all associations, while differences in scores exceeded 0.4 for 4.0% (tissues) and 1.1% (replicates) of all associations (a). In contrast, the differences in scores remained below 0.25 for 59.2% (tissues) and 79.3% (replicates) and exceeded 0.4 for 12.6% (tissues) and 2.8% (replicates) of the likely associations (scores > 0.5) (b). We found analogous results looking at differences in scores relative to the sum of scores. Specifically, we found that the DOS remained below

0.25 for 63.4% (tissues) and 77.6% (replicates) of all associations, and the DOS exceeded 0.4 for 16.7% (tissues) and 6.7% (replicates) of all associations (c). In contrast, the differences in scores remained below 0.25 for 56.6% (tissues) and 76.8% (replicates) and exceeded 0.4 for 19.9% (tissues) and 6.5% (replicates) of the likely associations (scores > 0.5) (d). In other words, following (d), over 75% of likely associations have scores whose difference is less than 25% of their average between pairs of replicates, while less than 10% of associations have scores whose difference is more than 40% of their average. Together, these results suggest that the association scores reproduce well in both absolute (a-b) and relative (c-d) terms, with replicates having smaller differences than pairs of tissues and likely associations being more variable than the other associations.

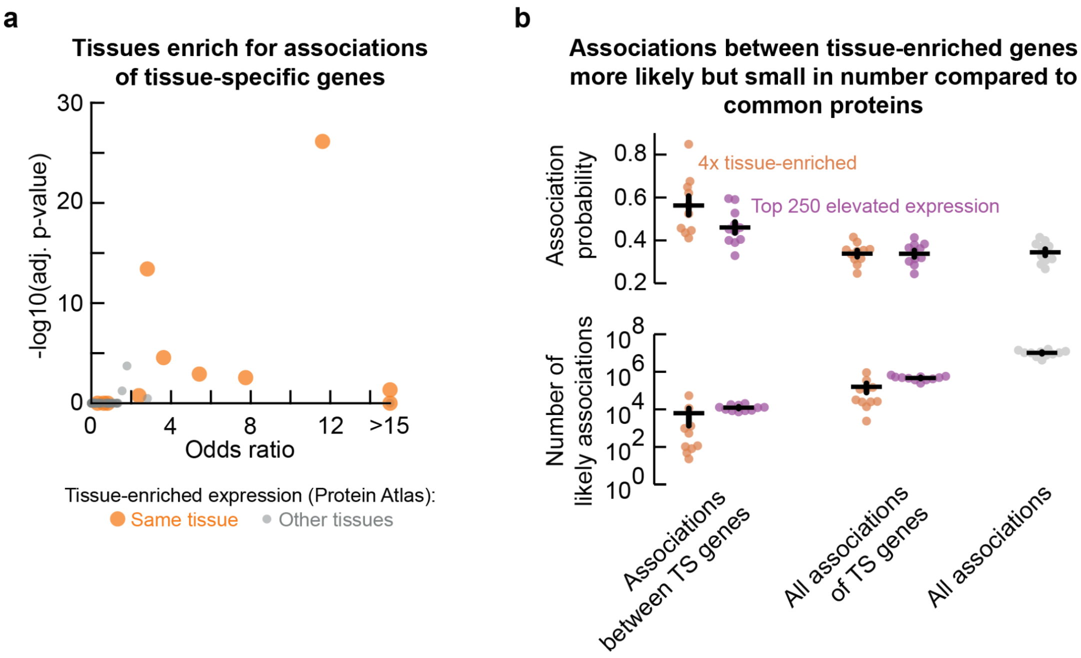

Figure S13. Tissue-specific gene expression does not drive the protein associations for tissues (related to Fig. 2). Quantifying the effect of genes with elevated tissue-expression profiles on the association scores for each tissue in the association atlas. We collected gene expression levels for human tissues from the Protein Atlas (consensus normalized expression) [17], which we then aggregated at the whole-tissue-level by taking the maximum expression for each protein over the sub-tissues for the brain (cerebellum, amygdala, cerebral cortex, basal ganglia, choroid plexus, hippocampal formation, hypothalamus, midbrain, pituitary gland, spinal cord) and colon (colon, duodenum, small intestine). Expression of bone marrow was used for blood. **(a)** Enrichment (odds ratio and log10 Benjamini-Hochberg (BH) adjusted p-value; one-sided Fisher exact test) of genes with tissue-enriched expression amongst the proteins for which we quantified protein associations in each tissue. Here, the tissue-enriched genes have at least 4-fold higher expression when compared to any other tissue (following the definition from the Protein Atlas). Backgrounds were all other genes with expression reported in the Protein Atlas and all other genes quantified in our association atlas. Shown is the enrichment of tissue-enriched genes for the same tissue (orange) and for the other tissues (gray). Infinite odds ratios were limited to 15. We found that the tissues from the association atlas enriched for tissue-specific genes, especially when comparing the enrichment of their tissue-specific genes to the enrichment of genes specific to the other tissues (p-value = 1.3e-6; one-sided MWU-test comparing the odds ratios). **(b)** Association probability and number of likely associations of proteins whose expression is tissue-specific ("TS"). Shown is the median association probability in each tissue for all quantified protein pairs (gray dots (n=11 tissues)), all associations of the tissue-specific proteins, or the associations between the tissue-specific proteins. As tissue-specific proteins, we used the definition of the Protein Atlas ("tissue-enriched"; genes whose expression is at least 4-fold higher in one tissue compared to any other tissue – orange dots (n=10 tissues)) or the top 250 proteins that are quantified in our association atlas and had the most tissue-specific expression ("elevated expression"; highest z-score across tissues – purple dots (n=11 tissues)). Each dot represents one tissue. Given the low and varying number of proteins that were tissue-enriched for some tissues, we used here the top 250 proteins with elevated expression as representative to quantify the effect of gene expression (with the statistics for tissue-enriched genes between brackets). We found that the median association probabilities changed from 0.35 ± 0.01 (mean with s.e.m.) for all quantified protein pairs to 0.34 ± 0.02 (0.34 ± 0.01) for all associations of proteins with tissue-elevated expression, and to 0.46 ± 0.03 (0.56 ± 0.04) for the associations between the proteins with tissue-elevated expression. Here, the likely associations between the proteins with elevated expression represented 0.13% ± 0.02% (0.06% ± 0.05%) of all likely associations, while all likely associations of the proteins with elevated expression represented 4.8% ± 0.2% (1.7% ± 0.8%) of all likely associations of the tissues. Overall, the proteins that were quantified for a given tissue in the association atlas are generally enriched for genes with

elevated expression for that same tissue, but not the other tissues. Indeed, the protein associations between these genes are more likely than the associations of other proteins. However, the associations of proteins with elevated expression are not more likely compared to the associations of all proteins, and the collection of likely associations of proteins with elevated expression is not substantial compared to all likely associations.

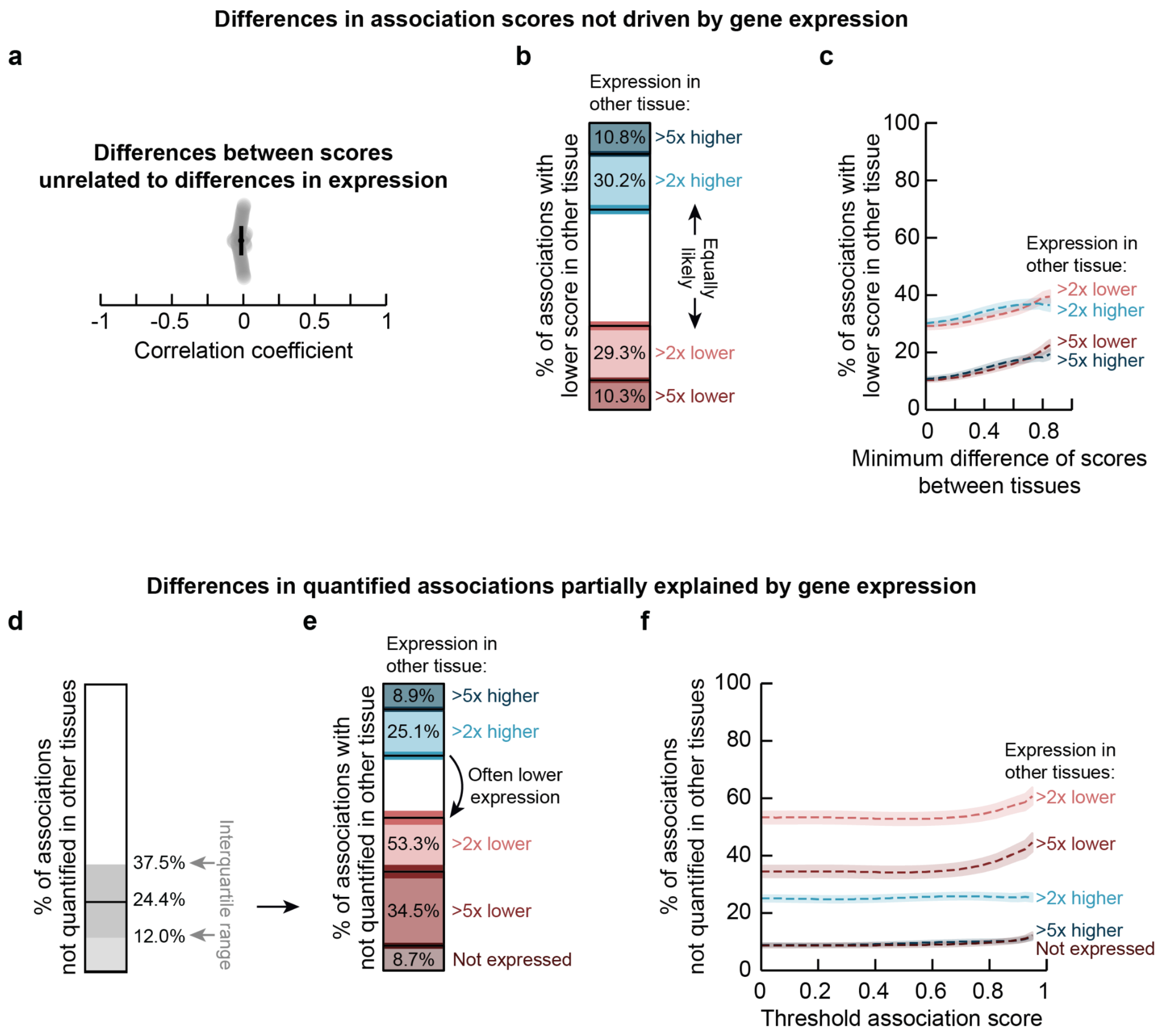

Figure S14. Differences between tissues not driven by differences in gene expression (related to Fig. 2). One of the drivers of differences in protein interactions between tissues is gene expression. Here we quantified the effect of differences in gene expression on differences in protein associations between tissues. First, we consider the associations that were co-quantified between pairs of tissues **(a-c)**, and then a tissue's associations that are not quantified in other tissues **(d-f)**. **(a)** As a starting point, we found that the differences in association scores were unrelated to differences in expression of its proteins between tissues across protein pairs (ρ = -0.02 ± 0.00 (mean with s.e.m.); p-value < 4e-14 for all but one pair of tissues). Shown are the pearson's correlation coefficients between the differences in association scores and the differences in log-fold changes of expression for all pairs of tissues (the log-fold change in expression for a protein pair was defined as the maximum log-fold change of the two proteins between tissues; gene expression data c.f. Fig. S13). Each dot represents one tissue. Error bar shows mean with s.e.m. (n= 55 pairs of tissues)). **(b)** Next, we selected the associations that were co-quantified for tissues A and B, and whose association score in tissue A exceeded the association score in tissue B. We then computed the percentage of these associations involving proteins whose expression in tissue B was different from the expression in tissue A by some minimum fold-change. We found that 29.3% of all associations had both lower association scores and at least 2-fold lower expression in other tissues (light red), compared to 30.2% of associations that had lower scores but at least a 2-fold higher expression in the other tissues (light blue). Similarly, we found that 10.3% (dark red) and 10.8% (dark blue) of associations lower association scores and at least 5-fold lower and 5-fold

higher expression in other tissues respectively. Thus, the associations that are more likely in one tissue compared to the other tissues are not more likely to involve proteins whose expression is higher compared to the other tissues. **(c)** As in (b), but filtering for associations with scores that were lower by some minimum amount in the other tissues. Shown is, as function of the minimum amount by which the score in tissue B is lower than the score in tissue A, the percentage of associations involving proteins whose gene expression in tissue B is at least 2-fold lower (but not zero - light red curve), 5-fold lower (dark red), 2-fold higher (light blue), or 5-fold higher (dark blue) compared to tissue A. These results demonstrate that the percentage of such associations that involved proteins with higher expression in the other tissues was generally not smaller than the percentage of associations that involved proteins with lower expression in the other tissues (p-value 0.69 - one-sided MWU-test; comparing percentages across minimum differences in score between and pairs of tissues). Finally, the same trend holds when further filtering for associations that are likely (scores > 0.5) in at least one of the tissues (p-value 0.80 - one-sided MWU-test). Together, (a-c) show that differences in association scores between tissues cannot be explained by differences in gene expression. **(d)** Finally, we considered the associations that were quantified for one tissue but not the other tissues. For pairs of tissues A and B, we computed the percentage of "unquantified associations" - protein pairs that had association scores for tissue A but not for tissue B. Taking the median across all pairs of tissues, we found that 24.4% of associations were typically not quantified in another tissue (interquartile range being 12.0% to 37.5% of associations). Similarly, we found that 26.3% of likely associations were typically not quantified in other tissues. We hypothesized that associations may be unquantified in other tissues due to the lack of gene expression for at least one of the proteins in those tissues. To test this, we determined whether the unquantified associations involved proteins that had substantial differences in gene expression between the tissues. **(e)** Specifically, we filtered for the protein pairs having association scores for tissue A but not tissue B, and computed the percentage of these associations involving proteins whose expression in tissue B was different from the expression in tissue A by some minimum fold-change. We found that 53.3% (light red) and 34.5% (dark red) of unquantified associations involved proteins whose expression was at least 2-fold and 5-fold lower in the other tissues respectively, of which 8.7% (brown) involved proteins that were not expressed in the other tissues. In contrast, only 25.1% (light blue) and 8.9% (dark blue) of all unquantified associations involved proteins whose expression was at least 2-fold and 5-fold higher in the other tissues respectively. Thus, associations that are not quantified in other tissues are more likely to have lower expression in those tissues. From these observations we estimated that roughly 28% of differences in unquantified associations between tissues could be explained by gene expression. To do so, we compared the percentages of unquantified associations involving proteins with lower or higher expression in the other tissue: 28.2% = 53.3% (>2x lower expression) - 25.1% (>2x higher expression; background). We found similar percentages when looking at larger differences in gene expression (from 5-fold difference: 34.5% - 8.9% = 25.6%). **(f)** Analogous to (c) the colored curves show, as a function of threshold score in tissue A, the percentage of protein pairs of tissue A that are not quantified in tissue B and that involve proteins whose genes are not expressed in tissue B (brown curve), or whose gene expression in tissue B is at least 2-fold lower (light red curve - including unexpressed genes), 5-fold lower (dark red), 2-fold higher (light blue), or 5-fold higher (dark blue) compared to tissue A. As before, we found that less than 30% of differences in unquantified associations between tissues could be explained by gene expression when filtering for unquantified associations that were likely (scores > 0.5; less than 27.5%) or confident (scores > 0.8; less than 29.3%).

Overall, we found that gene expression only explains differences between tissues for unquantified associations, as the differences in association scores are not driven by gene expression (a-c). Using the percentage of unquantified associations between pairs of tissues (typically 24.4% (from d)), and using the differences in unquantified associations that can be attributed to gene expression for each pair of tissues (roughly 28.2% (from e)), we find that 6.4% ± 1.0% (mean with s.e.m. for 110 pairs of tissues) of differences in all associations between pairs of tissues can be attributed to gene expression. Similarly, 6.9% ± 1.0% of differences in likely associations between pairs of tissues can be attributed to gene expression. Together, our findings suggest that the differences between tissues are not driven by gene expression, with higher association scores not being more likely to involve proteins with higher gene expression and with differences in association scores being uncorrelated with differences in gene expression (a-c). These analyses further support our previous observations that the association scores are not driven by the tissue-specificity of expression (Fig. S13). Additionally, we estimate that less than 7% of differences in (likely) associations can be explained by gene expression due to differences in expression leading to associations being quantified in one tissue but not in other tissues (d-f). In (c) and (f), the dotted lines show the average of percentages that were quantified for all pairs of tissues. In (b-c) and (e-f), the shaded areas indicate the s.e.m. (n=110 pairs of tissues).

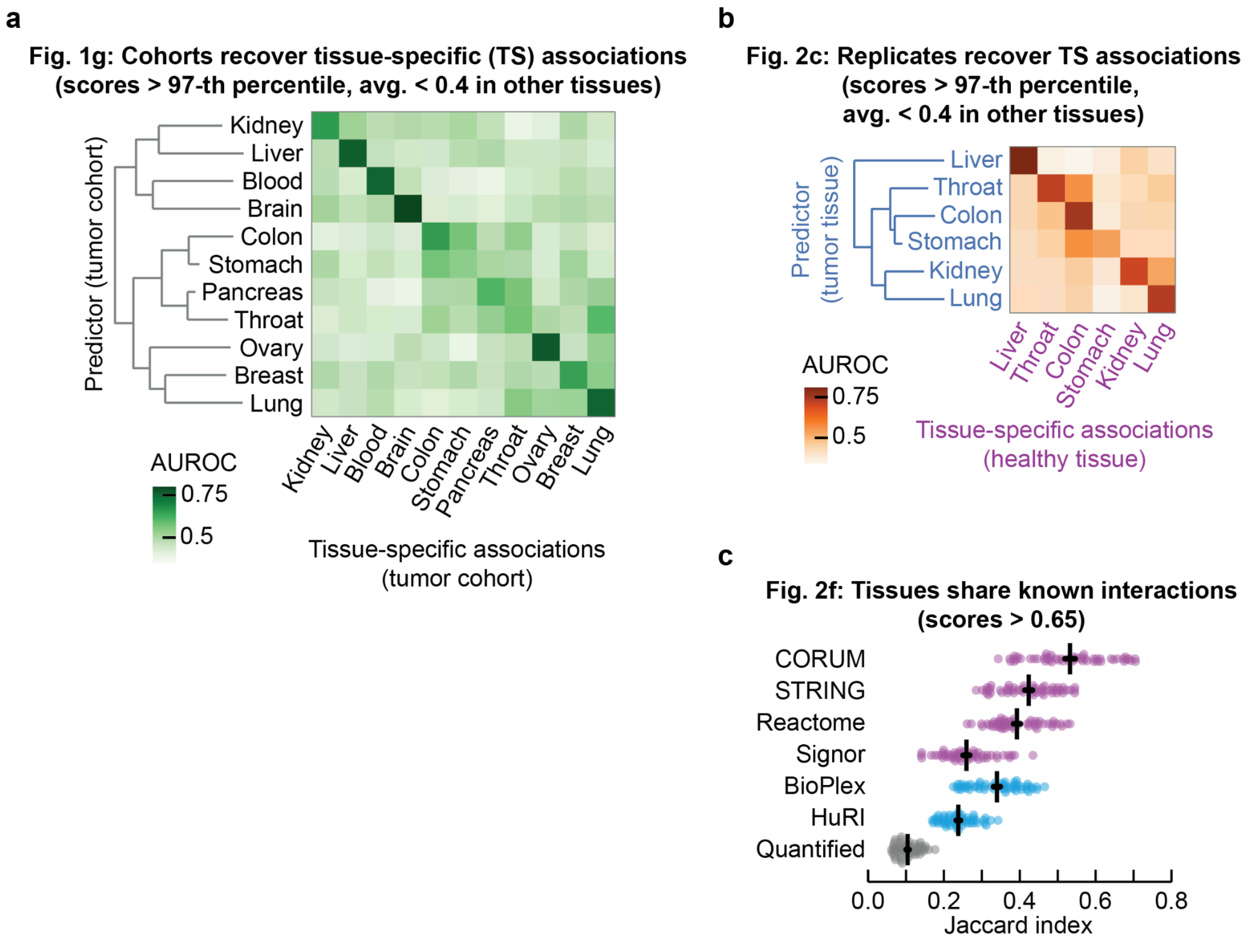

Figure S15. Analyses are not sensitive to the choice for score cut-off (related to Fig. 2). The analyses in Figs. 1-2 did not use any score cut-off to filter for associations, apart from the analyses in Fig. 1g and 2c,f. We performed the same analyses with different score cut-offs to demonstrate that the analyses are not sensitive to the cut-offs that are used. **(a)** In Fig. 1g, we defined the tissue-specific associations as having scores amongst the 95-th percentile in a given tissue with the average score remaining below 0.5 in the other tissues (Methods). The 95-th percentile corresponds to a score cut-off of 0.68 ± 0.01 (mean with s.e.m. (n=11 tissues)) across tissues. Changing the cut-off scores, we re-defined the tissue-specific associations as the associations with scores exceeding the 97-th percentile for a given tissue (corresponding to a cut-off score of 0.72 ± 0.01 across tissues), with the average score remaining below 0.4 for the other tissues. Heatmap shows the AUCs for recovering these tissue-specific associations using cohorts that were withheld when predicting the associations (Methods). Each square represents the average AUC for all cohorts of a tissue recovering the tissue-specific associations of a given tissue. We find results analogous to Fig. 1g when using different cut-off scores, with tissue-specific associations primarily being recovered by cohorts of the same tissue (AUCs 0.70 ± 0.02 (n=45)) compared to cohorts from different tissues (0.47 ± 0.00 (n=450)). **(b)** Tissue-specific associations for Fig. 2c were defined analogous to Fig. 1g. The 95-th percentile of the matched healthy- and tumor-derived scores is 0.70 ± 0.01 (mean with s.e.m. (n=6)) when averaged over all pairs. Analogous to (a), we re-defined the tissue-specific associations as the healthy-derived associations with scores exceeding the 97-th percentile for a given tissue (corresponding to a cut-off score of 0.74 ± 0.01 averaged over the tissues), with the average healthy-derived score remaining below 0.4 for the other tissues. Heatmap shows the AUCs for using tumor-derived association scores of tissues to recover tissue-specific associations predicted with the healthy-derived association scores of tissues (Methods). As before, we find results analogous to Fig. 2c when using different cut-off scores with the tissue-specific associations being recovered by the tumor-derived scores of the same tissue (AUCs 0.71 ± 0.04 (n=6)) compared to the tumor-derived scores of different tissues (0.43 ± 0.01 (n=30); p-value 2.1e-4, one-sided Welch's t-test). In (a-b), the tissues are clustered with

complete-linkage clustering using the manhattan distance. **(c)** Fig. 2f compares the recovery of known interactions having different sources of evidence using the likely associations of the tissues (scores > 0.5; Methods). Specifically, shown, are the associations (score > 0.65) that are shared between pairs of tissues as quantified by the Jaccard index (gray dots), compared to shared associations restricted to complex members (CORUM), physical associations (STRING scores exceeding 400), biological pathways (Reactome) and signaling (SIGNOR) (purple dots), or associations detected through yeast two-hybrid (HuRI) or affinity purification (BioPlex [2]) experiments (blue dots). Results are analogous to Fig. 2f. Each dot represents a pair of tissues. Error bars show mean with s.e.m. (n=55 pairs of tissues). Overall, these re-analysis demonstrate that different score cut-offs yield analogous results, with the analyses depending on the cut-off that is used.

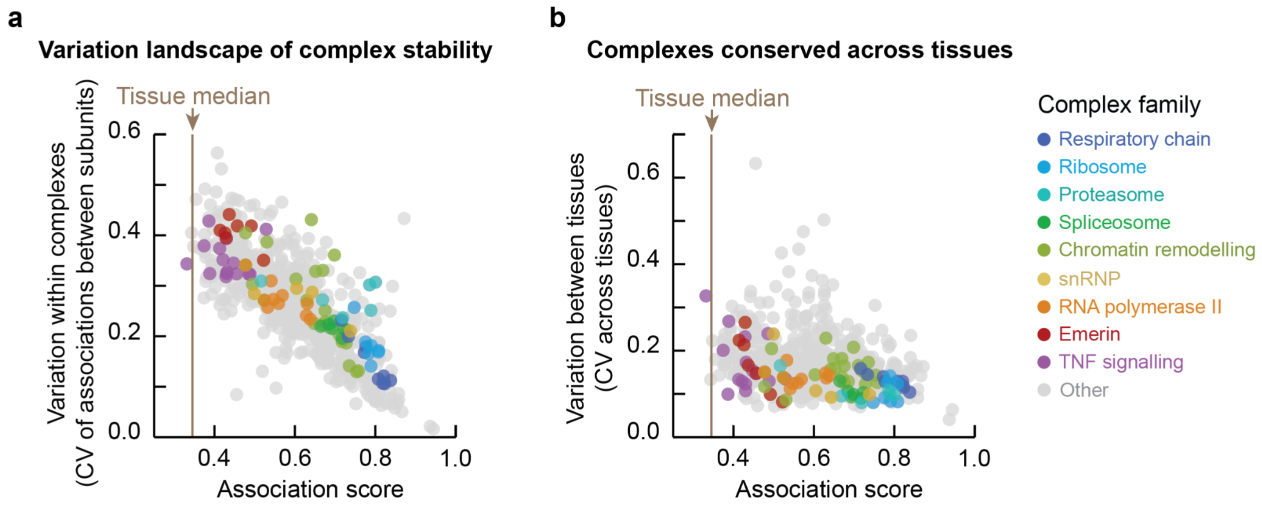

Figure S16. Well-defined human protein complexes preserved across tissues (related to Fig. 2). Given the conservation of associations between complex members across tissues, we explored the variation landscape of well-defined human protein complexes. Specifically, we filtered the human protein complexes in the CORUM database for complexes that consisted of at least 5 subunits and merged complexes with identical names. For the resulting 642 protein complexes, we explored the variation of associations within complexes and between tissues. We computed a complex-level association score (the median association score between all pairs of subunits; Table S26), a complex-level variation (the coefficient of variation (CV) of the association scores of all pairs of subunits; Table S27) and the variation of complexes between tissues (the CV of the complex-level association scores across tissues). **(a)** Complex-level variation as a function of the complex-level association scores, both averaged across tissues. We found that 76% of the protein complexes had an average complex-level association score that exceeded 0.5. Complexes became more variable as the complex-level association score decreased (ρ = -0.77, p-value 6.2e-125), suggesting that the associations of subunits become unlikely for more variable – less stable – complexes. Indeed, we found that variable complexes typically involved signaling and regulation (e.g., TNF (red) and Emerin (purple) complexes) while more stable complexes involved central cellular structures (e.g., ribosomes (light blue) and the respiratory chain (dark blue)). **(b)** Average and variation of complex-level association scores across tissues. Variation of complexes between tissues poorly correlates with the complex-level association scores (ρ = -0.28, p-value = 4.5e-13). As expected, central cellular structures such as the ribosome, respiratory chain, spliceosome, proteasome, and RNA polymerase were less variable between tissues compared to other protein complexes for which we quantified at least 5 genes in all tissues (average CV across tissues of 0.12 and 0.15 respectively, p-value 1.2e-6; one-sided MWU-test). However, we also found that complexes were not more variable across tissues than arbitrary collections of associations. Specifically, we found that there was no reason to assume that the complexes were more variable across tissues than dummy complexes consisting of arbitrary subunits and following the same size distribution (average coefficients of variation of 0.168 and 0.165 respectively; p-value = 0.24; two-sided MWU-test). To construct these dummy complexes, we randomly sampled the subunits for each CORUM complex from the pool of proteins that was quantified in all tissues (n=50 replicates) and subsampled these sampled subunits to match the number of actual subunits that were quantified for each tissue. We then computed the association score of the dummy complex as the median association score between all pairs of the sampled subunits). Together, these observations suggest that the well-defined protein complexes are generally preserved across tissues, and that there is no reason to assume tissue-specificity at the complex-level beyond what would be expected to result from tissue-specific interactions of the individual subunits to a similar degree as arbitrary pairs of proteins. In (a-b), each dot represents one protein complex. Brown solid line represents the median association score averaged over the tissues.

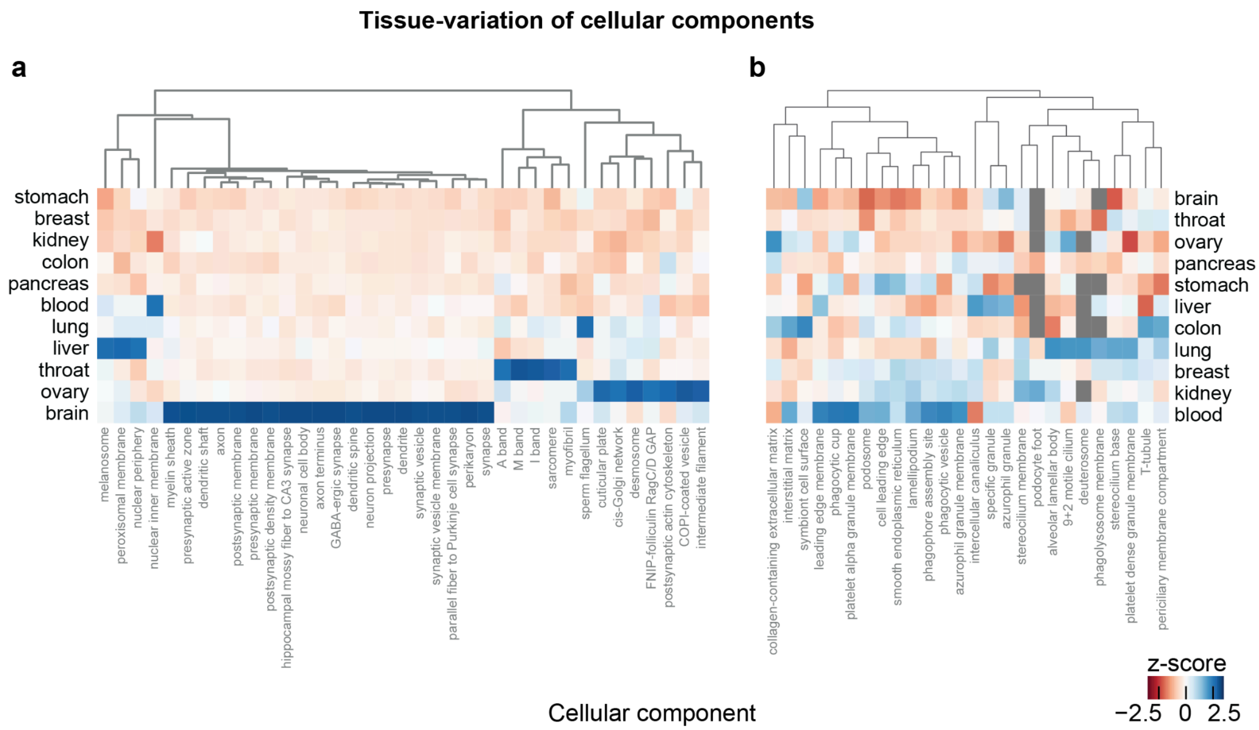

Figure S17. Protein associations vary for cell type-specific components (related to Fig. 2). Analogous to the protein complexes, for each tissue, we computed component-level association scores as the median association score between all pairs of proteins associated with cellular components as defined by Gene Ontology (Methods). **(a)** We computed the component-level association scores relative to the median association score for each tissue and z-scored these relative component-level associations across tissues. We found that 20.8% of the cellular components had z-scores exceeding 2.5 in at least one tissue. Shown are the cellular components whose z-score exceeded 2.5 in at least one tissue and that had at least 10 associations quantified in all tissues (limited to showing the 20 most tissue-specific components for each tissue (i.e., the brain)). From this systematic analysis, we found that the protein associations varied strongly for typical tissue-specific components, such as synaptic components for the brain, peroxisomal components for the liver and structural components of muscle fiber for the throat. **(b)** To gain some additional insight into cell type and tissue-specificity of cellular components, we manually curated some cellular components with tissue-elevated component-level association scores. Shown are the z-scored component-level associations across tissues. This manual curation reveals tissue-elevated components for the kidney (podocyte foot), liver (intercellular canaliculus), colon (e.g., symbiont cell surfaces), blood (e.g., for the function of the immune system - cellular motility, phagocytosis and invasion), the lung (e.g., for respiratory function - motile cilia, secretion of pulmonary surfactants). Together, these analyses demonstrate that protein-protein associations vary strongly between tissues, revealing cell-type specific differences between tissues at the level of cellular components. In (a-b), the dendrogram shows clustered go-terms using complete-linkage clustering with the Manhattan distance. Grey squares were not quantified.

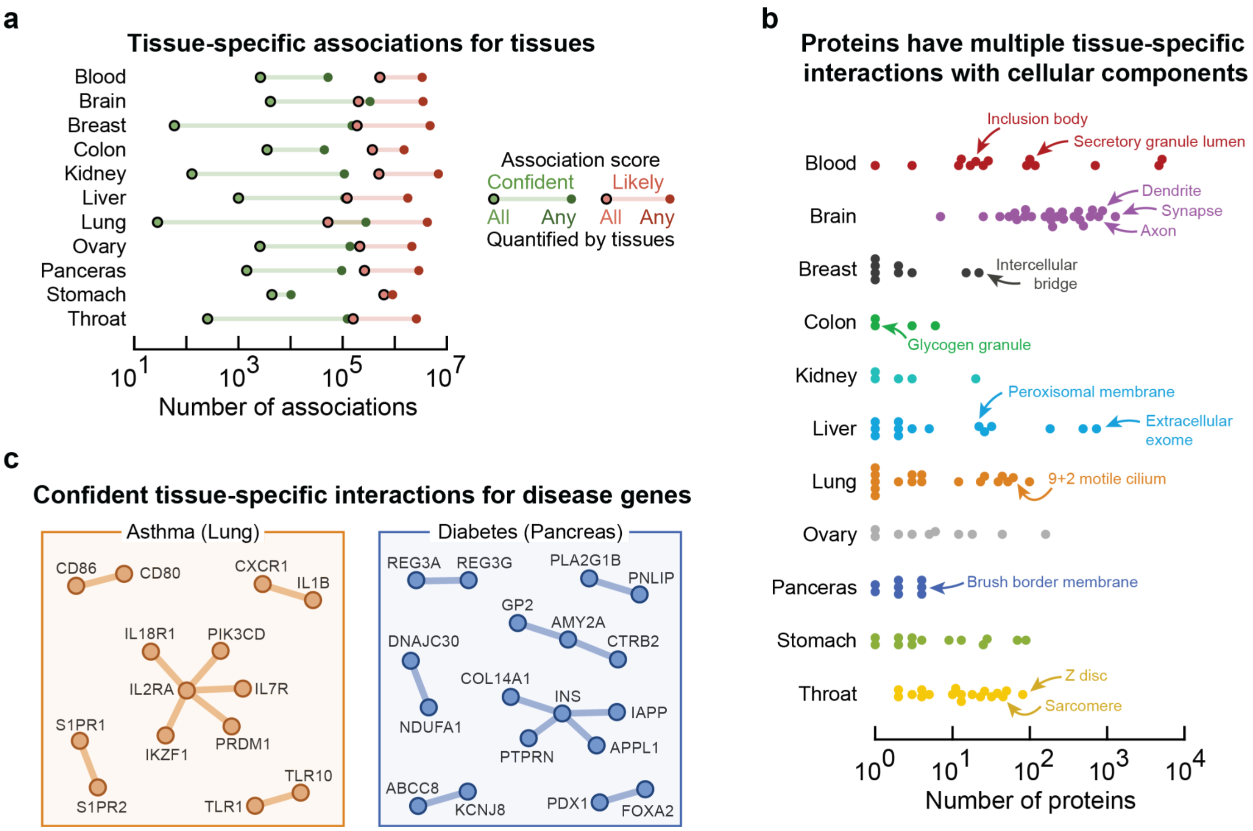

Figure S18. Tissue-specific associations for cellular components and disease (related to Fig. 3). (a) Quantifying the number of tissue-specific associations per tissue. For each tissue, we quantified the number of associations whose score exceeded some threshold for the given tissue and whose score remained below 0.5 for all other tissues. Shown is the number of such tissue-specific associations for threshold score 0.5 (likely associations - red dots) and 0.8 (confident associations - green dots). Here, we distinguished between associations that were quantified in all tissues (black edge) or associations that were quantified in any number of tissues (no edge - at least quantified for the given tissue). We found that tissues have 3.1e6 ± 5.1e5 (mean with s.e.m. of n=11 tissues) tissue-specific likely associations, of which 2.9e5 ± 5.5e4 are quantified in all tissues. Similarly, tissues have on average 1.3e5 ± 2.9e4 tissue-specific confident associations, of which 1.8e3 ± 5.1e2 are quantified in all tissues. **(b)** For each tissue, we filtered the tissue-specific likely associations for protein pairs whose interactions additionally had prior evidence (i.e., reported in STRING, HuMAP>0.1 [15], IntAct [14], or BioPlex). We then selected the top 25 cellular components whose component-level association scores (Fig. S17), relative to the median association scores of the tissues, were elevated for the tissue (z-score > 1 across tissues; average z-score 1.9 ± 0.2 across tissues). Finally, we quantified the number of proteins that had known interactions with at least two of the genes associated to these cellular components and whose interactions were tissue-specific and likely associations according to our association atlas. The resulting proteins had multiple known interactions with the components, with both the cellular components and interactions being specific for the given tissue. Shown is the number of proteins each tissue-elevated cellular component had multiple tissue-specific interactions with. Each dot represents one component. **(c)** Finally we studied some examples of tissue-specific associations for disease genes. As before (b), we filtered the tissue-specific confident associations for protein pairs whose interactions had prior evidence. Shown are the lung-specific confident interactions between proteins associated with asthma (orange) and the pancreas-specific confident interactions between proteins associated with diabetes (purple). Genes were associated with traits through GWAS (L2G >= 0.5), mouse phenotyping (IMPC scores >= 0.5) or drug targets (ChEMBL clinical stage II and higher), merging traits whose name contains the disease while ignoring cancers and measurements. Many of these known interactions are context-specific for the tissue and disease. For example, for asthma (chronic

inflammation of the airways) we found many lung-specific interactions between proteins involved in the signaling and function of the immune system (e.g., interleukins). For diabetes, we found a pancreas-specific confident association between insulin (INS) and amylin (IAPP), indeed both synthesized and co-secreted by the pancreas [30]. We further found a pancreas-specific association between subunits (KCNJ8 and ABCC8) of the beta-cell ATP-sensitive potassium channel that is involved in insulin secretion [31]. Finally, we found a pancreas-specific association between PDX1 and FOXA2, interacting transcription factors critical for pancreatic development and function [32]. These examples demonstrate cell type- and tissue-specific associations of protein complexes, cellular components and disease genes that are relevant for the given tissue within the context of disease. Overall, the results suggest that the association atlas may be used for studying the tissue-specificity of known interactions and the associations of disease genes within the context of the tissue.

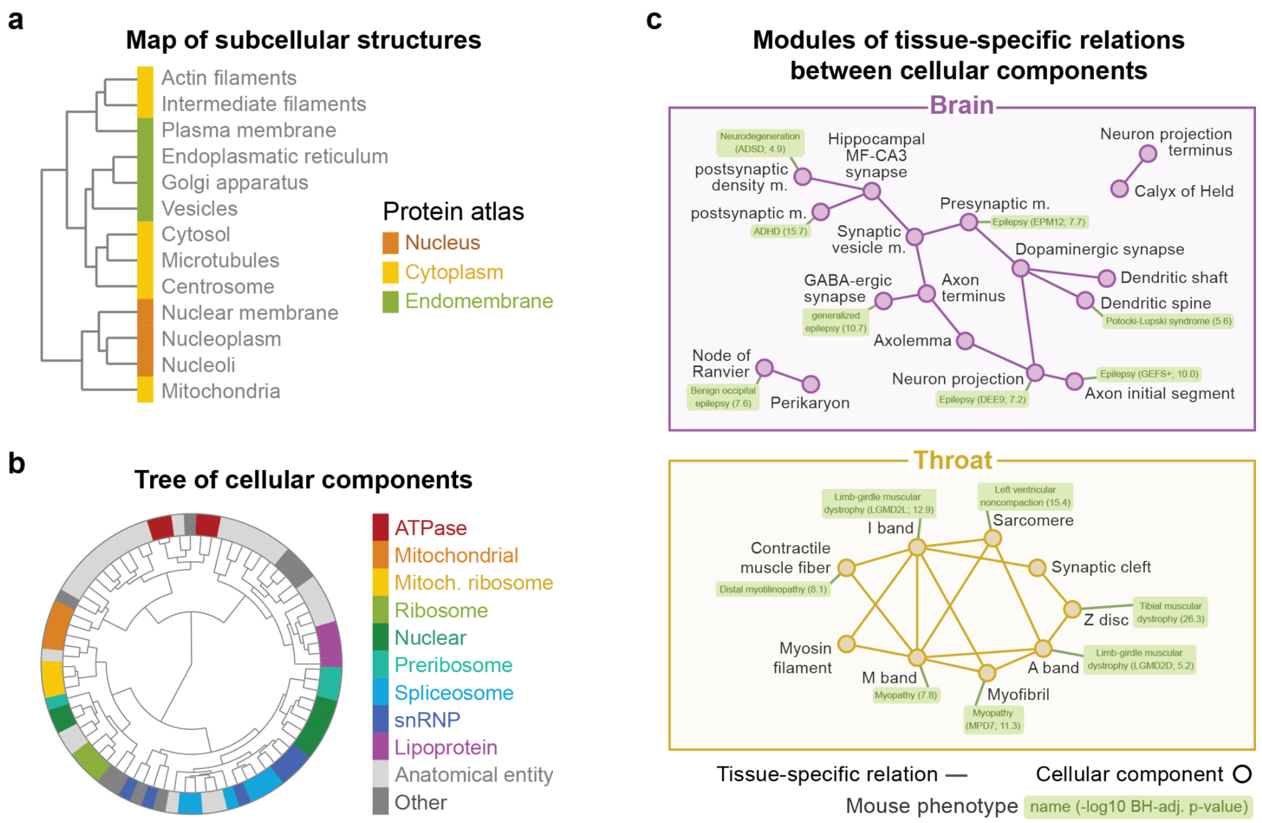

Figure S19. Mapping the organization of subcellular structures (related to Fig. 3). Using our association atlas, we sought to explore the structural organization of the human cell by systematically mapping the relations between multi-protein structures such as organelles and protein complexes in a tissue-specific manner. Most ontologies such as GO have been built and manually curated by domain experts. However, manually curating relations between ontology terms at scale and in a consistent manner has proven to be difficult. Moreover, systematically mapping the relations between ontology terms is challenging as it requires some relational information to link terms with no overlapping genes. For example, pairs of protein sets such as GO cellular components share few proteins (average Jaccard index 1.5e-3 (n=376,278)), illustrating that such gene sets are insufficient to link arbitrary pairs of ontology terms. However, as an example, one would expect that proteins that are annotated to be related with the nuclear membrane have higher association scores with proteins annotated with other nuclear-linked compartments compared to arbitrary proteins. By aggregating the association scores between pairs of proteins from different components for each tissue, we could thus build a tree of relationships that recovers the structural organization of the cell in a tissue-specific manner. We computed the relationship score between two ontology terms as the median association score between all pairs of proteins that are not shared between the terms (following Fig. 3). This methodology thus omits any similarity between terms that is simply due to the presence of common genes. **(a)** As a proof-of-concept, we sought to explore the relationships between subcellular structures as defined through cellular location data reported by the Protein Atlas. We filtered for proteins that had an unique main location and whose reliability was ‘Enhanced’ or ‘Supported’. We then determined the relationship scores between all subcellular locations in all tissues (Table S28). Finally, we averaged the relationship scores across tissues, and then clustered the locations based on their average relationship scores with all other locations to reconstruct a map of subcellular structures. Dendrogram shows the subcellular structures as defined by cellular location of proteins from the Protein Atlas (complete-linkage clustering with the Manhattan distance - leaf-joint distances are shortened for visualization). Subcellular structures are labeled as nuclear (orange), cytoplasm (green) or endomembrane (yellow) following annotation from the Protein Atlas. We found that the structures were organized based on both organellar similarity (i.e., nuclear, cytoplasmic and endomembrane) and spatial distribution (i.e., nucleus, cellular core and periphery). The relationship scores thus reveal a hierarchical organization of structures in the cell. **(b)** With the same approach, we used the relationship scores to map

the hierarchical organization of GO cellular components. Taking the component-level association scores (the median association score between all pairs of proteins associated with a GO cellular component; Tables S5-6), we selected the cellular components whose median association score, relative to the median association score of the tissues, exceeded 1.5 when averaged over the tissues (i.e., the components whose associated proteins are typically 1.5-fold more likely to associate compared to arbitrary pairs of proteins in the tissues). We then used the relationship scores between these 76 cellular components, relative to the median association score of each tissue, to compute the average relative relationship scores between each pair of components. Radial dendrogram shows hierarchical organization of the GO cellular components (complete linkage clustering with the Manhattan distance of the relationship matrix). Leaves are colored based on shared Gene Ontology names and GO ancestry for the anatomical entities (i.e., having GO:0110165 as parental node in the ontology graph). We found that cellular components with shared names were generally clustered together. Overall, the proposed methodology thus reconstructs the spatial organization of cellular structures on several size-scales ranging from protein complexes to subcellular compartments. **(c)** Finally, we used the differences in relationship scores between tissues to map the tissue-specificity of cellular components. As an example, for each tissue, we filtered for pairs of components that consisted of at least 15 proteins and whose relationship score exceeded the 99-th percentile of all pairs of components. We then selected pairs of cellular components whose relationship scores relative to the tissue median scores were tissue-specific (z-scores > 2.5 across tissues), revealing modules of high-confidence, tissue-specific relations between cellular components. Shown are modules for the brain (purple) and the throat (yellow), limited to the top 15 most tissue-specific relations for each tissue. We annotated the cellular components that were enriched with genes associated to traits through mouse phenotyping (green boxes - IMPC score >= 0.5). Here, each component shows at most one phenotype and each phenotype is shown only once, for the component having the strongest enrichment (one-sided Fisher exact test, Benjamini-Hochberg (BH) adjusted p-values < 1e-4). The brain modules had tissue-specific connections involving synaptic components that were enriched with genes associated with brain disorders. Similarly, for the throat, we found that the modules had tissue-specific connections of cellular components for muscle fiber and were enriched with genes associated with muscular disorders. Overall, these analyses demonstrate the use of our association atlas for systematically scoring the relations between cellular structures in a tissue-specific manner.

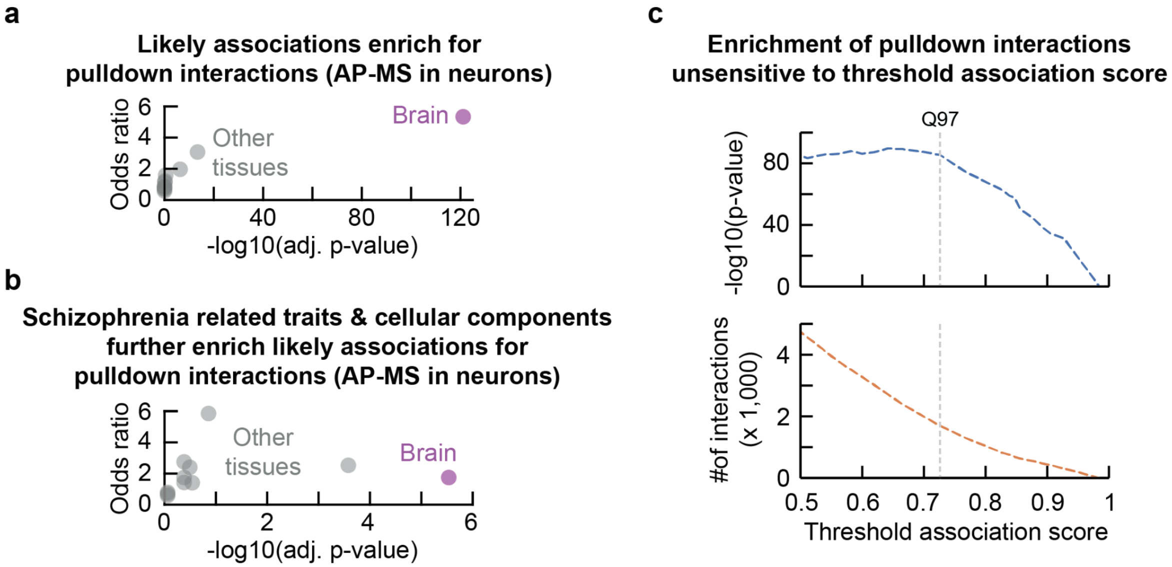

Figure S20. Brain associations of schizophrenia-related genes enriched for interactions from AP-MS in neurons (related to Fig. 4). As before, we filtered the brain interactions from pulldowns to baits that we would consider based on GWAS studies (L2G >= 0.5), and we filtered the protein associations in each tissue to only include associations that we could have detected with the pulldown studies (i.e., interactions of the bait proteins). We then tested how well the association scores could recover the brain interactions from pulldown studies in a tissue-specific manner. **(a)** First, we tested whether the most likely associations of each tissue (association scores exceeding the 97-th percentile) were enriched for these pulldown interactions. Shown is the odds-ratio as function of the Benjamini-Hochberg (BH) adjusted p-value (one-sided Fisher exact test) for the brain (purple) and the other tissues (gray). Indeed, we found that the top-percentile associations were enriched for the brain-interactions from pulldowns, especially for the brain (log BH-adj. p-value 121.1) compared to the other tissues (2.1 ± 1.4 (mean with s.e.m. of n=10 tissues)). **(b)** Next, we tested whether we could filter these most likely associations to further enrich the pulldown interactions. To do so, we selected the associations of protein pairs that involved one schizophrenia (SCZ) starting gene (L2G > 0.5) and one protein from the top 25 traits and cellular components that had the strongest tissue-specific relation to SCZ in each tissue (Methods). Here, we found that these SCZ-prioritized protein pairs were further enriched for the pulldown interactions compared to the other high-scoring (97-th percentile) associations, with the brain (log BH-adj. p-value 5.5) outperforming all other tissues (0.7 ± 0.3). We could thus use the likely associations involving SCZ-related genes and genes from SCZ-related traits and cellular components to enrich for interactions found for SCZ-related genes with pulldowns in brain cells. **(c)** Finally, we confirmed that the enrichment of pulldown interactions amongst the selected likely associations for SCZ-related genes was not sensitive to the choice of the threshold association score (i.e., the -log10 p-value exceeded 30 for all threshold association scores in the range 0.5 (all likely associations; 85-th percentile) to 0.93 (99.9-th percentile); top - blue curve), while the number of selected associations decreased roughly linear with the threshold association score (bottom - orange curve).

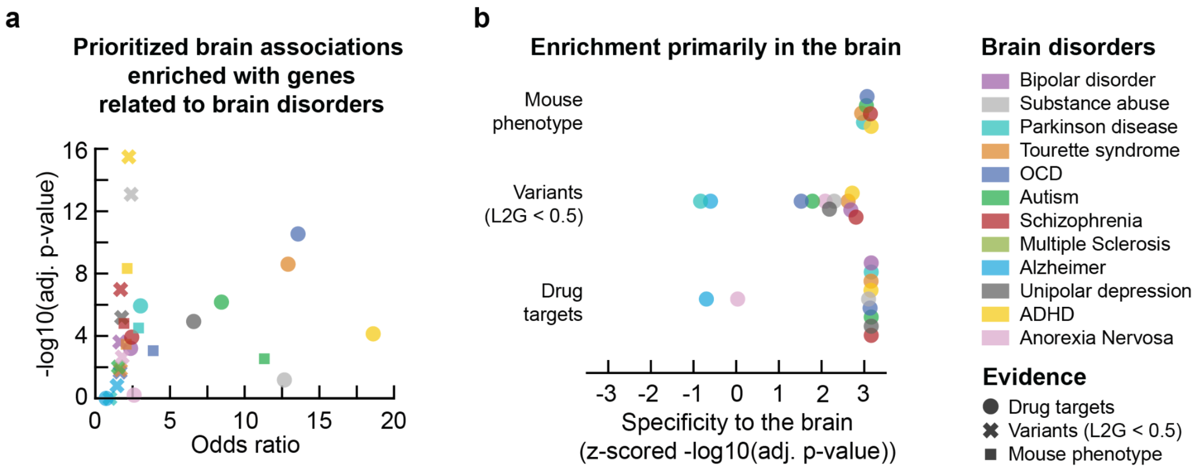

Figure S21. Proposed methodology for constructing networks of disease-related brain associations enriches for disease genes (related to Fig. 4). We selected brain-disorders from the GWAS traits whose trait-level association scores were elevated in the brain compared to the other tissues (z-score > 1; Fig. 3), that were also a mouse phenotype (IMPC) or had drug targets (ChEMBL), and whose proteins had at least 100 associations quantified in all tissues. We curated the remaining 21 traits for brain-specific disorders. Following the methodology identical to selecting the potential associations of schizophrenia-related genes (Fig. 4a-b), we created tissue-specific networks of associations for each of the resulting n=11 brain disorders. In short, we started by taking the genes associated with each disorder through GWAS studies ("starting genes" – OTAR L2G scores >= 0.5) and computed the top 25 traits and cellular components that had the strongest tissue-specific relation to the respective disorder in each tissue. We then considered as potential interactions all protein pairs that had one starting gene for a brain-disorder and one related gene for that same disorder, respectively (Methods). Next, for each tissue, we filtered these potential interactions for being in the 97-th percentile of the tissue-scores, leading to tissue-specific networks of associations for each brain-disorder. **(a)** After removing the starting genes for each disorder from the respective brain-network, the remaining genes were enriched for genes associated to the respective disorders through mouse phenotypes (-log10 BH-adjusted p-value 4.5 ± 0.6 – IMPC score >= 0.5; squares), drug targets (4.4 ± 1.0 – ChEMBL clinical stage II and above; circles), and variants with weak evidence supporting them as causal for the respective disorder (4.9 ± 1.5 – L2G scores < 0.5; crosses). Shown are the Benjamini-Hochberg (BH) adjusted p-values (mean with s.e.m.) as a function of the odds ratio for each disorder (one-sided Fisher exact test). **(b)** For most disorders (except Alzheimer, Parkinson, and anorexia nervosa), this enrichment was elevated for the brain compared to the other tissues, as demonstrated by the z-scores of the adjusted p-values for the brain (z-score > 0). This enrichment was especially striking for Tourette syndrome, schizophrenia and ADHD (z-score > 2) for all three types of evidence for gene-disease associations. Together, these observations suggest that the proposed methodology presents a systematic approach for prioritizing disease genes of tissue-specific traits.

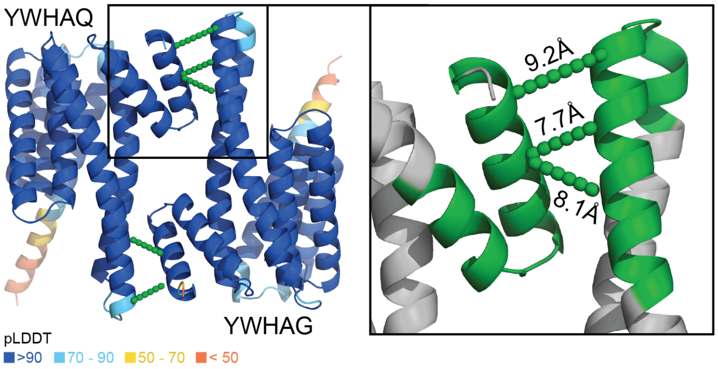

Figure S22 (related to Fig. 5). XL-MS data from mouse synaptosomes validates AlphaFold2 models of interfaces for synaptic interactions. The AlphaFold2 models of confident synaptic interactions for genes associated with brain-specific traits revealed 36 models having high-confidence predictions (ipTM > 0.7), of which 11 are known interactions (STRING scores > 750) and 9 have been found through AP-MS experiments (BioPlex score > 0.5). The 24 high-confidence predictions without any prior evidence as interactions (STRING scores = 0) are typically specific to the brain compared to other tissues (average association scores 0.69 in the synaptosome and 0.82 in the brain, compared to 0.43 in the other tissues (z-score 2.0; average ipTM score 0.78)). Finally, two of these high-confidence predictions have been identified through XL-MS in mouse synaptosomes [33]. One is the known interaction between PSMA3 and PSMA6 (ipTM = 0.77; STRING score = 999), with the two crosslinks having distances of 7.1 Å and 10.4 Å in the AF2 structure for the proteins. Similarly, the known interaction between TUBA4A and TUBB2A (ipTM = 0.90; STRING score = 991) had distances 32.3 Å and 35.2 Å for the crosslinks from the XL-MS dataset.

To further explore validated structures of our synaptic interactions (Fig. 5), we created additional AlphaFold2 structures for other synaptic interactions that we also validated with the XL-MS data from mouse synaptosomes. Specifically, we selected the confident synaptic interactions (association score > 0.8 in the brain and/or interaction probability > 0.8 in the synaptic interactome) and filtered for protein pairs that were less established interactions (STRING scores < 900), as these tend to be more tissue-specific (average z-score 1.6 in the brain; association scores) compared to the well-established ones (STRING scores >= 900; average z-score 0.6; p-value = 6e-118, one-sided t-test comparing z-scores). Finally, we filtered for the 34 protein pairs that additionally had crosslinks in the XL-MS dataset and created AF2 models of their interfaces. We found that 7 of the 33 resulting models had high-confidence predictions (ipTM > 0.7). Moreover, these high-confidence models enriched for crosslinks whose distance was below 30 Å in the model (odds ratio = 23.5; p-value = 1.4e-5; one-sided Fisher exact test - 12 of the crosslinks had distances below 30 Å and were for high-confidence models). Some of the high-confidence predictions involved well-established protein interactions, such as the 3 interactions between 14-3-3 proteins that had 9 crosslinks among them in the XL-MS dataset (average ipTM = 0.91; average distance between crosslinks = 8.2 Å). The visualized structure is the model for the interface between 14-3-3 proteins YWHAQ and YWHAG. Dotted green lines show crosslinks between peptides identified through XL-MS in mouse synaptosomes. Residues are colored by pLDDT. Inset shows distances between the identified peptides (green) of the protein (gray).

We additionally found crosslinks for the known interactions between proteins of the Collapsin response mediator family (CRMP1-DPYSL2 and DPYSL2-DPYSL3; STRING scores > 750). These proteins are important for axon guidance and dendritic growth, and have been identified as potential targets for Alzheimer's disease [34], with DPYSL2 in particular being highly expressed and specific to the brain (z-score = 5.6 when comparing expression across tissues). The AF2 model for CRMP1 and DPYSL2 (ipTM = 0.73) had distances 5.3 Å and 27.2 Å, while DPYSL2 and DPYSL3 (ipTM = 0.72) had a distance of 5.3 Å for the respective crosslinks from the XL-MS dataset. We also found moderate-confidence models (ipTM > 0.5), such as for the association of ATP2B2 with ATP1A1 (crosslink distance = 9.3 Å; ipTM = 0.58) and with ATP1A3 (distance = 9.8 Å; ipTM = 0.57). These associations are specific to the brain (association scores 0.91 and 0.95 respectively) when compared to the other tissues (0.45 and 0.58 on average respectively). Indeed, the expression of both ATP1A3 (z-score = 4.1) and ATP2B2 (z-score = 4.7) are highly specific to the brain. Intriguingly, these associations are not reported in any protein interaction database aside from HuMAP (scores < 0.006). Both ATP1A1 and ATP1A3 are the alpha subunits of Na/K-pumps, that are known to be important for regulation of intracellular calcium

and have been demonstrated to have interactions with calcium exchangers to form calcium-signaling domains in astrocytes [35]. With ATP2B2 being a calcium transporter, we hypothesize that its interactions with ATP1A1 and ATP1A3 may be related to the regulation of calcium transport in neurons. Finally, we found that the interaction between EPS15L1 and ITSN1 (STRING score 812) had a lower-confidence AF2 model (ipTM = 0.31), although the distance between the XL-MS peptides of the proteins was small (5.6 Å). Indeed, EPS15L1 is known to bind the ITSN1 coiled-coil domain, with both proteins being important for the AP-2 clathrin-coated vesicle life cycle and mouse knockout models of EPS15L1 showing severe neurological defects [36]. Overall, these observations demonstrate several synapse-specific protein associations - supported by both co-abundance in the human brain and co-fractionation in rat synaptosomes - that have moderate- or high-confidence structures and are enriched for additional evidence from XL-MS experiments in mouse synaptosomes.

REFERENCES

1. Luck, K. *et al.* A reference map of the human binary protein interactome. *Nature* **580**, 402–408 (2020).
2. Huttlin, E. L. *et al.* Dual proteome-scale networks reveal cell-specific remodeling of the human interactome. *Cell* (2021) doi:10.1016/j.cell.2021.04.011.
3. Lo Surdo, P. *et al.* SIGNOR 3.0, the SIGnaling network open resource 3.0: 2022 update. *Nucleic Acids Res.* **51**, D631–D637 (2023).
4. Milacic, M. *et al.* The Reactome Pathway Knowledgebase 2024. *Nucleic Acids Res.* **52**, D672–D678 (2024).
5. Szklarczyk, D. *et al.* STRING v11: protein-protein association networks with increased coverage, supporting functional discovery in genome-wide experimental datasets. *Nucleic Acids Res.* **47**, D607–D613 (2019).
6. Ruepp, A. *et al.* CORUM: the comprehensive resource of mammalian protein complexes--2009. *Nucleic Acids Res.* **38**, D497–501 (2010).
7. Koopmans, F. *et al.* SynGO: An Evidence-Based, Expert-Curated Knowledge Base for the Synapse. *Neuron* **103**, 217–234.e4 (2019).
8. Ashburner, M. *et al.* Gene ontology: tool for the unification of biology. The Gene Ontology Consortium. *Nat. Genet.* **25**, 25–29 (2000).
9. Hsu, Y.-H. H. *et al.* Using brain cell-type-specific protein interactomes to interpret neurodevelopmental genetic signals in schizophrenia. *iScience* **26**, 106701 (2023).
10. Pintacuda, G. *et al.* Protein interaction studies in human induced neurons indicate convergent biology underlying autism spectrum disorders. *Cell Genom* **3**, 100250 (2023).
11. O’Neill, A. C. *et al.* Spatial centrosome proteome of human neural cells uncovers disease-relevant heterogeneity. *Science* **376**, eabf9088 (2022).
12. Drummond, E. *et al.* Phosphorylated tau interactome in the human Alzheimer’s disease brain. *Brain* **143**, 2803–2817 (2020).

13. Tracy, T. E. *et al.* Tau interactome maps synaptic and mitochondrial processes associated with neurodegeneration. *Cell* **185**, 712–728.e14 (2022).

14. Orchard, S. *et al.* The MIntAct project--IntAct as a common curation platform for 11 molecular interaction databases. *Nucleic Acids Res.* **42**, D358–63 (2014).

15. Drew, K., Wallingford, J. B. & Marcotte, E. M. hu.MAP 2.0: integration of over 15,000 proteomic experiments builds a global compendium of human multiprotein assemblies. *Mol. Syst. Biol.* **17**, e10016 (2021).

16. van Oostrum, M. *et al.* The proteomic landscape of synaptic diversity across brain regions and cell types. *Cell* **186**, 5411–5427.e23 (2023).

17. Uhlén, M. *et al.* Proteomics. Tissue-based map of the human proteome. *Science* **347**, 1260419 (2015).

18. Hein, M. Y. *et al.* A human interactome in three quantitative dimensions organized by stoichiometries and abundances. *Cell* **163**, 712–723 (2015).

19. Lapek, J. D., Jr *et al.* Detection of dysregulated protein-association networks by high-throughput proteomics predicts cancer vulnerabilities. *Nat. Biotechnol.* **35**, 983–989 (2017).

20. Roumeliotis, T. I. *et al.* Genomic Determinants of Protein Abundance Variation in Colorectal Cancer Cells. *Cell Rep.* **20**, 2201–2214 (2017).

21. Kustatscher, G. *et al.* Co-regulation map of the human proteome enables identification of protein functions. *Nat. Biotechnol.* **37**, 1361–1371 (2019).

22. Coscia, F. *et al.* Integrative proteomic profiling of ovarian cancer cell lines reveals precursor cell associated proteins and functional status. *Nat. Commun.* **7**, 12645 (2016).

23. Grundner-Culemann, K. *et al.* Comparative proteome analysis across non-small cell lung cancer cell lines. *J. Proteomics* **130**, 1–10 (2016).

24. Nusinow, D. P. *et al.* Quantitative proteomics of the Cancer Cell Line Encyclopedia. *Cell* **180**, 387–402.e16 (2020).

25. Lichti, C. F. *et al.* The proteomic landscape of glioma stem-like cells. *EuPA Open Proteom.* **8**, 85–93 (2015).

26. Emdal, K. B. *et al.* Phosphoproteomics of primary AML patient samples reveals rationale for AKT combination therapy and p53 context to overcome selinexor resistance. *Cell Rep.* **40**, 111177 (2022).

27. Leo, I. R. *et al.* Integrative multi-omics and drug response profiling of childhood acute lymphoblastic leukemia cell lines. *Nat. Commun.* **13**, 1691 (2022).

28. Luca, B. A. *et al.* Atlas of clinically distinct cell states and ecosystems across human solid tumors. *Cell* **184**, 5482–5496.e28 (2021).

29. Cerami, E. *et al.* The cBio cancer genomics portal: an open platform for exploring multidimensional cancer

genomics data. *Cancer Discov.* **2**, 401–404 (2012).

30. Madsen, O. D. *et al.* Islet amyloid polypeptide and insulin expression are controlled differently in primary and transformed islet cells. *Mol. Endocrinol.* **5**, 143–148 (1991).

31. De Franco, E. *et al.* Update of variants identified in the pancreatic β-cell KATP channel genes KCNJ11 and ABCC8 in individuals with congenital hyperinsulinism and diabetes. *Hum. Mutat.* **41**, 884–905 (2020).

32. Ebrahim, N., Shakirova, K. & Dashinimaev, E. PDX1 is the cornerstone of pancreatic β-cell functions and identity. *Front. Mol. Biosci.* **9**, 1091757 (2022).

33. Gonzalez-Lozano, M. A. *et al.* Stitching the synapse: Cross-linking mass spectrometry into resolving synaptic protein interactions. *Sci Adv* **6**, eaax5783 (2020).

34. Quach, T. T. *et al.* Collapsin response mediator proteins: Novel targets for Alzheimer's disease. *J. Alzheimers. Dis.* **77**, 949–960 (2020).

35. Tian, J. & Xie, Z.-J. The Na-K-ATPase and calcium-signaling microdomains. *Physiology (Bethesda)* **23**, 205–211 (2008).

36. Mishra, R., Sengül, G. F., Candiello, E. & Schu, P. Synaptic AP2 CCV life cycle regulation by the Eps15, ITSN1, Sgip1/AP2, synaptojanin1 interactome. *Sci. Rep.* **11**, 8007 (2021).
