# Supplementary material for: A tissue-specific atlas of protein–protein associations enables prioritization of candidate disease genes
Source: Nat Biotechnol. 2025 May 2;44(4):654–67. doi: 10.1038/s41587-025-02659-z (PMC13090126; doi:10.1038/s41587-025-02659-z)
Supplement: Supplementary file 2 — Reporting Summary [file 41587_2025_2659_MOESM2_ESM.pdf]

nature portfolio

Corresponding author(s): PB

Last updated by author(s): Mar 21, 2025

Reporting Summary

Nature Portfolio wishes to improve the reproducibility of the work that we publish. This form provides structure for consistency and transparency in reporting. For further information on Nature Portfolio policies, see our Editorial Policies and the Editorial Policy Checklist.

Statistics

For all statistical analyses, confirm that the following items are present in the figure legend, table legend, main text, or Methods section.

n/a	Confirmed	
☐	☒	The exact sample size (*n*) for each experimental group/condition, given as a discrete number and unit of measurement
☐	☒	A statement on whether measurements were taken from distinct samples or whether the same sample was measured repeatedly
☐	☒	The statistical test(s) used AND whether they are one- or two-sided<br>*Only common tests should be described solely by name; describe more complex techniques in the Methods section.*
☐	☒	A description of all covariates tested
☐	☒	A description of any assumptions or corrections, such as tests of normality and adjustment for multiple comparisons
☐	☒	A full description of the statistical parameters including central tendency (e.g. means) or other basic estimates (e.g. regression coefficient) AND variation (e.g. standard deviation) or associated estimates of uncertainty (e.g. confidence intervals)
☐	☒	For null hypothesis testing, the test statistic (e.g. *F*, *t*, *r*) with confidence intervals, effect sizes, degrees of freedom and *P* value noted<br>*Give P values as exact values whenever suitable.*
☒	☐	For Bayesian analysis, information on the choice of priors and Markov chain Monte Carlo settings
☒	☐	For hierarchical and complex designs, identification of the appropriate level for tests and full reporting of outcomes
☐	☒	Estimates of effect sizes (e.g. Cohen's *d*, Pearson's *r*), indicating how they were calculated

Our web collection on statistics for biologists contains articles on many of the points above.

Software and code

Policy information about availability of computer code

Data collection	Not applicable
Data analysis	All code for data processing, analysis and the figures in this study is publicly available as Jupyter notebooks (BioStudies S-BSST1423 - https://doi.org/10.6019/S-BSST1423). Software used: AlphaFold-multimer (2.3.1), pandas (2.2.2), numpy (1.26.4), scipy (1.10.1), scikit-learn (1.6.1), networkx (3.2.1), matplotlib (3.7.2), seaborn (0.13.2), pickle (4.0), bravado (11.0.3), pyvis (0.3.1), statsmodels (0.14.0), pyspark (3.5.1), fastparquet (0.5.0), jupyter notebook (7.0.8), python (3.9.16), Spectronaut (13)

For manuscripts utilizing custom algorithms or software that are central to the research but not yet described in published literature, software must be made available to editors and reviewers. We strongly encourage code deposition in a community repository (e.g. GitHub). See the Nature Portfolio guidelines for submitting code & software for further information.

Data

Policy information about availability of data

All manuscripts must include a data availability statement. This statement should provide the following information, where applicable:

- Accession codes, unique identifiers, or web links for publicly available datasets
- A description of any restrictions on data availability
- For clinical datasets or third party data, please ensure that the statement adheres to our policy

All processed, analysed, and generated data in this study is publicly available (BioStudies S-BSST1423). The mass spectrometry proteomics data have been deposited

to the PRIDE repository (identifier PXD049084). Other proteomics data was collected from public studies (Table S1). Approved HGNC gene symbols were taken from (https://www.genenames.org), and gene symbols were harmonized or converted to human orthologs with Ensembl (https://www.ensembl.org). Evidence for protein interactions was collected from STRING (https://string-db.org), CORUM (https://www.helmholtz-munich.de), HuMAP2 (http://humap2.proteincomplexes.org), HuRI (http://www.interactome-atlas.org), BioPlex (https://bioplex.hms.harvard.edu), IntAct (https://www.ebi.ac.uk/intact), Reactome (https://reactome.org), and Signor (https://signor.uniroma2.it). Evidence for disease-related genes was obtained from The Open Targets genetics platform (https://platform.opentargets.org/). Gene ontology annotations were collected for SynGO (https://www.syngoportal.org) and for other ontology terms (https://geneontology.org), and associations of genes with GO terms were collected using the Uniprot rest API (https://www.uniprot.org) and EBI (https://www.ebi.ac.uk/QuickGO). Mutation frequencies in cancer were collected from cBioPortal (https://www.cbioportal.org). Consensus RNA expression data (https://www.proteinatlas.org/humanproteome/tissue/data#consensus_tissues_rna) and protein location data (https://www.proteinatlas.org/humanproteome/subcellular/data#locations) were collected from the Protein Atlas (https://www.proteinatlas.org). Source data are provided.

Research involving human participants, their data, or biological material

Policy information about studies with human participants or human data. See also policy information about sex, gender (identity/presentation), and sexual orientation and race, ethnicity and racism.

Reporting on sex and gender	Not applicable
Reporting on race, ethnicity, or other socially relevant groupings	Not applicable
Population characteristics	Not applicable
Recruitment	Not applicable
Ethics oversight	Not applicable

Note that full information on the approval of the study protocol must also be provided in the manuscript.

Field-specific reporting

Please select the one below that is the best fit for your research. If you are not sure, read the appropriate sections before making your selection.

☒ Life sciences ☐ Behavioural & social sciences ☐ Ecological, evolutionary & environmental sciences

For a reference copy of the document with all sections, see nature.com/documents/nr-reporting-summary-flat.pdf

Life sciences study design

All studies must disclose on these points even when the disclosure is negative.

Sample size	No sample size-calculation was performed. Cohorts of MS proteomics were selected based on the available public proteomics datasets in cancer patients at the time of performing the study. The number fractions for the mass spectrometry proteomics data (n=75) exceeds the minimum fractions (n=30) used for computing co-fractionation of proteins.
Data exclusions	The mass spectrometry proteomics data was filtered to exclude proteins without measurements and fractions with intensities below a pre-defined threshold (detailed in the Methods section - see 'Pre-processing of synaptosome co-fractionation data').
Replication	Not applicable for this study. This study is a meta-analysis of publicly available data. All findings can be reproduced through publicly available code and data, available through BioStudies (https://doi.org/10.6019/S-BSST1423). Findings are validated by independent (publicly available) experimental mass spectrometry experiments.
Randomization	Not applicable for this study. This study is a meta-analysis of publicly available data. Samples are proteomics data of cancer patients labeled with clinical information and already allocated into experimental groups. Batch effects are controlled by calibrating to a gold-standard set of positives.
Blinding	Not applicable for this study. This study is a meta-analysis of publicly available data. Samples are proteomics data of cancer patients labeled with clinical information and already allocated into experimental groups.

Reporting for specific materials, systems and methods

We require information from authors about some types of materials, experimental systems and methods used in many studies. Here, indicate whether each material, system or method listed is relevant to your study. If you are not sure if a list item applies to your research, read the appropriate section before selecting a response.

Materials & experimental systems

n/a	Involved in the study
☒	☐ Antibodies
☒	☐ Eukaryotic cell lines
☒	☐ Palaeontology and archaeology
☐	☒ Animals and other organisms
☒	☐ Clinical data
☒	☐ Dual use research of concern
☒	☐ Plants

Methods

n/a	Involved in the study
☒	☐ ChIP-seq
☒	☐ Flow cytometry
☒	☐ MRI-based neuroimaging

Animals and other research organisms

Policy information about studies involving animals; ARRIVE guidelines recommended for reporting animal research, and Sex and Gender in Research

Laboratory animals	Synaptosomes from cortices of adult rats (Rattus norvegicus; Sprague–Dawley)
Wild animals	Not applicable
Reporting on sex	Not applicable
Field-collected samples	Not applicable
Ethics oversight	All animal experiments were carried out under institutional guidelines (ZH172/18 Kanton Zürich Gesundheitsdirektion Veterinäramt).

Note that full information on the approval of the study protocol must also be provided in the manuscript.

Plants

Seed stocks	*Report on the source of all seed stocks or other plant material used. If applicable, state the seed stock centre and catalogue number. If plant specimens were collected from the field, describe the collection location, date and sampling procedures.*
Novel plant genotypes	*Describe the methods by which all novel plant genotypes were produced. This includes those generated by transgenic approaches, gene editing, chemical/radiation-based mutagenesis and hybridization. For transgenic lines, describe the transformation method, the number of independent lines analyzed and the generation upon which experiments were performed. For gene-edited lines, describe the editor used, the endogenous sequence targeted for editing, the targeting guide RNA sequence (if applicable) and how the editor was applied.*
Authentication	*Describe any authentication procedures for each seed stock used or novel genotype generated. Describe any experiments used to assess the effect of a mutation and, where applicable, how potential secondary effects (e.g. second site T-DNA insertions, mosiacism, off-target gene editing) were examined.*
